# Global burden of respiratory infections associated with seasonal influenza in children under 5 years in 2018: a systematic review and modelling study

**DOI:** 10.1016/S2214-109X(19)30545-5

**Published:** 2020-02-20

**Authors:** Xin Wang, You Li, Katherine L O'Brien, Shabir A Madhi, Marc-Alain Widdowson, Peter Byass, Saad B Omer, Qalab Abbas, Asad Ali, Alberta Amu, Eduardo Azziz-Baumgartner, Quique Bassat, W Abdullah Brooks, Sandra S Chaves, Alexandria Chung, Cheryl Cohen, Marcela Echavarria, Rodrigo A Fasce, Angela Gentile, Aubree Gordon, Michelle Groome, Terho Heikkinen, Siddhivinayak Hirve, Jorge H Jara, Mark A Katz, Najwa Khuri-Bulos, Anand Krishnan, Oscar de Leon, Marilla G Lucero, John P McCracken, Ainara Mira-Iglesias, Jennifer C Moïsi, Patrick K Munywoki, Millogo Ourohiré, Fernando P Polack, Manveer Rahi, Zeba A Rasmussen, Barbara A Rath, Samir K Saha, Eric AF Simões, Viviana Sotomayor, Somsak Thamthitiwat, Florette K Treurnicht, Marylene Wamukoya, Lay-Myint Yoshida, Heather J Zar, Harry Campbell, Harish Nair

**Affiliations:** a Centre for Global Health, Usher Institute, Edinburgh Medical School, University of Edinburgh, Edinburgh, UK; b Department of International Health, International Vaccine Access Center, Johns Hopkins Bloomberg School of Public Health, Baltimore, MD, USA; c Medical Research Council: Respiratory and Meningeal Pathogens Research Unit; Department of Science and Technology/National Research Foundation: Vaccine Preventable Diseases, Faculty of Health Sciences, University of the Witwatersrand, Johannesburg, South Africa; d Division of Global Health Protection, Center for Global Health, Centers for Disease Control and Prevention, Nairobi, Kenya; e Institute of Tropical Medicine, Antwerp, Belgium; f Department of Epidemiology and Global Health, Umeå University, Umeå, Sweden; g Yale Institute for Global Health; Section of Infectious Diseases, Department of Medicine, Yale School of Medicine; Department of Epidemiology of Microbial Diseases, Yale School of Public Health, New Haven, CT, USA; h Department of Pediatrics and Child Health, Aga Khan University, Karachi, Pakistan; i Dodowa Health Research Centre, Dodowa, Ghana; j Influenza Division, Centers for Disease Control and Prevention, Atlanta, GA, USA; k Barcelona Global Health Institute, Hospital Clínic—University of Barcelona, Barcelona, Spain; l Centro de Investigação em Saúde de Manhiça, Maputo, Mozambique; m Institució Catalana de Recerca i Estudis Avançats, Barcelona, Spain; n Pediatric Infectious Diseases Unit, Pediatrics Department, Hospital Sant Joan de Déu (University of Barcelona), Barcelona, Spain; o Consorcio de Investigación Biomédica en Red de Epidemiología y Salud Pública, Madrid, Spain; p Influenza Program, Centers for Disease Control and Prevention, Nairobi, Kenya; q Centre for Respiratory Disease and Meningitis, National Institute for Communicable Diseases, Johannesburg, South Africa; r School of Public Health, Faculty of Health Sciences, University of the Witwatersrand, Johannesburg, South Africa; s Clinical Virology Unit, Centro de Educación Médica e Investigaciones Clínicas, Argentina; t Public Health Institute of Chile, Región Metropolitana, Chile; u Ricardo Gutierrez Children Hospital, Buenos Aires, Argentina; v Department of Epidemiology, School of Public Health, University of Michigan, Ann Arbor, MI, USA; w Department of Pediatrics, University of Turku and Turku University Hospital, Finland; x Vadu Rural Health program, KEM Hospital Research Centre, Pune, Maharashtra, India; y Center for Health Studies, Universidad del Valle de Guatemala, Guatemala City, Guatemala; z Chief Physician's Office, Clalit Health Services, Clalit Research Institute, Tel Aviv, Israel; aa Ben Gurion University of the Negev, School of Public Health and Medical School for International Health, Beer-Sheva, Israel; ab University of Michigan School of Public Health, Ann Arbor, MI, USA; ac Department of Pediatrics, University of Jordan School of Medicine, Amman, Jordan; ad Centre for Community Medicine, All India Institute of Medical Sciences, New Delhi, India; ae ARI Study Group, Research Institute for Tropical Medicine, Muntinlupa, Philippines; af Área de Investigación en Vacunas, Fundación para el Fomento de la Investigación Sanitaria y Biomédica de la Comunitat Valenciana (Salud Pública), Valencia, Spain; ag Agence de Médecine Préventive, Paris, France; ah KEMRI-Wellcome Trust Research Programme, Kilifi, Kenya; ai Centre de Recherche en Santé de Nouna, Nouna, Burkina Faso; aj Fundacion INFANT, Buenos Aires, Argentina; ak Fogarty International Center, National Institutes of Health, Bethesda, MD, USA; al Vienna Vaccine Safety Initiative, Berlin, Germany; am Department of Microbiology, Child Health Research Foundation, Dhaka, Bangladesh; an Department of Pediatrics, Section of Infectious Diseases, University of Colorado, School of Medicine, Aurora, CO, USA; ao Department of Epidemiology and Center for Global Health, Colorado School of Public Health, Aurora CO, USA; ap Epidemiology Department, Ministry of Health, Santiago, Chile; aq Division of Global Health Protection, Thailand Ministry of Public Health; US CDC Collaboration, Nonthaburi, Thailand; ar Department of Medical Virology, National Health Laboratory Service and School of Pathology, Faculty of Health Sciences, University of the Witwatersrand, Johannesburg, South Africa; as African Population & Health Research Center, Nairobi, Kenya; at Department of Pediatric Infectious Diseases, Institute of Tropical Medicine, Nagasaki University, Nagasaki, Japan; au Department of Paediatrics & Child Health and Medical Research Council unit on Child & Adolescent Health, University of Cape Town, Cape Town, South Africa

## Abstract

**Background:**

Seasonal influenza virus is a common cause of acute lower respiratory infection (ALRI) in young children. In 2008, we estimated that 20 million influenza-virus-associated ALRI and 1 million influenza-virus-associated severe ALRI occurred in children under 5 years globally. Despite this substantial burden, only a few low-income and middle-income countries have adopted routine influenza vaccination policies for children and, where present, these have achieved only low or unknown levels of vaccine uptake. Moreover, the influenza burden might have changed due to the emergence and circulation of influenza A/H1N1pdm09. We aimed to incorporate new data to update estimates of the global number of cases, hospital admissions, and mortality from influenza-virus-associated respiratory infections in children under 5 years in 2018.

**Methods:**

We estimated the regional and global burden of influenza-associated respiratory infections in children under 5 years from a systematic review of 100 studies published between Jan 1, 1995, and Dec 31, 2018, and a further 57 high-quality unpublished studies. We adapted the Newcastle-Ottawa Scale to assess the risk of bias. We estimated incidence and hospitalisation rates of influenza-virus-associated respiratory infections by severity, case ascertainment, region, and age. We estimated in-hospital deaths from influenza virus ALRI by combining hospital admissions and in-hospital case-fatality ratios of influenza virus ALRI. We estimated the upper bound of influenza virus-associated ALRI deaths based on the number of in-hospital deaths, US paediatric influenza-associated death data, and population-based childhood all-cause pneumonia mortality data in six sites in low-income and lower-middle-income countries.

**Findings:**

In 2018, among children under 5 years globally, there were an estimated 109·5 million influenza virus episodes (uncertainty range [UR] 63·1–190·6), 10·1 million influenza-virus-associated ALRI cases (6·8–15·1); 870 000 influenza-virus-associated ALRI hospital admissions (543 000–1 415 000), 15 300 in-hospital deaths (5800–43 800), and up to 34 800 (13 200–97 200) overall influenza-virus-associated ALRI deaths. Influenza virus accounted for 7% of ALRI cases, 5% of ALRI hospital admissions, and 4% of ALRI deaths in children under 5 years. About 23% of the hospital admissions and 36% of the in-hospital deaths were in infants under 6 months. About 82% of the in-hospital deaths occurred in low-income and lower-middle-income countries.

**Interpretation:**

A large proportion of the influenza-associated burden occurs among young infants and in low-income and lower middle-income countries. Our findings provide new and important evidence for maternal and paediatric influenza immunisation, and should inform future immunisation policy particularly in low-income and middle-income countries.

**Funding:**

WHO; Bill & Melinda Gates Foundation.

## Introduction

Acute lower respiratory infections (ALRIs) are one of the leading causes of morbidity and mortality in children globally, accounting for 16% of mortality in children under 5 years in 2015.^1^ We previously estimated that, in 2015, 138 million ALRI cases, 22 million cases of severe ALRI, and 0·9 million ALRI deaths occurred globally.^2^ Seasonal influenza virus is an important pathogen associated with ALRI, detected in 3–7% of ALRI hospital admissions in children under 5 years.^3^, ^4^ We previously estimated that 20 million episodes of influenza-virus-associated ALRI, 1 million episodes of severe influenza-virus-associated ALRI and 28 000–111 000 influenza-virus-associated ALRI deaths occurred in children under 5 years in 2008.^5^ Despite this substantial burden, only 10% of low-income countries (LICs) and lower-middle-income countries (LMICs) have adopted national maternal or child influenza immunisation policies (13% for pregnant women) and, where present, most have achieved only low or unknown levels of vaccine uptake.^6^, ^7^, ^8^, ^9^ Influenza burden might have changed since we calculated these previous estimates due to the emergence and circulation of influenza A/H1N1pdm09. We aimed to incorporate new data to estimate the global number of cases, hospitalisations, and mortality from influenza-virus-associated ALRI in children under 5 years in 2018. These updated global burden estimates should inform the development of influenza immunisation policies and resource allocation decisions for young children.

**Research in context**

**Evidence before this study**

We previously estimated that in 2008, influenza virus was associated with 13% of all childhood acute lower respiratory infections ALRI episodes, and 2–10% of all childhood ALRI deaths, with 99% of these deaths occurring in developing countries. Since then, additional studies have estimated influenza-virus-associated or influenza-virus-attributable morbidity and mortality burden in children under 5 years globally using different methods. One systematic review (by the Global Respiratory Hospitalizations—Influenza Proportion Positive Working Group) estimated that there were 870 000 (95% CI 610 000–1 237 000) influenza-virus-associated ALRI hospitalisations in 2012 by applying the percentage of hospitalised children with ALRI positive for influenza virus to all-cause ALRI hospitalisations. Another study (by the Global Seasonal Influenza-associated Mortality Collaborator Network) estimated that there were 44 888 (95% credible interval 9243–105 690) influenza-virus-associated respiratory deaths in 2015 by use of excess respiratory mortality regression models on vital deaths records and influenza surveillance data. The third study (by the Institute for Health Metrics and Evaluation) estimated that there were about 8 million influenza-virus-attributable ALRI cases, 2 200 000 influenza-virus-attributable ALRI hospitalisations, and 23 400 influenza-virus-attributable ALRI deaths in children under 5 years in 2017; the mortality estimate was modelled on all-cause ALRI deaths, the population attributable fraction for influenza virus in ALRI, and the ratio of case-fatality between viral ALRI and bacterial ALRI.

**Added value of this study**

Our review was based on laboratory-confirmed influenza infection morbidity and mortality data. We incorporated an increased number of studies (157 studies in total, of which 78% were new data) compared to our previous analysis in 2008. Moreover, 36% of these studies were high quality unpublished data, and 39% from low-income and lower-middle income countries. The emergence and circulation of influenza A/H1N1pdm09 might have impacted the influenza burden globally. Inclusion of new data enabled us to provide new evidence in the era with the new influenza virus' circulation, informing future immunisation policy, especially in low-income and lower middle-income countries. In this review, we reported influenza-virus-associated respiratory disease burden by severity and by World Bank income level for age groups relevant for vaccine policy decisions. We estimated that influenza virus was associated with 7% of ALRI cases (10·1 million cases [uncertainty range (UR) 6·8–15·1]), 5% of ALRI hospitalisations (870 000 [UR 543 000–1 415 000]), and 4% of ALRI deaths (34 800 [UR 13 200–97 200]) in children under 5 years. About 23% of hospitalisations and 36% of in-hospital deaths were in infants younger than 6 months. About 82% of in-hospital deaths occurred in low-income and lower-middle income countries.

**Implications of all the available evidence**

Our updated review reports new influenza-virus-associated ALRI morbidity and mortality estimates in the era with the circulation of influenza A/H1N1pdm09. A large proportion of influenza-associated burden occurs among young infants and among children in low-income and lower-middle-income countries. Our findings provide new and important evidence for maternal and paediatric influenza immunisation, and should inform future immunisation policy particularly in low-income and middle-income countries.

## Methods

### Systematic review

We did a systematic review of the influenza-virus-associated disease burden in children under 5 years (appendix pp 4–6). We updated our previous review by searching Medline (Ovid), Embase (Ovid), Global Health (Ovid), CINAHL, Web of Science, and the Global Health Library for studies published between Jan 1, 2009 and Dec 31, 2018; earlier studies published from 1995 onwards in the previous review were also included.^5^ We also did our systematic review in three Chinese databases (CNKI, Wanfang, and Chongqing VIP), searching for studies published between Jan 1, 1995, and Dec 31, 2018. To search the grey literature, we also did broad searches through Google. No language or publication restrictions were applied, and reviewers screened the titles and abstracts for eligibility, and extracted data independently. Articles in non-Chinese or non-English were translated into English using Google Translate. Four reviewers (XW, YL, MR, and AC) screened the English-language databases. Two reviewers (XW and YL) screened the Chinese-language databases. Disagreements were resolved by HN.

We included studies that were published between January 1, 1995, and December 31, 2018, and reported any of the following data in children younger than 5 years: community incidence rates of respiratory diseases (eg, influenza-like-illness, ALRI, and more severe infections) with laboratory-confirmed influenza virus; hospitalisation rates of ALRI, ALRI with hypoxaemia, or very severe ALRI with laboratory-confirmed influenza virus; in-hospital case-fatality ratios (hCFRs) of ALRI with laboratory-confirmed influenza virus.

Studies had to use a clearly defined case definition for specimen collection and testing, and studies that reported incidence and hospitalisation rate data had to show data for at least one complete calendar year (or at least one full influenza season if in a temperate region). We included hCFR data for any length of period.

We excluded studies: without a clear denominator population at risk (limited to those reporting incidence and hospitalisation rate data); those in which influenza was studied as a co-infection rather than the primary outcome (ie, only including cases tested negative for other pathogens, or only including cases co-infected with influenza virus and other pathogens); reporting modelled burden estimates; studies in which influenza virus infections were diagnosed based on serology alone (strict requirement on sampling timing and serial sampling; poor sensitivity); explicitly stating that they included any nosocomial infections in the numerator; only including population subgroups with high-risk conditions; or reporting data from the pandemic period (2009–10 season in temperate regions and the year of 2009 in tropical regions), or reporting combined data for the non-pandemic and pandemic periods.

### Identification and assembly of high-quality unpublished data

The Respiratory Virus Global Epidemiology Network is a collaboration network including more than 70 investigator groups working on respiratory viral infections (eg, respiratory syncytial virus and influenza virus) and provided additional unpublished data to supplement the systematic review.^10^ We formulated common case definitions and shared data through agreed common approaches to data analysis. Other investigators with relevant data were also invited to contribute data and participate in the analysis.

### Definitions

A study was defined as a dataset from one site in one published paper or from one research group in the collaboration network. Similar to our previous analysis, we used separate case definitions for community-based studies with active case ascertainment and hospital-based studies with passive case ascertainment (appendix p 2).^10^ For community-based studies, the 2005 WHO Integrated Management of Childhood Illnesses case definition^11^ for clinical pneumonia was used to define ALRI since these definitions were used in most included community studies and have been widely used as indications for the treatment of pneumonia. Two further severity levels for community-based studies included severe ALRI (ie, ALRI with chest wall indrawing) and very severe ALRI (ie, ALRI with any general danger signs).^11^ For hospital-based studies, where diagnoses were based on physicians' clinical judgment, we defined hospitalised ALRI as the physician-confirmed diagnosis of ALRI where hospitalisation was required or recommended. We defined two further severity levels: ALRI with hypoxaemia (SpO_2_ <90%), and very severe ALRI (ie, children hospitalised with ALRI with any general danger signs,^11^ admitted to intensive care units [ICUs], or requiring mechanical ventilation).

### Assessment of risk of biases

We adapted the Newcastle-Ottawa Scale to assess the risk of bias in included studies.^12^ The modified scale contains seven domains: study design, adjustment for health utilisation, patient groups excluded, case definition, sampling strategy (ie, how cases were selected for sample collection and testing), diagnostic testing, and hypoxaemia ascertainment (appendix pp 9–10). Critical assessments on the risk of bias (high or low) were made separately for each domain.

### Statistical analysis

We extracted the number of cases and scaled the population-at-risk for the proportion of eligible patients who were tested per study where available (appendix p 27). We did stratified analyses by case ascertainment, severity, developing status according to UNICEF definitions, World Bank income level (LICs, LMICs, upper-middle-income [UMICs], and high-income countries [HICs]), and three non-overlapping age bands (0–5 months, 6–11 months, and 12–59 months).^13^, ^14^ Due to the small number of studies in LICs (appendix pp 7–8), we combined LICs and LMICs (as LMICs) when reporting burden estimates. We imputed rates for missing 0–59 months using a multiple imputation approach to allow for the uncertainty in missing data, and imputed missing population-at-risks according to the population structure by country income group based on WHO life tables (appendix pp 28–29).^15^, ^16^, ^17^

**Figure 1:**
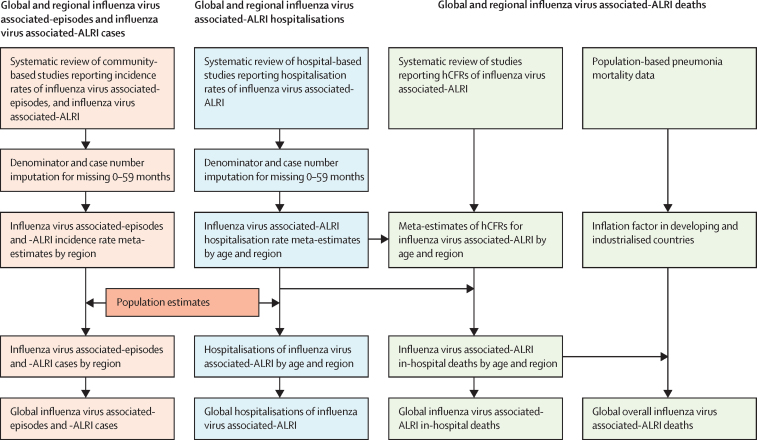
Approaches for estimation of global influenza virus morbidity and mortality in children under 5 years We report the number of influenza virus episodes and the number of cases of influenza-virus-associated ALRIs, global influenza-virus-associated-ALRI hospitalisations, and global estimates of influenza-virus-associated ALRI deaths in hospital and overall deaths (in community). This figure summarises our approach for each outcome and also shows how they relate to each other. Global estimates of hospitalisations for influenza virus ALRI were estimated by applying hospitalisation rates to population estimates (2018). Influenza-virus-associated ALRI in-hospital deaths were estimated by combining in-hospital case fatality ratios (hCFRs) for cases and hospitalisations. The inflation factor was estimated using three approaches (appendix pp 20–26), and we determined to use the most conservative estimate to calculate the number of overall deaths (appendix pp 20–26). A detailed description of imputation is in the appendix (p 28). ALRI=d acute lower respiratory infection.

Figure 1 summarises our approach for burden estimation. We estimated incidence rates, hospital admission rates, and hCFRs of influenza virus-associated respiratory infections using a generalised linear mixed model.^18^ In our previous analysis, we used a classic random-effect model. Compared with the previous model, the current model has an advantage in analysing studies with few events and does not require adding a continuity correction in case of zero events. The meta-estimates of influenza-virus-associated ALRI hospitalisation rate from the mixed regression model were similar to those from the classic random-effects model for most age groups, except for infants under 3 months (appendix pp 62–63). We estimated influenza-virus-associated ALRI cases (severe and very severe) and hospitalisations as described previously.^10^ Incidence rates and hospitalisation rates were applied to the UN population estimates for 2018.^19^, ^20^ The burden estimates and uncertainty ranges (URs) were calculated using Monte Carlo simulation, with the median value of 10 000 samples simulated from a log-normal distribution as the point estimate and the 2·5th and 97·5th percentiles as the 95% UR.^10^ We included all studies in the main analyses.

Previous evidence suggested that HIV infection^21^ was an important risk factor for influenza hospitalisation and death, and that the receipt of pneumococcal conjugate vaccine (PCV)^22^, ^23^, ^24^, ^25^ was associated with reduced risk of hospitalisation and death. As sensitivity analyses, we stratified data to assess how global influenza-virus-associated ALRI hospitalisations and in-hospital deaths were affected by (1) study bias (eg, a low risk of bias in sampling strategy), (2) low or high PCV coverage setting (≤60% or >60% in general population), and (3) low or high maternal HIV burden setting (according to the UNAIDS Global Plan; appendix pp 11–17).^26^, ^27^ We considered maternal HIV burden a good proxy of HIV burden in young children because mother-to-child transmission of HIV accounted for most HIV infection in young children.^28^ Results of sensitivity analyses are in the appendix (pp 11–19; 22–24). We also reported estimates for 2015 to enable comparisons with the 2015 estimates of respiratory syncytial virus ALRI burden (appendix, p 19).

We estimated in-hospital deaths by combining the influenza-virus-associated ALRI hospitalisations and hCFR meta-estimates as described previously.^10^ We included all studies in the main analysis of influenza-virus-associated ALRI in-hospital deaths, and conducted the same sensitivity analyses as described above for influenza-virus-associated ALRI hospitalisations (appendix pp 12–13, 16–17). For overall influenza-virus-associated ALRI mortality analysis, we invited investigators from the International Network for the Demographic Evaluation of Populations and their Health (INDEPTH) Network who had health and demographic surveillance data for defined populations to provide the numbers of pneumonia deaths occurring in and out of hospital.^29^ We identified six sites (in Mozambique, Kenya, South Africa, Burkina Faso, and Ghana) with eligible data (appendix p 20). For industrialised countries, the US Influenza-Associated Pediatric Mortality Surveillance System reports the most relevant data to our knowledge: location-specific deaths for children under 18 years with laboratory-confirmed influenza virus infection.^30^ For our analysis, we extracted the number of influenza-virus-associated paediatric deaths occurring in communities, emergency departments, and hospitals over 14 years during 2004–18 (excluding the pandemic year of 2009–10). Location-specific data on deaths among children aged 0–4 years were unavailable. We assumed health-care access in the USA was similar to other industrialised countries.

We estimated overall influenza-virus-associated ALRI deaths by combining in-hospital deaths and the median inflation factor of overall influenza-virus-associated ALRI deaths versus in-hospital deaths.^10^, ^31^ For industrialised countries we calculated the inflation factor directly by dividing the total number of paediatric influenza-virus-associated deaths by the number of deaths occurring in hospitals in the USA.^30^ For developing countries where the number of influenza-virus-specific deaths by location was unavailable, we divided the total number of pneumonia deaths by in-hospital pneumonia deaths, and used the median value across countries to represent the inflation factor for influenza-virus-specific ALRI deaths. Using this approach, we assumed that the relative contribution of influenza to ALRI deaths was the same for hospital and community settings. In sensitivity analyses we used two alternative approaches (approaches 2 and 3 in appendix pp 20–26). Approach 2 and 3 shared the same strategy in applying the proportion of influenza-virus-associated ALRI deaths among all-cause ALRI deaths to national all-cause pneumonia deaths, then dividing the national number of influenza-virus-associated ALRI in-hospital deaths, to back-calculate the inflation factor.

All analyses were done in R version 3.5.2, specifically the metafor package.^32^, ^33^ This study was done and reported in accordance with the Guidelines for Accurate and Transparent Health Estimates Reporting (GATHER) recommendations and the Strengthening the Reporting of Observational Studies in Epidemiology (STROBE) statement (appendix pp 72–74).^34^, ^35^

### Role of the funding source

The funders of the study had no role in study design, data collection, data analysis, data interpretation, or writing of the report. XW and HN (the corresponding author) had full access to all the data in the study and HN had final responsibility for the decision to submit for publication.

## Results

**Figure 2:**
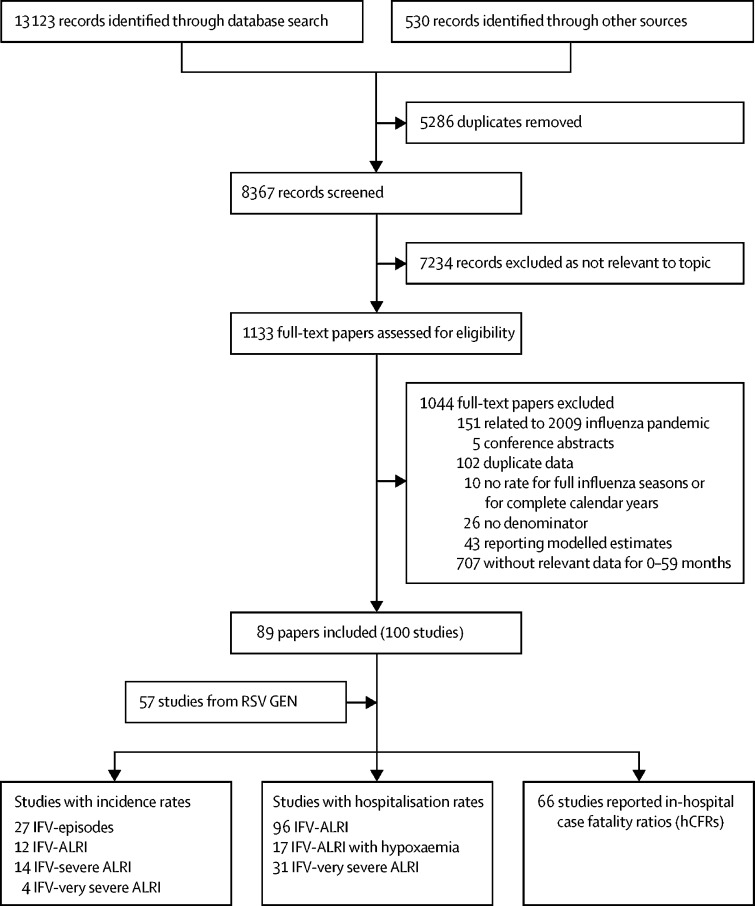
Flow diagram for selection of studies on seasonal influenza For multisite papers, the site-specific data were extracted where available and were analysed as one study; in this way 100 studies were extracted from 89 papers. One study could provide data on multiple outcomes among the same population; therefore the total number of studies was greater than the sum of studies by outcomes. RSV GEN=Respiratory Virus Global Epidemiology Network. IFV=influenza virus. ALRI=acute lower respiratory infection.

**Figure 3:**
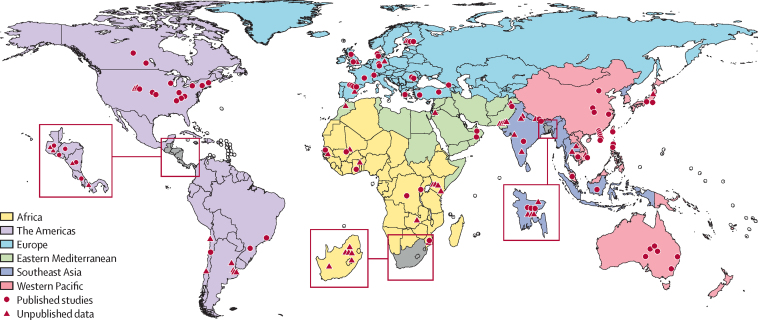
Location of included studies on influenza-virus-associated acute lower respiratory infection in children younger than 5 years

During 1995–2018, we identified 157 studies (123 new studies) providing data on community incidence, hospitalisation rates, and hCFRs (figure 2). Of these studies, 57 were unpublished and 100 were from published literature (89 papers). 106 studies (68%) were from developing countries; 14 studies (9%) were from LICs, 48 (31%) from LMICs, 29 (18%) from UMICs, 62 (39%) from HICs, and four studies from multiple countries from different country income groups (appendix pp 7–8; 30–58). Figure 3 shows where the included studies were conducted.

38 community-based studies reported incidence. 27 studies reported incidence of influenza virus episodes for 0–59 months, and 12 studies reported incidence for influenza-virus-associated ALRI cases (appendix pp 30–32). In 2018 among children under 5 years, we estimated 109·5 million (UR 63·1–190·6) influenza virus episodes and 10·1 million (UR 6·8–15·1) influenza-virus-associated ALRI cases globally. We were unable to estimate the burden in UMICs due to the scarcity of studies. We estimated 1·4 million (UR 0·6–3·4) severe influenza-virus-associated ALRI cases, and 0·4 million (UR 0·1–1·6) very severe influenza-virus-associated ALRI cases in children under 5 years in developing countries (table 1).

**Table 1:** Estimates of the incidence (per 1000 children per year), and number of influenza virus episodes and influenza virus-associated ALRI cases in children under 5 years in the community in 2018, by World Bank income level and development status

		**LMICs**	**UMICs**	**HICs**	**Developing countries**	**Industrialised countries**	Global burden estimates*
**Influenza virus episodes**							
Age 0–5 months							
	Studies	9	1	0	10	..	..
	Incidence (per 1000 children per year)†	80·2 (40·9–151·6)	..	..	80·8 (44·2–143·1)	..	..
	Episodes (1000s)	3552 (1848–6831)	..	..	5018 (2792–9023)	..	..
Age 6–11 months							
	Studies	2	1	0	2	..	..
	Incidence (per 1000 children per year)	165·9 (93·2–277·8)	..	..	164·8 (92·6–276·2)	..	..
	Episodes (1000s)	7233 (4201–12 457)	..	..	10 162 (5902–17 502)	..	..
Age 12–59 months							
	Studies	2	1	3	2	3	..
	Incidence (per 1000 children per year)	138·6 (57·2–299·4)	..	147·3 (82·6–248·9)	138·6 (57·2–299·4)	147·3 (82·6–248·9)	..
	Episodes (1000s)	47 524 (20 871–108 280)	..	7467 (4315–12 926)	67 125 (29 479–152 938)	8199 (4738–14 194)	74 448 (33 844–165 089)
Age 0–59 months							
	Studies	8 (3)‡	1	9 (3)	8 (3)	10 (3)	..
	Incidence (per 1000 children per year)	175·2 (101·5–302·3)	..	61·9 (32·5–117·9)	175·2 (101·5–302·3)	42·4 (17·2–104·8)	..
	Episodes (1000s)	75 538 (43 911–129 996)	..	3925 (2068–7453)	106 557 (61 943–183 376)	2953 (1202–7259)	109 510 (63 145–190 635)
**Influenza virus-associated ALRI cases**							
Age 0–5 months							
	Studies	4	1	0	5	..	..
	Incidence (per 1000 children per year)	4·9 (2·2–10·8)	..	..	8·5 (2·6–26·9)	..	..
	Episodes (1000s)	217 (98–479)	..	..	528 (165–1688)	..	..
Age 6–11 months							
	Studies	4	1	0	5	..	..
	Incidence (per 1000 children per year)	28 (23·6–33·3)	..	..	27·6 (23·4–32·6)	..	..
	Episodes (1000s)	1229 (1036–1459)	..	..	1702 (1444–2008)	..	..
Age 12–59 months							
	Studies	4	1	0	5	..	..
	Incidence (per 1000 children per year)	16·7 (8·6–32·3)	..	..	15·6 (8·9–27·1)	..	..
	Episodes (1000s)	5727 (29 66–11 062)	..	..	7556 (4344–13 149)	..	..
Age 0–59 months							
	Studies	7 (3)‡	1	4 (3)	8 (3)	4 (3)	..
	Incidence (per 1000 children per year)	14·6 (9·2–23·3)	..	9·3 (7·5–11·5)	15·6 (10·3–23·6)	9·3 (7·5–11·5)	..
	Episodes (1000s)	6299 (3961–10 021)	..	590 (477–729)	9498 (6305–14 313)	648 (524–800)	10 145 (6829–15 113)
**Severe influenza virus-associated ALRI cases**							
Age 0–5 months							
	Studies	11	1	0	12	0	..
	Incidence (per 1000 children per year)	4·1 (1·8–9·3)	..	..	5·1 (2·2–11·9)	..	..
	Episodes (1000s)	182 (80–411)	..	..	317 (137–733)	..	..
Age 6–11 months							
	Studies	3	1	0	4	0	..
	Incidence (per 1000 children per year)	3·9 (1·4–11·1)	..	..	6·1 (2·2–16·4)	..	..
	Episodes (1000s)	171 (61–479)	..	..	376 (139–1021)	..	..
Age 12–59 months							
	Studies	3	1	0	4	0	..
	Incidence (per 1000 children per year)	1·2 (0·6–2·4)	..	..	1·9 (0·7–5·1)	..	..
	Episodes (1000s)	412 (207–820)	..	..	920 (343–2471)	..	..
Age 0–59 months							
	Studies	6 (3)‡	1	0	7 (3)	0	..
	Incidence (per 1000 children per year)	1·7 (0·9–3·2)	..	..	2·4 (1–5·6)	..	..
	Episodes (1000s)	727 (386–1371)	..	..	1438 (612–3384)	..	..
**Very severe influenza virus-associated ALRI cases**							
Age 0–5 months							
	Studies	3	1	0	4	0	..
	Incidence (per 1000 children per year)	0·1 (0–122·1)	..	..	0·3 (0–13·3)	..	..
	Episodes (1000s)	4 (0–673)	..	..	19 (0–940)	..	..
Age 6–11 months							
	Studies	3	1	0	4	0	..
	Incidence (per 1000 children per year)	0·8 (0·2–4)	..	..	1 (0·3–3·5)	..	..
	Episodes (1000s)	35 (8–156)	..	..	62 (18–209)	..	..
Age 12–59 months							
	Studies	2	1	0	3	0	..
	Incidence (per 1000 children per year)	0·3 (0–2)	..	..	0·5 (0·1–2·3)	..	..
	Episodes (1000s)	103 (5–2024)	..	..	242 (51–1152)	..	..
Age 0–59 months							
	Studies	3 (1)‡	1	0	4 (1)	0	..
	Incidence (per 1000 children per year)	0·5 (0·1–2·8)	..	..	0·7 (0·2–2·7)	..	..
	Episodes (1000s)	211 (37–1194)	..	..	416 (105–1641)	..	..

Data in parentheses are estimated uncertainty ranges. Data were imputed using a multiple imputation approach. ALRI=acute lower respiratory infection. LMICs=lower-middle income countries. UMICs=upper-middle income countries. HICs=high-income countries. ..=not available.

* Global burden estimates were developed by summing up estimates for 0–59 months by country development status classified according to UNICEF definition.

† Incidence was estimated using generalised linear mixed models.

‡ Data in parentheses are the number of imputed studies.

We identified 96 studies reporting influenza-virus-associated ALRI hospital admission rates, including 59 studies with data on age groups 0–5 months, 6–11 months, or 12–59 months (appendix pp 37–43). Among children under 5 years, we estimated 870 000 hospital admissions (UR 543 000–1 435 000) for influenza-virus-associated ALRI globally, of which 23% and 21% were in infants aged 0–5 months and 6–11 months, respectively (table 2). An estimated 420 000 hospital admissions for influenza-virus-associated ALRI (UR 261 000–677 000) occurred in LMICs. In subgroup analyses of studies with low risk of bias and in analyses stratified by low or high national PCV coverage and by low or high national maternal HIV burden, the hospital admission estimates were similar to those in the main analysis; the point values ranged from 772 000 to 895 000 for hospital admissions, with wide and overlapping URs (appendix pp 11–15).

**Table 2:** Estimates of hospital admission rates (per 1000 children per year), and number of hospital admissions in children under 5 years with influenza virus-associated ALRI in 2018, by World Bank income level and development status

		**LMICs**	**UMICs**	**HICs**	**Developing countries**	**Industrialised countries**	Global*
**Influenza virus–associated ALRI**							
Age 0–5 months							
	Studies	13	9	13	24	11	..
	Rate (per 1000 children per year)†	1·8 (1·1–3·1)	3·7 (1·8–7·4)	4·4 (3·1–6·3)	2·8 (1·8–4·3)	3·7 (2·7–5·3)	..
	Hospital admissions (1000s)	80 (48–133)	68 (34–138)	28 (20–40)	174 (113–268)	26 (18–36)	200 (131–304)
Age 6–11 months							
	Studies	11	8	9	21	7	..
	Rate (per 1000 children per year)	1·5 (1–2·4)	3·8 (1·7–8·3)	3·5 (1·4–8·8)	2·7 (1·7–4·3)	2·6 (0·9–7·6)	..
	Hospital admissions (1000s)	66 (43–102)	70 (32–154)	22 (9–55)	166 (105–264)	18 (6–52)	185 (111–317)
Age 12–59 months							
	Studies	17	11	20	35	13	..
	Rate (per 1000 children per year)	0·8 (0·5–1·3)	0·8 (0·3–2)	1·2 (0·6–2·2)	0·9 (0·6–1·5)	0·9 (0·4–1·8)	..
	Hospital admissions (1000s)	274 (171–441)	117 (46–301)	61 (32–116)	436 (276–688)	50 (24–106)	486 (300–794)
Age 0–59 months							
	Hospital admissions (1000s)	420 (261–677)	255 (111–593)	111 (60–211)	776 (494–1220)	94 (48–194)	870 (543–1415)
**Influenza virus-associated ALRI with hypoxaemia**							
Age 0–5 months							
	Studies	8	2	..	10	..	..
	Rate (per 1000 children per year)	0·9 (0·5–1·6)	0·9 (0·5–1·5)	..	0·9 (0·6–1·4)	..	..
	Hospital admissions (1000s)	40 (22–71)	17 (10–29)	..	56 (37–85)	..	..
Age 6–11 months							
	Studies	8	2	..	10	..	..
	Rate (per 1000 children per year)	0·7 (0·3–1·4)	1 (0·7–1·6)	..	0·8 (0·5–1·1)	..	..
	Hospital admissions (1000s)	31 (14–66)	18 (12–28)	..	49 (33–73)	..	..
Age 12–59 months							
	Studies	8	1	2	9	2	..
	Rate (per 1000 children per year)	0·2 (0·1–0·4)	..	0 (0–1·3)	0·2 (0·1–0·3)	0 (0–1·3)	..
	Hospital admissions (1000s)	69 (34–137)	..	0 (0–4)	97 (56–167)	0 (0–4)	97 (56–172)
Age 0–59 months							
	Hospital admissions (1000s)	139 (71–274)	..	..	202 (126–326)	7 (1–62)‡	209 (127–387)
**Very severe influenza virus-associated ALRI**							
Age 0–5 months							
	Studies	9	3	2	12	2	..
	Rate (per 1000 children per year)	0·6 (0·2–1·4)	0·2 (0·1–0·4)	0·3 (0·1–0·7)	0·3 (0·1–0·9)	0·3 (0·1–0·7)	..
	Hospital admissions (1000s)	27 (10–70)	4 (2–7)	2 (1–5)	19 (6–56)	2 (1–6)	21 (7–61)
Age 6–11 months							
	Studies	9	3	2	12	2	..
	Rate (per 1000 children per year)	0·5 (0·3–0·9)	0·3 (0·1–1)	0·1 (0–0·6)	0·4 (0·2–0·8)	0·1 (0–0·6)	..
	Hospital admissions (1000s)	22 (13–38)	6 (2–17)	1 (0–7)	25 (12–49)	1 (0–8)	25 (12–57)
Age 12–59 months							
	Studies	9	5	8	16	6	..
	Rate (per 1000 children per year)	0·1 (0–0·3)	0·1 (0–0·2)	<0·05	0·1 (0–0·2)	<0·05	..
	Hospital admissions (1000s)	34 (4–263)	15 (2–92)	0 (0–0)	48 (8–303)	0 (0–0)	49 (8–304)
Age 0–59 months							
	Hospital admissions (1000s)	83 (27–371)	24 (6–116)	3 (1–12)	92 (26–408)	3 (1–13)	95 (28–421)

Data in parentheses are estimated uncertainty ranges. ALRI=acute lower respiratory infection. LMICs=lower-middle income countries. UMICs=upper-middle income countries. HICs=high-income countries. ..=not available.

* Global burden estimates were developed by summing up estimates in three non-overlapping age groups (0–5 months, 6–11 months, and 12–59 months), and in developing and industrialised countries classified according to UNICEF definition.

† Hospital admissions rates were estimated using generalised linear mixed models.

‡ In industrialised countries, two studies reported the hospital admission rates for influenza virus-associated ALRI with hypoxaemia for 0–59 months; the meta-estimate was 0·1 (<0·05–0·4), translating to 7000 (UR 1000–62 000) hospital admissions.

For infants under 1 year, incidence meta-estimates of influenza virus episodes and of influenza-virus-associated ALRI cases increased with age (p for linear trend <0·05, appendix p 67). The linear trend for hospital admission rates was not significant. However, the hospital admission rate of influenza-virus-associated ALRI was lower in neonates (0·9 per 1000 neonates per year, 95% CI 0·4–1·8), and was consistently higher in infants older than 1 month (1·6–1·9 per 1000 infants per year) in developing countries. Only two studies reported hospital admission rates of influenza-virus-associated ALRI by narrow age groups among infants in industrialised countries.

Based on 15 studies, we estimated 209 000 hospital admissions for influenza-virus-associated ALRI with hypoxaemia (UR 127 000–387 000) globally in children under 5 years. An estimated 139 000 (UR 71 000–274 000) hospital admissions occurred in LMICs (table 2). For very severe influenza-virus-associated ALRI, based on 24 studies, we estimated 95 000 (UR 28 000–421 000) hospital admissions in children under 5 years globally, 22% and 26% in infants aged 0–5 months and 6–11 months, respectively (table 2). An estimated 83 000 (UR 27 000–371 000) hospital admissions for very severe influenza-virus-associated ALRI occurred in LMICs.

66 studies reported hCFRs for influenza-virus-associated ALRI in children under 5 years, including 28 studies with data on three non-overlapping age bands (appendix pp 49–53). We estimated that hCFRs for influenza-virus-associated ALRI were 3·1% (95% CI 1·3–6·9), 2·0% (0·6–6·2), and 1·4% (0·7–2·8) in children aged 0–5 months, 6–11 months, and 12–59 months in developing countries, respectively (table 3). By country-income group, hCFRs were higher in LMICs (3·4%, 95% CI 1·4–7·9, for 0–59 months) than UMICs (1·4%, 0·6–3·5) and HICs (0·5%, 0·2–1·4). The analysis stratified by age and country development status yielded 15 300 influenza-virus-associated ALRI (UR 5800–43 800) in-hospital deaths in children under 5 years, with 36% and 23% among infants aged 0–5 months and 6–11 months, respectively. About 82% of influenza-virus-associated ALRI in-hospital deaths occurred in LMICs. We did stratified analyses in studies with low risk of bias, by low or high national PCV coverage and by low or high maternal HIV burden. In these sensitivity analyses, in-hospital mortality estimates were similar to those in the main analysis; the point values ranged from 13 800 to 20 800 for in-hospital mortality, with wide and overlapping URs (appendix pp 12–13, 16–17).

**Table 3:** In-hospital case fatality ratio (hCFR) meta-estimates and in-hospital deaths in children under 5 years with influenza virus-associated ALRI in 2018, by World Bank income level and development status

			**LMICs**	**UMICs**	**HICs**	**Developing countries**	**Industrialised countries**	Global*
Studies†			10	11	7	23	5	..
	Age 0–5 months							
		hCFR (%)‡	3·2 (0·6–15·4)	2·6 (0·9–7·5)	0·5 (0–4·6)	3·1 (1·3–6·9)	0·3 (0–6·9)	..
		Deaths	2500 (500–13 800)	1800 (500–6200)	100 (0–4100)	5400 (2100–13 700)	100 (0–2700)	5500 (2100–16 300)
	Age 6–11 months							
		hCFR (%)	8·1 (4·1–15·3)	0·7 (0·1–7·4)	0·8 (0·2–3·2)	2·0 (0·6–6·2)	0·9 (0·2–3·4)	..
		Deaths	5300 (2400–11 600)	500 (0–4700)	200 (0–900)	3300 (900–11 500)	200 (0–900)	3500 (1000–12 400)
	Age 12–59 months							
		hCFR (%)	3·3 (1·7–6·3)	0·8 (0·3–2·2)	0·4 (0·1–2·1)	1·4 (0·7–2·8)	0·4 (0·1–2·7)	..
		Deaths	9100 (4000–20 100)	900 (200–3700)	200 (0–1300)	6100 (2700–13 800)	200 (0–1200)	6300 (2700–14 900)
	Age 0–59 months							
		Deaths	17 000 (6900–45 100)	3200 (800–14 400)	600 (100–6200)	14 800 (5700–39 000)	500 (100–4800)	15 300 (5800–43 800)

Data in parentheses are estimated uncertainty ranges. ALRI=acute lower respiratory infection. LMICs=lower-middle income countries. UMICs=upper-middle income countries. HICs=high-income countries. ..=not available.

* Global estimates were calculated by summing up estimates in three non-overlapping age groups (0–5 months, 6–11 months, and 12–59 months), and in developing and industrialised countries according to UNICEF definitions.

† hCFR meta-estimates were based on studies providing data for the three non-overlapping age bands.

‡ hCFRs were estimated using generalised linear mixed models.

For industrialised countries, we estimated an inflation factor of 1·6 (range 1·3–1·9 across 14 years) based on the data for US paediatric influenza-virus-associated deaths. For developing countries, we used an inflation factor of 2·3 in the main analysis (range 1·5–3·5 across six sites), and estimated an overall influenza-virus-associated ALRI mortality of 34 800 (UR 13 200–97 200) in children under 5 years globally. In sensitivity analyses, we estimated a higher inflation factor in approach 2 (3·0) and approach 3 (3·4). Using the two approaches, the point overall mortality estimate could increase by 30–47%, with wide and overlapping URs (appendix pp 20–26).

## Discussion

We estimated that globally in 2018, in children under 5 years, there were 109·5 million influenza virus episodes, around 10 million cases of influenza-virus-associated ALRI, about 870 000 hospital admissions for influenza-virus-associated ALRI, 15 000 in-hospital deaths from influenza-virus-associated ALRI, and around 35 000 total deaths from influenza-virus-associated ALRI. The wide uncertainty ranges of the burden estimates reflected differences across studies, arising from the variation in influenza activity between seasons, variation in influenza epidemiology between populations and methodological differences, and lack of data, especially mortality data.

The incidence rates of influenza virus episodes and influenza-virus-associated severe ALRI in children under 5 years were consistent with our previous estimates for 2008.^5^ However, we estimated a lower incidence of influenza-virus-associated ALRI compared with the previous review for 0–59 months in developing countries. In the previous review, the incidence of influenza-virus-associated ALRI was only based on three studies from two sites. Our updated estimate was refined by incorporating new data with larger sample sizes achieved by multiple-season observation and data from more geographically diverse areas (12 studies). Improved influenza vaccine uptake is not likely to be the reason for the lower incidence of influenza-virus-associated ALRI since paediatric influenza immunisation policy had not been introduced (in Bangladesh, India, and Pakistan), or was only introduced in children with chronic diseases (in Nicaragua), or achieved low coverage (in South Africa) during the study periods.^36^ The national PCV coverage was above 60% midway in three studies in Nicaragua, Pakistan, and South Africa; increase in PCV uptake might contribute to the lower point value of incidence rates.^26^, ^37^ The reduction in influenza-virus-associated ALRI incidence rates is consistent with a general decrease in the incidence of all-cause ALRI.^2^ Our estimate of influenza-virus-associated ALRI cases, after accounting for the attributable fraction of influenza virus in influenza-virus-associated ALRI (80%), generally agreed with the number of influenza-virus-attributable ALRI cases (about 8 million) estimated by the Institute for Health Metrics and Evaluation (IHME).

We estimated about 870 000 influenza-virus-associated ALRI hospitalisations (about 5% of all-cause hospitalised ALRI), similar to our previous estimate.^5^, ^38^ Our estimate developed using an incidence-based approach, is lower than the IHME estimate of influenza-virus-attributable ALRI hospitalisations for 2017 (about 2 200 000) derived from a proportion-based approach.^39^ The difference in the two estimates might reflect the difference in analytical methods; data used in the two analytical methods were not directly comparable. Nevertheless, a previous study reported an estimate similar to our result, although using a proportion-based approach.^4^ Our global estimates for influenza-virus-associated ALRI hospitalisations changed marginally (if at all) in sensitivity analyses, suggesting that they were not affected by study biases, or inclusion of data from different settings—eg, with different PCV coverages and HIV burden. The primary objective of doing these sensitivity analyses was to assess to what extent the global estimates would be affected by these factors. It is important to note that the estimates in the stratified analyses by PCV coverage (and by 2009) were not directly comparable because most data were from different areas. We identified five studies reporting influenza-virus-associated ALRI hospitalisation rates for at least 5 consecutive years with PCV introduced midway. In a pooled regression analysis of the five studies, we did not find a significant difference in influenza-virus-associated ALRI hospitalisation rates between high PCV coverage periods and low coverage periods. However, this finding needs to be interpreted with caution because the five studies had short observation periods (5–8 years). In the main analyses of influenza-virus-associated ALRI hospitalisations, most studies were from the post-2009 period, and hospitalisation estimates did not change after excluding pre-2009 data. Moreover, the estimate did not change after excluding 2010 data, when the influenza pandemic affected some regions.^40^ 80% of influenza-virus-associated ALRI can be attributed to influenza based on a systematic review of case-control studies investigating the viral profile in children under 5 years with and without ALRI. Thus, based on our estimate, 696 000 ALRI hospitalisations could be attributed to influenza virus in children under 5 years.^41^

We estimated 15 300 influenza-virus-associated ALRI in-hospital deaths based on 28 hCFR studies. The estimates were not affected by risk of bias or by the inclusion of data from different PCV coverage settings and HIV burden settings. We estimated up to 4% of all childhood ALRI deaths were associated with influenza virus.^2^ Our estimate was between the IHME influenza-virus-attributable ALRI mortality estimate (about 23 400 influenza-virus-attributable ALRI deaths in 2017) and the influenza-virus-associated respiratory mortality estimate in a study by Iuliano and colleagues (44 888, 95% credible interval 9243–105 690, in 2015) for 92 countries where 92% of respiratory deaths occurred.^39^, ^42^ The IHME mortality estimate was modelled on all-cause ALRI deaths, the percentage of influenza virus in patients with ALRI from published literature, the attributable fraction of influenza virus for influenza-virus-confirmed ALRI, and the ratio of case-fatality between viral ALRI and bacterial ALRI (viral-to-bacterial case fatality ratio).^39^ Iuliano and colleagues did a time series analysis using records for vital respiratory deaths and influenza circulation data in three countries, and extrapolated these to other countries. The different estimates in the two studies reflected the difference in case definitions (respiratory deaths *vs* ALRI deaths) and in statistical models;^43^ we used three different approaches to model the number of overall deaths, and reported the most conservative influenza-virus-associated ALRI mortality burden.

For calculation of the upper bound of influenza-virus-associated ALRI mortality (overall influenza-virus-associated ALRI mortality), we estimated a more conservative inflation factor (2·3) for developing countries in the main analysis than those used in another two approaches in sensitivity analyses (3·0 in approach 2 and 3·4 in approach 3). Each approach has potential biases. One modelling study in South Africa estimated that a significantly higher percentage of influenza-virus-attributable deaths occurred outside hospitals than the percentage of all-cause deaths occurring outside hospitals (57% versus 41%).^44^ This finding suggests that using the inflation factor for all-cause pneumonia might underestimate the influenza-virus-specific estimate in the main analysis. Potential biases for approaches 2 and 3 are explained in the appendix, pp 20–24. Although estimated using data from six sites, the estimated inflation factor was still limited by lack of data. Health-care access varies significantly across the world; most data we included were from rural areas and from LMICs. The inflation factor and the upper bound of mortality estimate in developing countries might be overestimated due to the poor access to health care at these sites. Care-seeking has been investigated in national household surveys of 11 Middle East and North African countries: 34–80% (median 70% approximately) of children under 5 years with acute respiratory infections (caregiver's self-report) sought care in health facilities.^45^ However, it is still challenging to understand the scenario of ALRI deaths from these data. For industrialised countries, we estimated the inflation factor using mortality data for 0–17 years, which might cause an underestimation if young children are more likely to die before being admitted, or an overestimation if young children are more likely to be taken to health-care facilities when they are sick.^46^

We estimated the highest hCFRs in LMICs, and 82% of the in-hospital deaths occurred in these countries. However, only 10% of LICs and LMICs have had a national policy for influenza immunisation in children (13% for pregnant women) in 2014.^9^ Where present there were mostly very low or unknown levels of vaccine coverage.^6^, ^47^, ^48^, ^49^, ^50^ According to previous evidence, a high influenza vaccine coverage (about 70%) can substantially reduce influenza-associated hospitalisations and deaths in children under 5 years of age.^51^

A large proportion of influenza-virus-associated ALRI hospitalisations (44%) and in-hospital deaths (59%) occurred during infancy, with 23% of hospitalisations and 36% of the in-hospital deaths in infants under 6 months. Our estimates of influenza-virus-associated ALRI hospitalisations are likely to underestimate the effect of influenza. Influenza can cause primary infections or expose children to severe secondary bacterial infections while not being detected at specimen collection. Maternal influenza vaccination trials assessing vaccine efficiency against hospitalised ALRI might help to understand the direct and indirect effects of influenza in causing ALRI.^52^, ^53^ The infant data we included were from developing countries with either no influenza immunisation policy or low influenza vaccine coverage in pregnant women during the study periods (ie, Bangladesh, Guatemala, India, Kenya, Mozambique, South Africa, and Thailand).^36^, ^54^, ^55^ Findings from two trials in Mali and South Africa suggested that maternal influenza immunisation is an effective intervention against influenza infections in the first 3 months of age.^56^, ^57^ Influenza virus predisposes individuals to secondary bacterial infection and adverse outcomes associated with such infections.^58^ Therefore, preventing influenza virus through maternal immunisation could play a role in reducing the burden of all-cause ALRI. For example, in a pooled analysis of three maternal influenza immunisation trials in South Africa, Mali, and Nepal, there was a 20% reduction in all-cause severe clinical pneumonia in infants under 6 months.^53^ However, there was heterogeneity in this effect between three trials; therefore, it would be challenging to quantify the global effect of maternal influenza immunisation on reducing all-cause ALRI in early infancy.

Our burden estimates were generally robust to the sensitivity analyses we performed. However, there were some limitations in our review. First, although we prespecified case definitions, heterogeneity between studies still existed; for example, 50% of hospital-based studies used other definitions (eg, hospitalised acute respiratory infection). This might cause an overestimation of influenza-virus-associated ALRI hospitalisations. Second, PCR was used in about 70% of hospital-based studies to detect influenza, and diagnostic tools with lower sensitivity were used in 30% of studies. However, the hospitalisation estimates did not change when restricting the analysis to these PCR studies. Third, not all infections were tested for influenza virus. For incidence and hospitalisation rates, we have adjusted for underdetection, assuming the proportion of influenza virus in tested and untested cases was the same. Studies were systematically assessed, and the rates might have been biased in some studies: for influenza-virus-associated ALRI hospitalisation rates, at least 90% of eligible cases were tested in 50% of studies; less than 90% of cases were tested in 30% of studies, and the main reasons for not collecting specimens included cases being tested systematically (17%), refusal and discharge (10%), and unknown reasons (3%); in the remaining 20% of studies the proportion tested was unavailable. We reported unadjusted hCFRs, which might be underestimated because children who were very ill were less likely to be sampled and tested, as suggested by the higher hCFRs among untested cases than those tested (appendix p 26). Fourth, hospitalisation estimates were affected by access to health care and health-seeking behaviour, so the potential numbers of influenza-virus-associated ALRI hospitalisations are likely to be higher in resource-limited areas and in older children who are less likely to be taken to health-care facilities when they are sick.^46^ Furthermore, influenza vaccine coverage has increased over the last decade in industrialised countries (eg, in the USA). Estimates based on data during the low vaccine coverage period could cause an overestimation. Due to the scarcity of influenza vaccine coverage data in most countries, we determined not to adjust influenza vaccine use in estimating influenza-associated burden.

Based on 157 studies of which 123 studies were not included in our 2008 estimates, we reported the new estimates in the era with the circulation of influenza A/H1N1pdm09. Although we included more studies than our previous review, estimates were still based on limited data. High-quality in-hospital mortality data, especially from LICs, are needed to improve the global in-hospital mortality estimate. Studies reporting age-stratified influenza-virus-associated ALRI morbidity and mortality burden should be encouraged. Moreover, further influenza-virus-associated ALRI mortality studies should ensure optimal sample size for robust mortality estimation. These efforts will help refine estimates of global burden of influenza in young children and understand the role of influenza in childhood disease, and inform policy. The initiation of post-mortem surveillance such as in the Child Health and Mortality Prevention Surveillance Network, will provide evidence to improve influenza-virus-associated mortality estimation.^59^
