# Supplementary material for: Global burden of respiratory infections associated with seasonal influenza in children under 5 years in 2018: a systematic review and modelling study
Source: Lancet Glob Health. 2020 Feb 20;8(4):e497–510. doi: 10.1016/S2214-109X(19)30545-5 (PMC7083228; doi:10.1016/S2214-109X(19)30545-5)
Supplement: Supplementary appendix [file mmc1.pdf]

THE LANCET
Global Health

Supplementary appendix

This appendix formed part of the original submission and has been peer reviewed. We post it as supplied by the authors.

Supplement to: Wang X, Li Y, O'Brien KL, et al. Global burden of respiratory infections associated with seasonal influenza in children under 5 years in 2018: a systematic review and modelling study. *Lancet Glob Health* 2020; published online Feb 20. http://dx.doi.org/10.1016/S2214-109X(19)30545-5.

Supplementary material for "Global burden of respiratory infections associated with seasonal influenza in young children in 2018: a systematic review and modeling study"

Case definitions and glossary

Community-based studies: studies where eligible cases are actively identified through regular visits to households. We considered studies conducted in outpatient departments or in the offices of general practitioners in industrialised countries as good proxies of community-based studies.
Hospital-based studies: studies where children are enrolled when they are admitted into hospital.

Case definition for community-based studies

1. IFV-episodes: the entire spectrum of respiratory infections from influenza-like illness, ALRI, and severe ALRI AND laboratory confirmed IFV. Influenza-like illness was defined as sudden fever (>38 deg C) and cough or sore throat and absence of other diagnosis.
2. IFV-associated ALRI: cough or difficulty breathing with increased respiratory rate for age (cut-offs same as in WHO Integrated Management of Childhood Illnesses [IMCI] case definition) AND laboratory confirmed IFV.
3. IFV-associated severe ALRI: for children aged 2-59 months, cough or difficulty in breathing with chest wall indrawing AND laboratory confirmed IFV; for children aged <2 months, increased respiratory rate [RR] (>60 breaths/ min) OR chest wall indrawing AND laboratory confirmed IFV.
4. IFV-associated very severe ALRI: cough or difficulty in breathing with any danger signs (cyanosis, difficulty in breastfeeding or drinking, vomiting everything, convulsions, lethargy, or unconsciousness, head nodding) AND laboratory confirmed IFV.

Case definition for hospital-based studies

1. Hospitalised IFV-associated ALRI: all children with physician confirmed diagnosis of ALRI (pneumonia or bronchiolitis) that are hospitalised or recommended hospitalisation AND laboratory confirmed IFV.
2. Hospitalised IFV-associated ALRI with hypoxaemia: hospitalised ALRI cases with hypoxaemia (as defined below) AND laboratory confirmed IFV.
3. Hospitalised IFV-associated very severe ALRI: hospitalised ALRI with any danger signs (cyanosis, difficulty in breastfeeding or drinking, vomiting everything, convulsions, lethargy, or unconsciousness, head nodding) OR proxies for very severe disease – requiring mechanical ventilation OR ICU admission.
4. Hypoxaemia: SpO_2 <90% (at altitude <=2500 m above sea level) in children aged 1-59 months and <88% for neonates; SpO_2 <87% (at altitude >2500 m above sea level) in children aged 1-59 months and <85% for neonates.

The relationship between the case definitions of different severity is displayed in Supplementary figure 1.

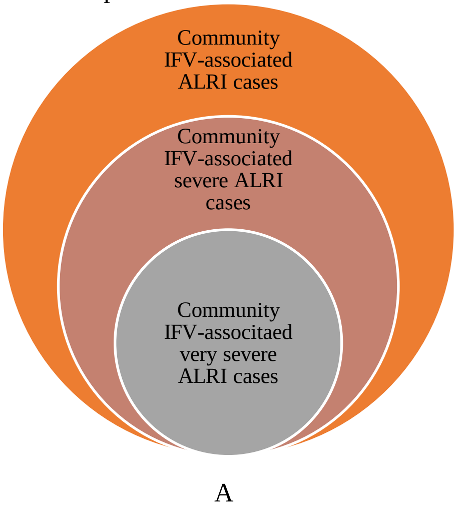

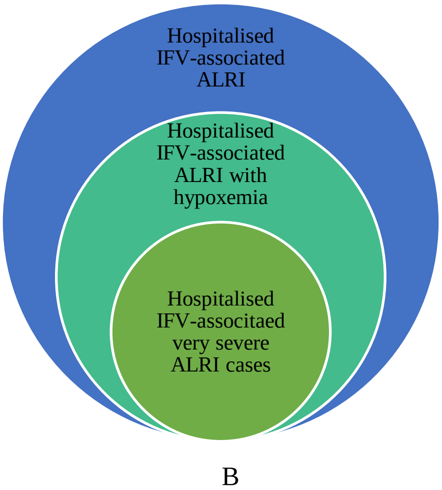

Supplementary figure 1: The relationship between community IFV-associated ALRI, IFV-associated severe ALRI, and IFV-associated very severe ALRI (A). The relationship between hospitalised IFV-associated IFV-ALRI, IFV-associated ALRI with hypoxemia, and IFV-associated very severe ALRI (B). The size of each circle is not proportionate to the number of cases for each severity.

Glossary of terms and abbreviations.

IFV	Influenza virus
ALRI	Acute lower respiratory infection
ILI	Influenza-like-illness, defined as sudden fever (>38 deg C) and cough or sore throat and absence of other diagnosis
IFV-episodes (ALRI, severe ALRI, very severe ALRI)	Influenza-associated respiratory infections (specific definitions see above)
SARI	Severe acute respiratory infection
LICs, LMICs, UMICs, HICs	By World Bank Classification: low-income countries, lower middle-income countries, upper middle-income countries, high-income countries
hCFR	In-hospital case-fatality ratio
UR	Uncertainty range
RSV	Respiratory syncytial virus
SpO2	Peripheral oxygen saturation
ICU	Intensive care unit
MV	Mechanical ventilation
IMCI	Integrated Management of Childhood Illnesses
UNICEF	United Nations International Children's Emergency Fund
WHO	World Health Organisation
UN	United Nations
HIV	Human immunodeficiency virus
PCV	Pneumococcal conjugate vaccine
INDEPTH	The International Network for the Demographic Evaluation of Populations and their Health
US	The United States of America
AFE	The attributable fraction among the exposed. Here we meant the attributable fraction for IFV-associated ALRI
GATHER	the Guidelines for Accurate and Transparent Health Estimates Reporting
IHME	the Institute for Health Metrics and Evaluation

Search Strategy

Medline (Ovid)
1. exp Influenza, Human/
2. exp Influenzavirus B/ or exp Influenzavirus A/ or exp Influenzavirus C/
3. *Influenza Vaccines/ or *Influenza A virus/ or *Influenza, Human/ or *Respiratory Tract Infections/ or *Disease Outbreaks/ or *Influenza A Virus, H1N1 Subtype/
4. H1N1pdm.mp
5. pH1N1.mp
6. 2009H1N1.mp
7. exp Bronchiolitis/ or exp Bronchiolitis, Viral/
8. exp Respiratory Tract Diseases/
9. exp Respiratory Tract Infections/ or acute respiratory infections.mp. or Influenza, Human/
10. exp Pneumonia, Viral/ or *Pneumonia/ or acute lower respiratory infections.mp.
11. exp Incidence/
12. exp Prevalence/
13. exp Morbidity/
14. exp Child Mortality/ or exp Infant Mortality/ or *Hospital Mortality/ or exp Mortality/
15. exp Death/ or exp "Cause of Death"/
16. pediatric mortality.mp
17. paediatric mortality.mp
18. pediatric death.mp.
19. paediatric death.mp.
20. burden.mp.
21. (1 or 2 or 3 or 4 or 5 or 6) and (7 or 8 or 9 or 10) and (11 or 12 or 13 or 14 or 15 or 16 or 17 or 18 or 19 or 20)
22. animals
23. 21 not 22
24. Limit 23 to (yr="2009-2018" and ("all infant (birth to 23 months)" or "preschool child (2 to 5 years)"))

Embase (Ovid)
1. exp Influenza virus A/ or exp influenza/ or exp Influenza virus A H3N2/ or exp Influenza virus/ or exp Influenza virus A H1N1/ or exp Influenza virus B/
2. exp pandemic influenza/ or exp pandemic
3. influenza outbreak*.mp
4. exp 2009 H1N1 influenza/ or 2009 H1N1.mp
5. H1N1pdm.mp
6. pH1N1.mp
7. exp respiratory tract infection/
8. exp lower respiratory tract infection/
9. exp virus pneumonia/ or exp pneumonia/
10. exp bronchiolitis/ or exp viral bronchiolitis/
11. exp incidence/
12. exp prevalence/
13. exp morbidity/
14. exp mortality/ or exp childhood mortality/ or exp infant mortality/
15. exp death/ or exp child death/
16. paediatric mortality.mp.
17. paediatric mortality.mp.
18. paediatric death.mp.
19. paediatric death.mp.
20. burden.mp.
21. (1 or 2 or 3 or 4 or 5 or 6) and (7 or 8 or 9 or 10) and (11 or 12 or 13 or 14 or 15 or 16 or 17 or 18 or 19 or 20)
22. animals
23. 21 not 22
24. Limit 23 to (yr="2009 -2018" and (infant or preschool child <1 to 6 years>))

Global Health (Ovid)

1. exp influenza A/ or exp Influenza A virus/ or exp Influenza B virus/ or exp influenza viruses/ or exp swine influenza A viruses/ or exp swine influenza viruses/ or exp influenza B/ or exp influenza/
2. pandemic influenza.mp
3. 2009 H1N1.mp
4. H1N1pdm.mp
5. pH1N1.mp
6. influenza outbreak*.mp
7. (respiratory diseases or lower respiratory tract infections).sh.
8. exp pneumonia/
9. bronchiolitis.mp.
10. exp incidence/
11. burden.mp.
12. exp morbidity/
13. exp infant mortality/ or exp mortality/
14. exp death/ or exp "causes of death"/
15. paediatric mortality.mp.
16. paediatric mortality.mp.
17. paediatric death.mp.
18. paediatric death.mp.
19. (1 or 2 or 3 or 4 or 5 or 6) and (7 or 8 or 9) and (10 or 11 or 12 or 13 or 14 or 15 or 16 or 17 or 18)
20. Limit 19 to yr="2009-2018"

CINAHL
1. (MH “Influenza, Humans+”) OR (MH “Influenzavirus B+”) OR (MH “Influenzavirus A+”) OR (MM “Influenza, Pamdemic (h1N1)2009”))
2. (MM “Bronchiolitis”) OR (MM “Community-Acquired Pneumonia”) OR (MM “Pneumonia, Viral”) OR (MM “Influenza, Human”)
3. “children”
4. 1 AND 2 AND 3
Limiters: 2009-2018

Web of Science
TOPIC: influenza or 2009 H1N1 or H1N1pdm or pH1N1 AND TOPIC: children AND TOPIC: acute respiratory infection or pneumonia
Time span= 2009-2018

Global Index Medicus [including WHOLIS (KMS)]
tw:((tw:(influenza) OR tw:(ph1n1) OR tw:(h1n1pdm) OR tw:(2009 h1n1)) AND (tw:(children))) AND (db:("WPRIM" OR "LILACS" OR "IMSEAR" OR "IMEMR" OR "WHOLIS") AND year_cluster:("2009" OR "2010" OR "2011" OR "2012" OR "2013" OR "2014" OR "2015" OR "2016" OR "2017" OR"2018"))

Google
children acute respiratory infections influenza
H1N1pdm OR pH1N1 OR burden OR incidence OR mortality OR morbidity OR death
-animal
filetype:pdf

CNKI (China Knowledge Resource Integrated Database)
Topic: respiratory infection or respiratory tract infection or pneumonia or bronchiolitis
AND Topic: influenza virus or H1N1 or pandemic influenza
AND Topic: morbidity or mortality or disease burden or hospitalization
AND Topic: children or infant
From 1995 to Dec 31 2018.

Wanfang Data
Topic: respiratory infection or respiratory tract infection or pneumonia or bronchiolitis
AND topic: influenza virus or H1N1 or pandemic influenza
AND Topic: morbidity or mortality or disease burden or hospitalization
AND Topic: children or infant
From 1995 to Dec 31 2018.

Chongqing VIP
Title or key words: (children or infant)
AND (morbidity and mortality or mortality cases or hospitalization or disease burden)
AND (influenza virus or H1N1 or pandemic influenza)
AND (respiratory tract infection or respiratory infection or pneumonia or bronchiolitis or lung infection or severe pneumonia or infectious bronchiolitis)
AND (1995 to Dec 31 2018).

No. of included studies by outcome

Supplementary table 1: No. of included studies reporting incidence rates (n = 38)

	No. of studies			
	**IFV-episodes**	**IFV-ALRI**	**IFV-severe ALRI**	**IFV-very severe ALRI**
All	27	12	14	4
New	21	7	5	4
From RSV GEN	9	10	12	4
Age group				
0-5 months	10	5	12	4
6-11 months	3	5	4	4
12-59 months	5	6	4	3
0-59 months*	18 (6)	12 (6)	7 (3)	4 (1)
Developing countries	14	8	14	4
World Bank income level†				
LICs	8	0	2	0
LMICs	5	7	11	3
UMICs	1	1	1	1
HICs	13	4	0	0
WHO region‡				
AFR	5	1	2	1
AMR	4	2	1	1
EMR	0	1	3	0
EUR	5	2	0	0
SEAR	7	5	8	2
WPR	6	1	0	0
After the 2009 influenza pandemic	18	6	11	3
Quality assessment§				
Low risk of bias in study design	24	12	14	4
Low risk of bias in patient groups excluded	20	8	14	4
Low risk of bias in case definition	10	9	9	4
Low risk of bias in sampling strategy	22	9	14	4
Low risk of bias in diagnostic test	23	11	12	3

* Data in the parenthesis were the numbers of imputed studies.

† LICs: low income countries; LMICs: lower-middle income countries; UMICs: upper-middle income countries; HICs: high income countries as per World Bank Classification.

‡ AFR: WHO African region; AMR: WHO Region of the Americas; EMR: WHO Eastern Mediterranean region; EUR: WHO European region; SEAR: WHO South-East Asian region; WPR: WHO Western Pacific region.

§ As specified in the quality assessment criteria.

Supplementary table 2: No. of included studies reporting hospitalisation rates and hCFRs (n=122)[*]

	No. of studies			
	**IFV-ALRI (N=96)**	**IFV-ALRI with hypoxemia (N=17)**	**IFV-very severe ALRI (N=31)**	**hCFRs of IFV-ALRI (N=66)**
Reporting data for 0-59 months[†]	91 (7)	17 (3)	31 (3)	59
Including data for three age groups[‡]	59	13[§]	24	28
New studies	45	13	24	28
From RSV GEN	30	13	22	27
Developing countries	45	11	18	23
World Bank income level[**]				
LICs	4	1	2	3
LMICs	16	8	8	7
UMICs	13	2	6	11
HICs	26	2	8	7
WHO region[††]				
AFR	14	5	6	11
AMR	14	4	7	9
EMR	1	0	0	0
EUR	8	1	4	3
SEAR	8	1	4	1
WPR	14	1	3	2
After the 2009 pandemic period	30	9	18	23
Quality assessment[‡‡]				
Low risk of bias in study design	51	12	21	26
Adjustment for healthcare utilization	49	11	18	--
Low risk of bias in patient groups excluded	53	10	18	22
Low risk of bias in case definition	29	12	21	25
Low risk of bias in sampling strategy	39	12	19	20
Low risk of bias in diagnostic test	46	11	19	--
Sampling strategy				
Sampling >=90% of eligible cases	29	7	13	17
Systematic sampling	10	0	0	3
Sampling <90% of eligible cases mainly because of refusal or discharged before specimen collection	6	5	7	4
Sampling <90% of eligible cases with unknown reasons	2[§§]	1	1	2
Unknown proportion of sampling	12	0	3	2

[*] There were 59, 13, 24, and 28 studies reporting hospitalisation rates of IFV-(severe and very severe) ALRI and hCFRs. In the table, we only presented the number of studies reporting data for three age groups - 0-5 m, 6-11 m, 1-4 y unless stated otherwise.

[†] Data in parenthesis were the numbers of imputed studies.

[‡] 0-5 m, 6-11 m, 1-4 y.

[§] The 13 studies with age-stratified data were from developing countries. For industrialised countries, we estimated the hospitalisations using data that not stratified by age in 2 studies.

[**] LICs: low income countries; LMICs: lower-middle income countries; UMICs: upper-middle income countries; HICs: high income countries as per World Bank Classification.

[††] AFR: WHO African region; AMR: WHO Region of the Americas; EMR: WHO Eastern Mediterranean region; EUR: WHO European region; SEAR: WHO South-East Asian region; WPR: WHO Western Pacific region.

[‡‡] As specified in the quality assessment criteria.

[§§] Including one study where testing was ordered by physicians; one study sampling <90% of eligible cases due to lack of resources.

Assessment criteria

Supplementary table 3: Assessment of risk of biases for community-based studies

Category	Description	Risk of bias
Study design	Studies where the cases were prospectively enrolled	Low
	Other studies	High
Patient groups excluded	No exclusions that may affect estimates	Low
	Exclusions that may affect estimates, e.g., any of the following:<br>1. Not including very young children (e.g., neonates).<br>2. Excluding children with high-risk conditions.<br>3. Other exclusions that may affect estimates	High
Case definition	Using common/standard definitions	Low
	Using non-standard/inconsistent definitions	High
Sampling strategy	The proportion of testing is available AND either of the following:<br>1. >=90% of eligible cases have been tested.<br>2. Testing a systematic sample of patients.	Low
	<90% of eligible cases have been tested<br>OR<br>The proportion of eligible cases who have been tested is unavailable.	High
Diagnostic test	PCR or culture;<br>Or using other diagnostic tests, but confirming negative samples with PCR or culture	Low
	1. Other diagnostic tests, e.g., IFA, DFA.<br>2. No mention of diagnostic tests	High

Supplementary table 4: Assessment of risk of biases for hospital-based studies

Category	Description	Risk of bias
Study design	Studies where the cases were prospectively enrolled	Low
	Other studies	High
Adjustment for health utilization (only for hospitalisation rate studies)	Meeting either of the following:<br>1. Including all or main hospitals;<br>2. Not including main hospitals, but adjusting for the proportion of patients admitted in the study hospitals	Low
	Not including main hospitals OR no related description; AND no adjustment for the proportion of patients admitted in the study hospitals	High
Patient groups excluded	No exclusions that may affect estimates	Low
	Exclusions that may affect estimates, e.g., any of the following:<br>1. Not including very young children (e.g., neonates).<br>2. Excluding children with high-risk conditions.<br>3. Other exclusions that may affect estimates	High
Case definition	Using common/standard definitions	Low
	Using non-standard/inconsistent definitions	High
Sampling strategy	The proportion of testing is available AND either of the following:<br>1. >=90% of eligible patients have been tested.<br>2. Testing a systematic sample of patients.	Low
	<90% of eligible cases have been tested<br>OR<br>The proportion of eligible cases who have been tested is unavailable.	High
Diagnostic test (only for hospitalisation rate studies)	PCR or culture;<br>Or using other diagnostic tests, but confirming negative samples with PCR or culture	Low
	1. Other diagnostic tests, e.g., IFA, DFA.<br>2. No mention of diagnostic tests	High
Hypoxemia ascertainment (only for studies providing hypoxemia data)	SpO2 was recorded for all influenza-confirmed cases	Low
	1. SpO2 was recorded for a proportion of influenza-confirmed cases.<br>2. No mention of how many influenza-confirmed cases have been assessed for hypoxemia.	High

Details of sensitivity analyses

In Supplementary table 5, we estimated global hospitalisations and in-hospital mortality by summing up the estimates by country income group where available.

In Supplementary table 6, IFV-ALRI hospitalisations and in-hospital mortality were estimated based on the meta-estimates for 0-59 months. We further estimated hospital admissions and in-hospital mortality before 2009 and after the 2009 influenza pandemic period.

In Supplementary table 7-10, we estimated hospitalisations and in-hospital mortality by the risk of bias and by different settings. Analyses were conducted (1) in studies with low risk of bias in in sampling strategy; (2) in studies with low risk of bias in diagnostic test method (only for hospital admissions); (3) in studies with low overall risk of biases; (4) in studies after the 2009 influenza pandemic period. Moreover, stratified analyses were performed by PCV coverage, and by HIV burden in pregnant women. Data were classified into low (<=60%, arbitrary cut-off) and high PCV coverage (>60%) based on the PCV coverage among general population per year per country[1].

In Supplementary table 11, we estimated hospitalisations of IFV-ALRI with hypoxemia by excluding studies with high risk of bias in hypoxemia ascertainment.

In supplementary table 12, we reported burden estimates for 2015. In meta-regression analysis, we did not find significant difference in hospitalisation rates and hCFRs between studies before 2015 and studies during 2015-2018 (median study year: pre-2014 vs 2014 or later). So we applied the meta-estimates of all studies to the 2015 UN population estimates to yield 2015 estimates.

Supplementary table 5: Global burden estimates by age group and by country income group in 2018

	0-5 m[*]	6-11 m*	12-59 m*	0-59 m[†]
Global hospitalisations of IFV-ALRI (*1,000)	176 (101-311)	158 (83-311)	452 (248-858)	786 (432-1481)
Global in-hospital deaths of IFV-ALRI	4500 (1000-24000)	6000 (2500-17200)	10300 (4300-24900)	20800 (7800 65700)

[*] For each age group, global estimates were calculated by summing up the estimates by country income group; estimates by income strata were in main Table 2 and Table 3.
[†] Estimates in children younger than five years were calculated by summing up the estimates by three age groups (0-5 m, 6-11 m, and 12-59 m).

Supplementary table 6: Sensitivity analyses of hospitalisation rate and hCFR meta-estimates for 0-59 months before and after 2009 influenza pandemic period, and global burden estimates in 2018

	Developing	Industrialised	Global estimates
**IFV-ALRI hospital admissions (all studies)**			
No of studies	62 (3)	29 (4)	
Rates (/1000)	1.3 (0.9-1.7)	0.8 (0.6-1.1)	
Hospital admissions (*1000)	775 (568-1056)	59 (45-78)	834 (613-1133)
**IFV-ALRI hospital admissions (before 2009 pandemic)**			
No of studies	16 (1)	19 (3)	
Rates (/1000)	1.8 (1-3.6)	1.0 (0.8-1.2)	
Hospital admissions (*1000)	1095 (579-2070)	70 (57-85)	1164 (636-2155)
**IFV-ALRI hospital admissions (after 2009 pandemic)**			
No of studies	52 (2)	12 (2)	
Rates (/1000)	1.2 (0.9-1.7)	0.6 (0.4-1)	
Hospital admissions (*1000)	730 (532-1002)	42 (27-66)	772 (559-1067)
**IFV-ALRI in-hospital deaths (all studies)**			
No of studies	43	16	
hCFR (%)	1.9 (1.2-3.2)	0.5 (0.3-0.7)	
Deaths	15000 (8400-26600)	300 (200-500)	15300 (8500-27100)
**IFV-ALRI in-hospital deaths (before 2009 pandemic)**			
No of studies	12	7	
hCFR (%)	1.5 (0.6-3.9)	0.4 (0.2-0.7)	
Deaths	16400 (5300-49900)	300 (100-500)	16700 (5400-50400)
**IFV-ALRI in-hospital deaths (after 2009 pandemic)**			
No of studies	35	9	
hCFR (%)	2.0 (1.1-3.6)	0.5 (0.3-0.6)	
Deaths	14600 (7400-28300)	200 (100-400)	14800 (7500-28700)

Supplementary table 7: Sensitivity analyses of IFV-ALRI hospitalisation rate meta-estimates by age group and by study quality, and global hospitalisations in 2018.

	0-5 m		6-11 m		12-59 m		Hospital admissions in 0-59 m
	No *	Rate †	No	Rate	No	Rate	
**Main analysis - in all studies**							
Developing	24	2.8 (1.8-4.3)	21	2.7 (1.7-4.3)	35	0.9 (0.6-1.5)	776 (494-1220)
Industrialised	11	3.7 (2.7-5.3)	7	2.6 (0.9-7.6)	13	0.9 (0.4-1.8)	94 (48-194)
Global							870 (543-1415)
**In studies with low risk of bias in sampling strategy**							
Developing	18	2.8 (1.6-4.8)	15	2.1 (1.2-3.7)	27	0.8 (0.5-1.3)	691 (390-1224)
Industrialised	6	3.8 (2.8-5.1)	4	2 (0.4-10.8)	6	1.1 (0.3-4)	102 (39-330)
Global							793 (430-1553)
**In studies with low risk of bias in test method**							
Developing	23	2.7 (1.7-4.3)	20	2.7 (1.6-4.5)	32	0.9 (0.6-1.5)	770 (482-1232)
Industrialised	5	4.9 (3-7.7)	3	2.2 (0.5-9.8)	8	0.9 (0.5-1.5)	100 (54-209)
Global							870 (536-1441)
**In studies with low risk of biases in >=5 categories‡**							
Developing	19	2.4 (1.4-4.1)	17	2.2 (1.3-3.9)	28	0.8 (0.5-1.3)	672 (407-1112)
Industrialised	3	3.8 (2.6-5.6)	··	··	2	0.5 (0.1-1.7)	··
Global							··

* No: number of studies.
† Rate: hospitalisation rate per 1,000 children per year.
‡ We assessed six domains for hospitalisation rate studies, and set the cut-off value of low risk of biases in five domains to include at least 75% of studies.

Supplementary table 8: Sensitivity analysis of hospitalisation rate meta-estimates of IFV-ALRI by age group, and global hospitalisations in 2018.

	0-5 m		6-11 m		12-59 m		Hospital admissions in 0-59 m
	No *	Rate †	No	Rate	No	Rate	
**Main analysis - in all studies**							
Developing	24	2.8 (1.8-4.3)	21	2.7 (1.7-4.3)	35	0.9 (0.6-1.5)	776 (494-1220)
Industrialised	11	3.7 (2.7-5.3)	7	2.6 (0.9-7.6)	13	0.9 (0.4-1.8)	94 (48-194)
Global							870 (543-1415)
**In studies providing data for all three age groups**							
Developing	19	2.5 (1.4-4.3)	19	2.7 (1.6-4.6)	19	1 (0.5-1.9)	806 (437-1494)
Industrialised	5	4.5 (2.5-8.1)	5	1.9 (0.7-5.4)	5	0.8 (0.3-1.9)	89 (40-205)
Global							895 (477-1698)
**After the 2009 pandemic period**							
Developing	20	3 (1.8-4.9)	17	2.8 (1.6-5)	27	0.8 (0.5-1.5)	746 (436-1280)
Industrialised	4	2.8 (1.2-6.5)	3	1.8 (0.4-9)	4	0.6 (0.2-1.9)	65 (22-207)
Global							812 (458-1487)
**By HIV burden**[2]							
Developing - Global plan priority countries	12	2.0 (1.2-3.4)	11	1.9 (1.5-2.5)	12	0.5 (0.4-0.8)	201 (140-291)
Developing – other countries	12	3.7 (2.0-7.0)	10	3 (1.3-7.3)	23	1.2 (0.6-2.3)	583 (292-1169)
Industrialised	11	3.7 (2.7-5.3)	7	2.6 (0.9-7.6)	13	0.9 (0.4-1.8)	94 (48-194)
Global							877 (481-1655)
**By PCV coverage**‡[1]							
Developing countries							
PCV >60%	11	1.5 (0.9-2.5)	10	1.2 (0.8-1.9)	18	0.6 (0.4-1.0)	220 (138-351)
PCV <=60%	18	2.5 (1.5-4.2)	16	2.6 (1.4-4.7)	22	1.2 (0.6-2.3)	539 (289-1009)
**Subtotal in developing countries**							759 (428-1360)
Industrialised countries							
PCV >60%	3	2.6 (0.9-7.1)	2	2.4 (0.3-17.2)	3	0.8 (0.2-3.1)	76 (19-335)
PCV <=60%	10	3.4 (2.5-4.5)	6	1.6 (0.5-4.9)	11	0.9 (0.4-1.9)	12 (6-26)
**Subtotal in industrialised countries**							88 (25-361)
**Global**							847 (453-1720)

* No: number of studies.
† Rate: hospitalisation rate per 1,000 children per year.
‡ The cut-off value was arbitrarily chosen.

Supplementary table 9: Sensitivity analyses of IFV-ALRI in-hospital case fatality ratio (hCFR) meta-estimates by age group and by study quality, and global burden estimates in 2018.

	0-5 m		6-11 m		12-59 m		
	**No** [*]	**hCFR (%)**	**No**	**hCFR (%)**	**No**	**hCFR (%)**	**Global in-hospital mortality in 0-59 m**
**Main analysis - in all studies reporting by three age groups**							
Developing	23	3.1 (1.3-6.9)	23	2 (0.6-6.2)	23	1.4 (0.7-2.8)	14800 (5700-39000)
Industrialised	5	0.3 (0-6.9)	5	0.9 (0.2-3.4)	5	0.4 (0.1-2.7)	500 (100-4800)
Global							15300 (5800-43800)
**In studies with low risk of bias in sampling strategy**							
Developing	18	3.5 (1.5-8)	18	2.8 (0.9-8.3)	18	2.3 (1.3-4.1)	18700 (7300-48300)
Industrialised	2	‥	2[†]	‥	2	0.8 (0-26.4)	‥
Global							‥
**In studies with low risk of biases in >=3 categories**[‡]							
Developing	22	3.5 (1.6-7.6)	22	2.9 (1.1-7.5)	22	2.2 (1.3-3.6)	17700 (7600-41700)
Industrialised	2	‥	2†	‥	2	0.8 (0-26.4)	‥
Global							‥

[*] No: number of studies.
[†] Both studies reported zero IFV-ALRI death.
[‡] We assessed four domains for hCFR studies, and set the cut-off value of low risk of biases in three domains to include at least 75% of studies.

Supplementary table 10: Sensitivity analyses of IFV-ALRI in-hospital case fatality ratio (hCFR) meta-estimates by age group, and global burden estimates in 2018.

	0-5 m		6-11 m		12-59 m		
	**No** *	**hCFR (%)**	**No**	**hCFR (%)**	**No**	**hCFR (%)**	**In-hospital mortality in 0-59 m**
**Main analysis - in all studies reporting by three age groups**							
Developing	23	3.1 (1.3-6.9)	23	2 (0.6-6.2)	23	1.4 (0.7-2.8)	14700 (5600-38700)
Industrialised	5	0.3 (0-6.9)	5	0.9 (0.2-3.4)	5	0.4 (0.1-2.7)	400 (100-4700)
Global							15100 (5700-43300)
**After the 2009 pandemic period**							
Developing	22	3.5 (1.5-8.1)	22	1.9 (0.5-6.9)	2	1.4 (0.6-3.1)	15200 5200-44900)
Industrialised	5	0.3 (0-6.9)	5	0.9 (0.2-3.4)	5	0.4 (0.1-2.7)	300 (0-4100)
Global							15500 (5300-49000)
**By HIV burden**[2]							
Developing - Global plan priority countries	11	3.2 (1.2-8.7)	11	2.8 (0.9-8.9)	11	2.1 (1.1-4.2)	5100 (2000-13700)
Developing – other countries	12	2.8 (0.7-10.9)	12	1.0 (0.1-12.3)	12	1.0 (0.3-3.1)	8200 (1800-42900)
Industrialised	5	0.3 (0-6.9)	5	0.9 (0.2-3.4)	5	0.4 (0.1-2.7)	500 (100-4800)
Global							13800 (3800-61600)
**By PCV coverage**†1							
Developing countries							
PCV <=60%	18	2.5 (0.5-11.5)	18	3.4 (1.2-9.0)	18	1.3 (0.4-3.9)	10600 (4200-40900)
PCV >60%	15	2.9 (1.0-8.0)	15	1.4 (0.2-8.8)	15	1.8 (0.7-4.8)	4300 (1300-15100)
**Subtotal in developing countries**							14900 (4100-55700)
Industrialised countries							
PCV <=60%	2	2.0 (0.3-12.6)	2	··	2	1.0 (0-23.9)	··
PCV >60%	3	··	3	1.0 (0.2-3.8)	3	0.3 (0-2.5)	··
**Subtotal in industrialised countries**							··
**Global**							··

* No: number of studies.
† The cut-off value was arbitrarily chosen.

Supplementary table 11: Sensitivity analyses of hospitalisation rate meta-estimates of IFV-ALRI with hypoxemia by age group, and global burden estimates in 2018.

	0-5 m		6-11 m		12-59 m		
	**No** *	**Rate**†	**No**	**Rate**	**No**	**Rate**	**Hospital admissions of IFV-ALRI with hypoxemia in 0-59 m**
**Main analysis – in studies with data for three age bands**							
Developing	10	0.9 (0.6-1.4)	10	0.8 (0.5-1.1)	9	0.2 (0.1-0.3)	202 (126-326)
Industrialised		‥		‥	2	0 (0-1.1)	‥
Global							‥
**In studies with low risk of bias in hypoxemia ascertainment**							
Developing	7	1.0 (0.6-1.6)	7	0.8 (0.4-1.5)	7	0.2 (0.1-0.4)	208 (125-358)
Industrialised		‥		‥		‥	‥
Global							‥
**In studies reporting data by three age bands**							
Developing	8	1 (0.6-1.6)	8	0.8 (0.6-1.0)	8	0.2 (0.1-0.4)	206 (111-386)
Industrialised		‥		‥		‥	‥
Global							‥

* No: number of studies.
† Rate: hospitalisation rate per 1,000 children per year.

Supplementary table 12: Global burden estimates by age group and by country income group in 2015

	0-5 m[*]	6-11 m*	12-59 m*	0-59 m[†]
Hospitalisations of IFV-ALRI (*1,000)				
Developing	172 (112–266)	166 (105–264)	430 (273–678)	768 (489–1,207)
Industrialised	25 (18–35)	18 (6–51)	51 (24–107)	93 (48–193)
Global estimates	197 (130–301)	184 (111–315)	480 (296–785)	861 (537–1,400)
In-hospital deaths of IFV-ALRI				
Developing	5,300 (2,100–13,500)	3,300 (900–11,500)	6,000 (2,600–13,600)	14,700 (5,600–38,700)
Industrialised	100 (0–2,800)	200 (0–900)	200 (0–1,200)	400 (100–4,700)
Global estimates	5,400 (2,100–16,100)	3,500 (1,000–12,400)	6,200 (2,600–14,800)	15,100 (5,700–43,300)

[*] For each age group, global estimates were calculated by summing up the estimates by country development status; rates by age and by region are available in main Table 2 and Table 3. 2015 UN population estimates were used.

[†] Estimates in children younger than five years were calculated by summing up the estimates by three age groups (0-5 m, 6-11 m, and 12-59 m). For hospitalisations of IFV-ALRI with hypoxemia,

Details of overall mortality estimation

We estimated overall IFV-ALRI deaths by applying the inflation factor (factor of overall IFV-ALRI deaths versus in-hospital deaths) to the robust IFV-ALRI in-hospital mortality estimates.

The details about inflation factor estimation in industrialised countries are in the main text. For developing countries, we invited investigators from the INDEPTH Network to provide two types of data: (1) population-based pneumonia deaths and local concurrent influenza circulation data from severe acute respiratory infection (SARI) surveillance, both reported on a monthly basis; (2) the numbers of pneumonia deaths occurring in- and out-of-hospital for the same observation period, separately. For the (1) type data, we requested the number of pneumonia deaths to be at least 60 over three consecutive years. In addition to the INDEPTH Network, we identified one study in Bangladesh in RSV GEN with eligible (1) type data. So altogether we identified six sites in developing countries with relevant data. The inflation factor was estimated using three different approaches in developing countries. Here we listed the details for each approach.

Approach 1 (main analysis) - we divided the overall pneumonia deaths by in-hospital pneumonia deaths, and used the median value across countries to represent the inflation factor for IFV-specific ALRI deaths as in Supplementary table 13. Using this approach, we assumed that the relative contribution of influenza in ALRI deaths was the same for the hospitalised deaths and those not hospitalised.

Supplementary table 13: Approach 1 - Inflation factor for IFV-ALRI mortality in developing countries, and overall IFV-ALRI mortality among children under five years

	Site	Ratio of in- and out-of-hospital pneumonia deaths over in-hospital deaths in children under five years	Inflation factor (average ratio)	Overall IFV-ALRI mortality
**Developing**	Nairobi, Kenya (urban)	1.7	2.3	34100 (UR 13100 89700)
	Siaya, Kenya (rural)	3.5		
	Nouna, Burkina Faso (rural)	1.5		
	Dodowa, Ghana (rural)	2.1		
	Manhiça, Mozambique (peri-urban, rural)	2.8		
	Agincourt, South Africa (rural)	2.3		
**Industrialised**	USA	1.6	1.6	700 (100-7700)
**Global**				34800 (13200-97200)

Approach 2 and 3 shared a same strategy in applying (c) the proportion of IFV-ALRI deaths among all-cause ALRI deaths to (d) national all-cause pneumonia deaths, and then divided by (f) the national IFV-ALRI in-hospital deaths, to back calculate the inflation factor. (f) The national IFV-ALRI in-hospital deaths, were estimated using the location-matched hospitalisation rate and hCFR data (site-matched data in Siaya, Kenya; Manhiça, Mozambique; country-matched data in South Africa). For Bangladesh, we used the hCFR meta-estimate in developing countries because site- or country-matched data were unavailable (Supplementary table 13-15).

For Approach 2 and 3, our first step was to estimate the IFV-ALRI in-hospital deaths using population in 2015 (U5 population), the hospitalisation rate (U5 Hos Rate), and hCFR (U5 hCFR) per country for 0-59 months where available. For countries the hospitalisation rates and hCFRs were unavailable, we applied the meta-estimates in developing countries to the country-specific population (Supplementary table 14).

$$\text{Country IFV-ALRI in-hospital deaths} = \text{U5 population} * \text{U5 Hos Rate} * \text{U5 hCFR}$$

Approach 2 - the excess mortality approach depends on well-defined influenza seasons. A notable difference between Approach 2 and 3 was that different methods were used to estimate (c) the proportion of IFV-ALRI deaths among all-cause deaths. Approach 2 was similar to the previous model we have used wherein the excess pneumonia deaths during influenza season was attributed to influenza. Whenever there is an overlap between influenza and RSV season, we proportionately attributed excess pneumonia deaths within influenza season to the two pathogens. Assumptions included (1) the association of influenza with pneumonia deaths was not

confounded by other pathogens; (2) for IFV and RSV, the degree of association between the virus activity and the number of pneumonia deaths caused by the viruses was same. IFV (RSV) season was defined as at least 10 samples were tested and at least 10% of tested samples were positive. There are no clear influenza seasons in the two sites in Bangladesh and Kenya, so we are unable to estimate the inflation factor using this approach. The impact of influenza or any pathogen varies across years. We may observe negative excess pneumonia deaths with the IFV season in a specific year when other pathogens circulate outside IFV and RSV seasons, and cause more pneumonia deaths. Therefore, to account for this, and to estimate the average annual impact of seasonal IFV on pneumonia deaths, the negative excess deaths for one particular year were not set to zero. With the IFV-ALRI in-hospital deaths, we estimated the inflation factor as in Supplementary table 15.

Supplementary table 14: In-hospital deaths data and population-based pneumonia deaths data by site

Site and period (population-based pneumonia deaths and IFV activity)	Site and period (in-hospital IFV-ALRI deaths) (f)
Nairobi (Kenya), 2008, 2010-2015	Siaya (Kenya), 2010-2014
Siaya (Kenya), 2010-2016	Siaya (Kenya), 2010-2014
Bangladesh, 2010-2012	Meta-estimates in developing countries
Manhiça, Mozambique, 2012-2016[*3]	Manhiça, Mozambique, 2011-2014
Agincourt, South Africa, 2010-2015	Paarl, Soweto, Klerksdorp, Pietermaritzburg, 2010-; Soweto, 1998-2005

[*] Local IFV and RSV activity data in Manhiça, Mozambique were available during Jan 2012-June 2014, but unavailable during July 2014-Dec 2016. For IFV, we used the data in neighbouring areas (distance <100 kilometres). For RSV, the average proportions of RSV during Jan 2012-Dec 2013 were extrapolated to Jan-Dec 2014.

Supplementary table 15: Approach 2 - Inflation factor for IFV-ALRI mortality in developing countries, and overall IFV-ALRI mortality among children under five years

Year	Country	IFV-PNE death at the site (a)	PNE death at the site (b)	Proportion of IFV-PNE deaths (c=a/b, %)	2015 U5 PNE deaths (d) [4]	Overall IFV-ALRI deaths (e = d*c)	IFV-ALRI in-hospital deaths (f)	Inflation factor (h = e/f)	Average inflation factor	Global overall IFV-ALRI mortality*
2010-2012	Bangladesh	-33.2	208	--	17408	--	361	--	3.0	45200 (UR 17200-124500)
2010-2015	South Africa	4.6	125	3.7	7105	264	98	2.7		
2012-2016	Mozambique	4.5	151	3.0	11769	351	104	3.4		
2008; 2010-2015	Kenya (Nairobi)	-13.5	244	--	10584	--	142	--		
2010-2016	Kenya (Siaya)	50.1	1261	4.0	10584	421	142	3.0		

* Estimated by summing up overall mortality in developing (inflation factor = 3.0) and industrialised countries (inflation factor = 1.6)

Approach 3 - we adopted a new methodology. In this approach, we considered that the number of IFV-ALRI deaths was associated with the influenza activity (i.e., the proportion of IFV in ALRI cases), the risk of dying from IFV-ALRI compared with the risk of dying for non-IFV-ALRI, and the number of ALRI deaths. Influenza activity and ALRI deaths can vary across months through a year. Thus, the number of IFV-ALRI deaths could be estimated by combining the proportion of IFV in ALRI cases, ALRI deaths, and the risk of dying from IFV-ALRI on a monthly basis. We considered that the risk of dying from IFV-ALRI was constant through a year.

So to estimate (c) the proportion of IFV-ALRI deaths among ALRI deaths, specifically we combined (1) the monthly proportion of IFV in SARI cases, (2) monthly pneumonia deaths; (3) the risk of dying from IFV compared with non-IFV-ALRI - the ratio of hCFR of IFV-ALRI deaths over non-IFV-ALRI deaths. The samples at the sites were mostly from children under five years with SARI in local surveillance, we considered that the proportion of IFV in these samples was a reasonable proxy of that in pneumonia cases. Based on eight hospital-based studies in developing countries, we estimated that the hCFR of IFV-ALRI was lower compared with non-IFV-ALRI in children under five years (1.9 [95%CI 0.5-6.6)] vs 3.4 [95%CI 1.6-7.3], Supplementary table 15-16). Thus the risk of dying from IFV compared with non-IFV-ALRI was 0.56 (1.9/3.4).

After calculating the proportion, we applied it to pneumonia deaths (U5 PNE) in 2015 per country to estimate the country-specific IFV-ALRI overall deaths (Supplementary table 16). Based on pneumonia deaths under five years for the ith month (MonPNEi), IFV positivity for the ith month (PropIFVi, %), and the relative hCFR (0.56), country-specific IFV-ALRI overall deaths were estimated as:

$$\text{U5 PNE} * \left(\sum_1^k \left(\frac{\text{PropIFVi}* 0.56}{(\text{PropIFVi}*0.56+(1-\text{PropIFVi})*1.00} * \text{MonPNEi}\right) / \sum_1^k \text{MonPNEi}\right)$$

In Approach 3, the bias mainly came from the ratio of CFR of IFV-ALRI deaths over that for non-IFV-ALRI deaths, which was estimated based on eight hospital-based studies from RSV GEN from low- and middle-income countries. We were unable to find an alternative estimate in other studies. However, the 2015 CFR estimate of all-cause overall severe pneumonia (4·2%) and our CFR estimate of hospitalised IFV-ALRI (1·9%) suggested a lower CFR ratio (0·45) which would be reflected in a lower inflation factor, and a lower mortality estimate.[5]

Supplementary table 16: Approach 3 - Inflation factor for IFV-ALRI mortality in developing countries, and overall IFV-ALRI mortality among children younger than five years [*]

Year	Country	IFV-PNE death at the site (a)	PNE death at the site (b)	Proportion of IFV-PNE deaths (c=a/b, %)	2015 U5 PNE deaths (d) [4]	Overall IFV-ALRI deaths (e = d*c)	IFV-ALRI in-hospital deaths (f)	Inflation factor (h = e/f)	Average inflation factor	Global overall IFV-ALRI mortality[†]
2010-2012	Bangladesh	8.6	208	4.1	17408	720	361	2.0		
2010-2015	South Africa	5.4	125	4.3	7105	307	98	3.1		
2012-2016	Mozambique	5.9	151	3.9	11769	460	104	4.4	3.4	51100 (UR 19500-140400)
2008; 2010-2015	Kenya (Nairobi)	14.6	244	6.0	10584	633	142	4.5		
2010-2016	Kenya (Siaya)	69.8	1261	5.5	10584	586	142	4.1		

[*] PNE: pneumonia. IFV: influenza virus. U5: in children under five years.
[†] Estimated by summing up overall mortality in developing (inflation factor = 3.4) and industrialised countries (inflation factor = 1.6)

Supplementary table 17: Information of tested/untested hospitalised ALRI cases/deaths in eight studies in low- and middle-income countries with available data*.

Location (Study period)	Age	IFV-ALRI deaths	IFV-ALRI cases	ALRI deaths tested negative for IFV	ALRI cases tested negative for IFV	Untested ALRI deaths	Untested ALRI cases	Non-IFV-ALRI deaths	Non-IFV-ALRI cases
Gambia; 2011-2013	0-5 m	0	17	5	239	0	3	5	242
Morocco; 2010-2011	0-5 m	0	3	14	125	0	0	14	125
Mozambique; 2011-2014	0-5 m	0	5	5	108	0	1	5	109
South Africa; 1998 to 2005	0-5 m	1	28	92	934	17	42	109	976
South Africa; 2011-2013	0-5 m	0	18	17	437	0	3	17	440
Thailand; 2005-2011	0-5 m	0	34	5	669	26	1961	31	2630
Togo; 2011-2013; 2014-2015	0-5 m	0	1	2	33	1	5	3	38
Zambia; 2011-2013	0-5 m	3	11	58	310	4	6	62	316
Gambia; 2011-2013	6-11 m	0	4	5	135	2	5	7	140
Morocco; 2010-2011	6-11 m	0	9	8	126	0	0	8	126
Mozambique; 2011-2014	6-11 m	1	7	2	90	0	4	2	94
South Africa; 1998 to 2005	6-11 m	2	20	24	585	5	21	29	606
South Africa; 2011-2013	6-11 m	1	15	13	211	0	0	13	211
Thailand; 2005-2011	6-11 m	0	110	3	1437	22	3846	25	5283
Togo; 2011-2013; 2014-2015	6-11 m	0	5	0	18	0	2	0	20
Zambia; 2011-2013	6-11 m	1	7	28	139	3	3	31	142
Gambia; 2011-2013	12-59 m	0	12	7	219	3	4	10	223
Morocco; 2010-2011	12-59 m	1	16	7	510	0	0	7	510
Mozambique; 2011-2014	12-59 m	0	11	3	190	2	6	5	196
South Africa; 1998 to 2005	12-59 m	1	50	18	985	10	56	28	1041
South Africa; 2011-2013	12-59 m	1	21	5	239	0	0	5	239
Thailand; 2005-2011	12-59 m	1	559	7	5086	30	15811	37	20897
Togo; 2011-2013; 2014-2015	12-59 m	0	14	0	84	1	3	1	87
Zambia; 2011-2013	12-59 m	2	13	21	144	2	5	23	149

* Only including studies testing >=90% of ALRI cases.

Supplementary table 18: hCFR meta-estimates in ALRI cases those were positive for IFV, negative for IFV, and in those untested ALRI cases. *

	hCFR in IFV cases (%)	hCFR in IFV negative cases (%)	hCFR in non-test cases (%)	hCFR in all non-IFV cases (%)	hCFR in tested cases (IFV positive and negative) (%)
0-5 m	1.1 (0.1-19.4)	5.0 (2.5-10.0)	10.4 (1.9-41.1)	5.5 (2.8-10.5)	4.9 (2.3-9.8)
6-11 m	3.1 (0.8-11.0)	3.1 (1.2-8.1)	11.1 (0.9-62.9)	3.7 (1.6-8.5)	3.2 (1.2-8.1)
12-59 m	1.4 (0.4-5.6)	1.4 (0.5-3.9)	16.4 (2.5-59.9)	2.0 (0.8-4.8)	1.5 (0.5-3.9)
0-59 m	1.9 (0.5-6.6)	2.8 (1.2-6.5)	13.5 (3.1-43.7)	3.4 (1.6-7.3)	2.8 (1.1-6.5)

* Meta-estimates were based on the data in eight studies in low- and middle-income countries providing relevant data by three age bands and testing >=90% of eligible ALRI cases.

Details of denominator scaling

Before meta-analyses, we extracted the number of cases and for each study, and scaled the population denominator by applying the original denominator to the proportion of eligible cases who were tested. In published studies to avoid underestimating rates, the number of cases are usually scaled (the observed case number divided by the proportion of eligible cases who are tested). The rates would be the same when we scaled the case number (Formula 1) and scaled the denominator (Formula 2). However, we considered the scaled denominator could better reflect the true size of each study, and the weight of each study in meta-analyses than the original denominator.

Formula 1 - scaling the case number:

Rate = $(\text{No. of observed cases}/\text{Proportion of test})/\text{Original denominator}$

Formula 2 - scaling the denominator:

Rate = $\text{No. of observed cases}/(\text{Proportion of test} * \text{Original denominator})$

Rates are same in Formula 1 and 2. The difference lies in that the original denominator would be used in meta-analyses in Formula 1; while in Formula 2, the scaled denominator (Proportion of test * Original denominator) would be used as input data in meta-analyses.

As in Supplementary table 2, all studies were categorised into five groups based on the sampling strategy. For example, of the 59 studies in the main analyses for IFV-ALRI hospitalisation rates, 49% (29/59) of studies sampled and tested at least 90% of eligible cases, 17% (10/59) sampled and tested cases systematically, 14% (8/59) sampled and tested <90% of eligible cases mainly because of refusal (10%), and without reporting any reasons (3%). Additionally, in 20% (12/59) of studies, the proportions of test in eligible cases are not reported. We did not scale denominators in the studies without the information of proportions of test.

Data imputation

For the community setting, data on incidence rates were insufficient for analysis by narrow age groups. So in this setting we analysed data for the overall age band (0-59 months). Not all studies reported data for 0-59 months; other age groups commonly reported were 0-11 months, 0-23 months. Thus, we decided to impute rates for missing 0-59 months. Our primary focus was to impute incidence rates; hospitalisation rates for missing 0-59 months were also imputed. The imputed hospitalisation rates were only analysed in sensitivity analysis; we reported the hospitalisations from age-stratified analysis as the main analysis which did not require imputed data. The imputation was done as follows. First, we estimated denominators according to WHO life tables by World Bank income level. Based on the $_nq_x$ (the probability of dying between age n and x+n) for both sexes in 2013, we computed the ratios between population estimates in age groups (0-11 m, 0-23 m, 0-59 m).[6] Because the information is only available for 0-11 months and 12-59 months, we assumed that the probability of dying within 12-59 months was same. By multiplying the specific ratios by the base denominator, we estimated the denominator for 0-59 months.

After denominator estimation, for studies missing case numbers, we imputed case numbers using a multiple imputation approach assuming the rates for 0-59 m were missing at random (Supplementary figure 2).[7] Briefly, we firstly did meta-analyses to estimate the rate ratios between 0-59 m and one of other age groups (i.e., 0-11 m, 0-23 m, 0-35 m). Here, 0-59 m and any one of other age groups made one comparison (e.g., 0-59 m vs 0-11 m). For each comparison we only performed meta-analysis when there were three or more studies. We assumed the pooled rate ratio followed a log-normal distribution, and simulated 100 samples using the pooled rate ratio and standard error for each comparison. Then based on the samples of rate ratios, we imputed rates for 0-59 months based on the rates in the reference group and the corresponding rate ratios; using the denominator and imputed rate we calculated the case number. This gave us 100 datasets of imputed case number. We did meta-analysis for each dataset, and combined the meta-estimates together using the Rubin's rules.[8,9]

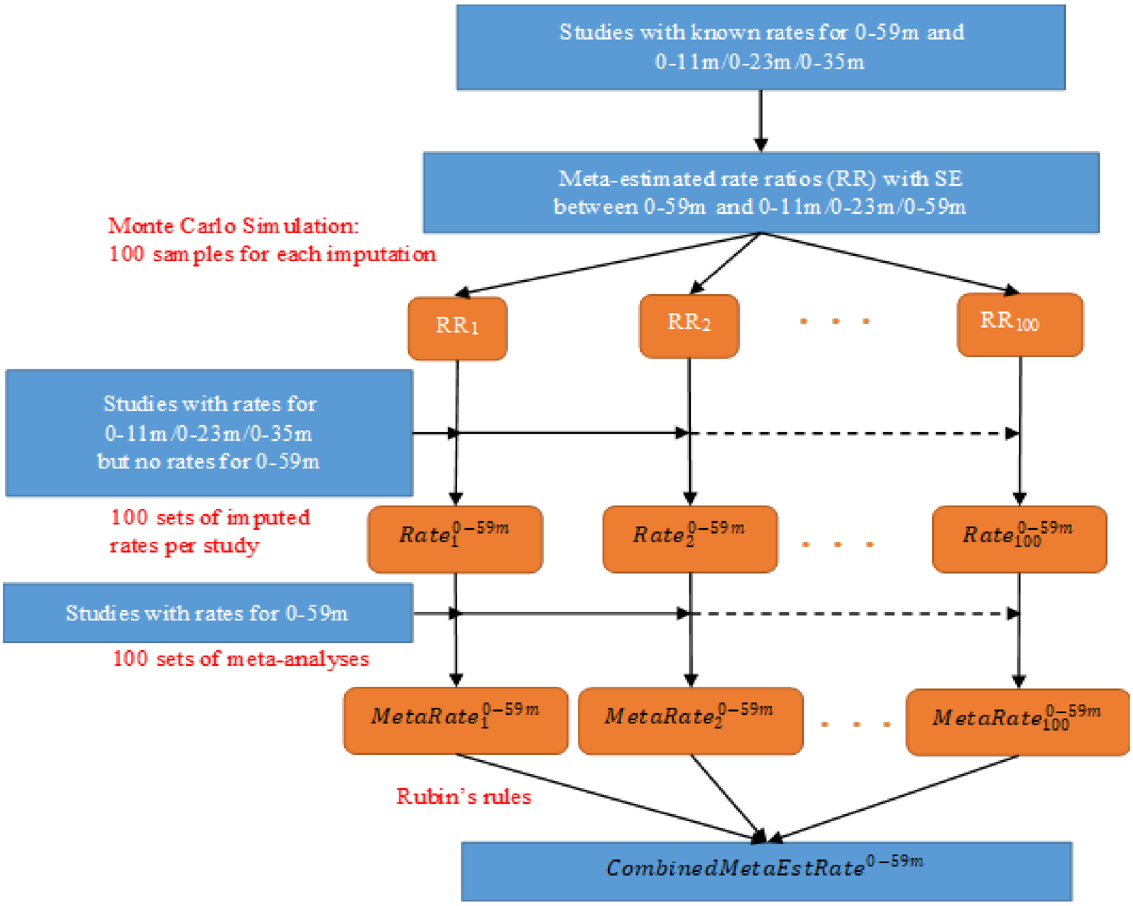

Supplementary figure 2. Imputing rates for 0-59 months using a multiple imputation approach.

In our previous analysis, we imputed rates for 0-59 months using median rate ratios.[10] Briefly, we imputed rates based on median rate ratios and the data in the reference age group. Unlike the multiple imputation approach,

there was one imputed number for one study. The results from the median rate ratio approach and the multiple imputation approach are in Supplementary table 19.

Supplementary table 19: Incidence/hospitalisation rate meta-estimates for 0-59 months when excluding and including imputed data using the median rate ratio approach and the multiple imputation approach.

	Excluding imputed data		Including imputed data (median rate ratio approach)		Including imputed data (multiple imputation approach)
	No*	Rate†	No	Rate	Rate
**In the community setting‡**					
IFV-episodes	5	142.9 (69.2-271.9)	8 (3)	135.5 (84-211.3)	175.2 (101.5-302.3)§
IFV-ALRI	5	15.4 (10-23.6)	8 (3)	15.8 (10.5-23.9)	15.6 (10.3-22.6)
IFV-severe ALRI	4	2.4 (0.6-9.6)	7 (3)	2.3 (1-5.2)	2.4 (1.0-5.6)
IFV-very severe ALRI	3	0.7 (0.1-3.9)	4 (1)	0.7 (0.2-2.9)	0.7 (0.2-2.7)
**Hospital-based studies (IFV-ALRI)**					
Developing	59	1.3 (1.0-1.8)	62 (3)	1.3 (0.9-1.7)	1.3 (0.9-1.7)
Industrialised	25	0.9 (0.7-1.2)	29 (4)	0.8 (0.6-1.1)	0.8 (0.6-1.1)

* No: number of studies. Data in parentheses was the number of imputed studies.
† Rate: per 1,000 children per year. Incidence rate for community-based studies; hospitalisation rate for hospital-based studies.
‡ Rates in developing countries.
§ We estimated a higher rate ratio between 0-59 m and 0-11 m (or 0-23 m) using the multiple imputation approach compared with the median rate ratio approach. This was mainly driven by one large study in Bangladesh in which the incidence rates of IFV-episodes increased with age. Unlike the median rate ratio, the rate ratio from a meta-analysis was influenced by study sizes. Thus, the higher rate ratio translated to the higher incidence rates after imputation.

Tables for description of included studies and quality of included studies

Supplementary table 20: Description of studies reporting IFV-episodes incidence rates in children under five years (per 1,000 children per year)*†.

ID	Location (reference)	Case Definition	Denominator source	Specimen and diagnostic test	0-27 d	1-2 m	3-5 m	6-11 m	12-23 m	24-59 m	0-5 m	0-59 m
700	Bamako, Mali (Sep 2011-Jan 2014) (Tapia et al, 2016)[11]	Fever; ARI AND Fever	Defined population base	NPS and OPS; PCR	36.1	40.1	133.0	··	··	··	83.1	··
701	IISP sites, USA (Oct 2009-Jul 2013) (Fowlkes et al, 2015)[12]	ILI; ARI AND Fever	Census-derived estimate	Nasal swabs, nasal aspirates, NPS, or OPS; PCR	··	··	··	··	··	13.2	··	9.7
703	Turku, Finland (Oct 2000-May 2002) (Heikkinen et al, 2004)[13]	ARI AND Fever	Defined population base	Nasal swabs; Viral culture and subsequent immunoperoxidase staining with monoclonal antibodies	··	··	··	··	185.3	110.6	··	132.3
801	Senegal (Jan 2012-Dec 2013) (Diene-Sarr et al, 2015)[14]	Fever	Defined population base	NP and oral specimens; PCR	··	··	··	··	··	259.7	45.5	300.0
802	Senegal (Jan 2012-Dec 2013) (Diene-Sarr et al, 2015)[14]	Fever	Defined population base	NP and oral specimens; PCR	··	··	··	··	··	282.1	280	317.2
b_40	Nashville, TN, USA (Aug 1974-Jul 1999) (Neuzil et al, 2002)[15]	ARI; Fever	Defined population base	Nasal wash; Culture and HI or DFA	··	··	··	··	114	81.7	··	95.0
ba_3	Ballabhgarh, India (2001-2005) (Broor et al, 2007)[16]	ARI	Defined population base	NPW; DFA	··	··	··	··	174.2	··	··	··
ba_4	Kamalapur, Bangladesh (Apr 2004-Dec 2007) (Brooks et al, 2010)[17]	ARI AND Fever	Defined population base	NPW; Viral culture and HI	··	··	··	··	78.7	71.5	··	92.7
up_2	Managua, Nicaragua (Sep 2012-Sep 2015) (Gordon and colleagues)	ARI; Fever	Defined population base	Nasal and throat swabs; PCR	41.7	56.5	140.6	244.0	263	··	102.9	··
up_25	Kamalapur, Bangladesh (2008-2015) (Brooks and colleagues)	ARI AND Fever	Defined population base	NPW; PCR and tissue culture	0.4	4.5	23.1	110.6	199.5	274.4	9.3	126.7
up_600	Nepal (April 2011-May 2014) (Omer and colleagues)	ARI AND Fever	Defined population base	NPS; PCR	66.7	105.6	··	··	··	··	94.1	··

* NPA: nasopharyngeal aspirate. NPS: nasopharyngeal swab. NPW: nasopharyngeal wash. OPS: oropharyngeal swab. PCR: polymerase chain reaction. IFA: indirect immunofluorescence assay. DFA: direct immunofluorescence. ELISA: enzyme-linked immunosorbent assay. HI: hemagglutination-inhibition assay. RIDT: rapid influenza diagnostic test. SPIA: solid-phase immunoassay. MN: micro-neutralization assay. TRFIA: time-resolved fluoroimmunoassay. ILI: influenza like illness (fever and either cough or sour throat). ARI: any of respiratory symptoms, including cough, sour throat, rhinitis, pharyngitis, and others.

† Incidence rates were adjusted for the proportion of testing in eligible patients where available.

ID	Location (reference)	Case Definition	Denominator source	Specimen and diagnostic test	0-27 d	1-2 m	3-5 m	6-11 m	12-23 m	24-59 m	0-5 m	0-59 m
up_601	Mali (Sep 2011-Jan 2014) (Omer and colleagues)	ARI AND Fever; Fever	Defined population base	NPS; PCR	40	33.6	··	··	··	··	35.5	··
up_602	South Africa (Mar 2011-May 2013) (Omer and colleagues)	ARI; Fever	Defined population base	NPS; PCR	26.7	111.7	··	··	··	··	86.6	··
229	Nam, Vietnam (2007-2010) (Horby et al, 2012) [18]	ILI	Defined population base	Nasal and Throat swabs; PCR	··	··	··	··	··	··	··	27.8
702	Izu-Oshima Island, Japan (Jan 2009-Mar 2011) (Inamasu et al, 2012) [19]	ILI	Census-derived estimate	NPS; RIDT and PCR	··	··	··	··	··	··	··	180.4
s408	Japan (Dec-Jun, 2004-2008) (Kimura et al, 2011)[20]	ILI	Census-based estimate	NPS or NPA; Rapid antigen test kit	··	··	··	··	··	··	··	124.1
ba_1	Berne, Switzerland (Apr 1999-Dec 2004) (Regamey et al, 2008) [21]	ARI	Defined population base	Nasal swabs; PCR	··	··	··	··	··	··	··	··
e19	India (Aug 2012-Dec 2014) (Kumar et al, 2017) [22]	ARI; Fever	Defined population base	NPA; PCR	··	··	··	··	··	··	··	··
e24	Southwest Finland (Jan 2010-Apr 2012) (Teros-Jaakkola et al, 2017) [23]	ARI; Fever	Defined population base	Nasal swabs; PCR	··	··	··	··	··	··	··	··
e17	USA (Jul 2010-Jun 2014) (Buck et al, 2017) [24]	ARI	Defined population base	NA; NA	··	··	··	··	··	··	··	··
e21	Sydney, Australia (May-Nov 2011) (Li-Kim-Moy et al, 2017) [25]	ARI AND Fever	Defined population base	Nasal and throat swabs; PCR	··	··	··	··	··	··	··	··
e23	Nepal (Apr 2011-Apr 2013) (Steinhoff et al, 2017) [26]	ARI; Fever	Defined population base	Nasal swabs; PCR	··	··	··	··	··	··	181.3	··
1807	Australia (2010-2014) (Sarna et al, 2017) [27]	ARI	Defined population base	Nose swab; PCR	··	··	··	··	··	··	··	··
f037	Romania (2011-2016) (Gefenaite et al, 2018) [28]	ILI	Census-derived estimate	NPS; PCR	··	··	··	··	··	··	··	1.4
f052	Nepal (2011-2013) (Katz et al, 2018) [29]	ARI/Fever	Defined population base	Nasal swab; PCR	··	··	··	··	··	··	190.5	··
f071	Spain (2010-2016) (Oliva et al, 2018) [30]	ILI	Census derived estimates	Respiratory swab; NA	··	··	··	··	··	··	··	24.4
s408	Japan (2004-2008) (Kimura et al et al, 2011)[20]	ILI	Census derived estimates	NPS or NPA; Rapid antigen test	··	··	··	··	··	··	··	124.1

Supplementary table 21: Description of studies reporting IFV-associated ALRI incidence rates in children under 5 years (per 1,000 children per year) *†

ID	Location (reference)	Case Definition	Denominator source	Specimen and diagnostic test	0-27 d	1-2 m	3-5 m	6-11 m	12-23 m	24-59 m	0-5 m	0-59 m
b_11	Multicentre, Germany (Nov 1999- Oct 2001) (Forster et al, 2004) [31]	ALRI; Croup	Census-derived estimate	NPA; PCR	··	··	··	··	12.4	··	··	··
b_40	Nashville, TN, USA (Aug 1974-Jul 1999) (Neuzil et al, 2002) [15]	ALRI	Defined population base	Nasal wash; Culture and HI or DFA	··	··	··	··	10.5	3.9	··	8.5
ba_3	Ballabhgarh, India; rural (2001-2005) (Broor et al, 2007) [16]	ALRI	Defined population base	NPW; DFA	··	··	··	··	58.1	··	··	··
ba_4	Kamalapur, Bangladesh; urban (Apr 2004 - Dec 2007) (Brooks et al, 2010) [17]	ALRI	Defined population base	NPW; Culture and HI	··	··	··	··	70.6	20.3	··	25.4
up_1	Faridabad, Haryana, India; rural (Aug 2012-Aug 2014) (Krishnan and colleagues)	ALRI	Census-derived estimate	OP or nasal specimens; PCR	0	27.8	14.6	19.0	21.7	5.4	18.0	10.9
up_2	Managua, Nicaragua (Sep 2012-Sep 2015) (Gordon and colleagues)	ALRI	Defined population base	Nasal and throat swabs; PCR	0	0	10.4	32.2	17.3	··	5.9	··
up_25	Kamalapur, Bangladesh; urban (2008-2014) (Brooks and colleagues)	ALRI AND No wheeze	Defined population base	NPW; PCR and tissue culture	0	2.3	7.8	29.4	40.0	20.2	3.4	17.9
up_26	Oshikhandass, Pakistan; rural (Apr 2012-Mar 2014) (Rasmussen and colleagues)	ALRI	Defined population base	NPS; PCR	0	0	0	0	17.4	3.8	0	5.8
up_3	Western Province, South Africa (Mar 2012-Dec 2016) (Zar and colleagues)	ALRI	Census-derived estimate	NPS; PCR	21.5	71.4	44.1	24.1	21.3	1.2	50.4	22.8
703	Turku, Finland (Oct 2000-May 2002) (Heikkinen et al, 2004) [13]	ALRI	Defined population base	Nasal swabs; Culture and subsequent immunoperoxidase staining with monoclonal antibodies	··	··	··	··	··	··	··	··
e19	India (Aug 2012-Dec 2014) (Kumar et al, 2017) [22]	ALRI	Defined population base	NPA; PCR	··	··	··	··	··	··	··	··
1807	Brisbane, Australia (2010-2014) (Sarna et al et al, 2017) [27]	ALRI; wheeze	Defined population base	Nose swab; PCR	··	··	··	··	··	··	··	··

* NPA: nasopharyngeal aspirate. NPS: nasopharyngeal swab. NPW: nasopharyngeal wash. OPS: oropharyngeal swab. PCR: polymerase chain reaction. IFA: indirect immunofluorescence assay. DFA: direct fluorescent antibody. ELISA: enzyme-linked immunosorbent assay. HI: hemagglutination-inhibition assay. RIDT: rapid antigen detection test. SPIA: solid-phase immunoassay. MN: micro-neutralization assay. TRFIA: time-resolved fluoroimmunoassay. ARI: any of respiratory symptoms, including cough, sour throat, rhinitis, pharyngitis, and others. ALRI, sALRI, and vsALRI: WHO definition for ALRI, chest wall indrawing ALRI, and very severe ALRI.

† Incidence rates were adjusted for the proportion of testing in eligible patients where available.

Supplementary table 22: Description of studies reporting incidence rates of IFV-associated severe ALRI in children under 5 years (per 1,000 children pr year)*†

ID	Location (reference)	Case Definition	Denominator source	Specimen and diagnostic test	0-27 d	1-2 m	3-5 m	6-11 m	12-23 m	24-59 m	0-5 m	0-59 m
ba_4	Kamalapur, Bangladesh (Apr 2004-Dec 2007) (Brooks et al, 2010) [17]	sALRI	Defined population base	NPW; Viral culture and HI	··	··	··	··	1.7	0	··	0.6
up_1	Haryana, India (Aug 2012-Aug 2014) (Krishnan and colleagues)	sALRI	Census-derived estimate	OP or nasal specimens; PCR	0	13.9	14.6	6.3	6.2	1.6	13.5	4.0
up_2	Managua, Nicaragua (Sep 2012-Sep 2015) (Gordon and colleagues)	sALRI	Defined population base	Nasal and throat swabs; PCR	0	0	0	10.7	4.7	··	0	··
up_25	Kamalapur, Bangladesh (2008-2014) (Brooks and colleagues)	sALRI	Defined population base	NPW; PCR and tissue culture	0	0.4	0.3	1.7	1.4	0.6	0.3	0.7
up_271	Sindh, Pakistan (Oct 2011-June 2014) (Asad and colleagues)	sALRI	Defined population base	NPS; PCR	··	··	··	··	··	··	7.8	··
up_3	Western Province, South Africa (March 2012-Dec 2016) (Zar and colleagues)	sALRI	Census-derived estimate	NPS; PCR	21.5	66.7	33.1	18.1	14.6	0	43.5	17.6
ba_2	Mirzapur, Bangladesh (Oct 1993-Aug 1996) (Hasan et al, 2006) [32]	sALRI	Defined population base	NPA; ELISA	··	··	··	··	··	··	··	··
up_521	Sylhet, Bangladesh (2011-2013) (Saha and colleagues)	pSBI	Defined population estimate	NPS or OPS; PCR	16.4	··	··	··	··	··	··	··
up_522	Karachi, Pakistan (2012-2013) (Saha and colleagues)	pSBI	Defined population estimate	NPS or OPS; PCR	12.2	··	··	··	··	··	··	··
up_523	Matiari, Pakistan (2012-2013) (Saha and colleagues)	pSBI	Defined population estimate	NPS or OPS; PCR	5.3	··	··	··	··	··	··	··
up_524	Vellore, India (2013-2014) (Saha and colleagues)	pSBI	Defined population estimate	NPS or OPS; PCR	7.4	··	··	··	··	··	··	··
up_525	Odisha, India (2013-2014) (Saha and colleagues)	pSBI	Defined population estimate	NPS or OPS; PCR	7.6	··	··	··	··	··	··	··
up_600	Nepal (Apr 2011-May 2014) (Omer and colleagues)	sALRI	Defined population base	NPS; PCR	7.4	6.2	··	··	··	··	6.6	··
up_601	Mali (Sep 2011-Jan 2014) (Omer and colleagues)	sALRI	Defined population base	NPS; PCR	0	0	··	··	··	··	0	··

* NPA: nasopharyngeal aspirate. NPS: nasopharyngeal swab. NPW: nasopharyngeal wash. OPS: oropharyngeal swab. PCR: polymerase chain reaction. IFA: indirect immunofluorescence assay. DFA: direct fluorescent antibody. ELISA: enzyme-linked immunosorbent assay. HI: hemagglutination-inhibition assay. RIDT: rapid influenza diagnostic test. SPIA: solid-phase immunoassay. MN: micro-neutralization assay. TRFIA: time-resolved fluoroimmunoassay. ALRI, sALRI, and vsALRI: WHO definition for ALRI, chest wall indrawing ALRI, and very severe ALRI applied by a health worker. pSBI: WHO possible severe bacterial infections.

† Incidence rates were adjusted for the proportion of testing in eligible patients where available.

Supplementary table 23: Description of studies reporting incidence rates of IFV-associated very severe ALRI in children under 5 years (per 1,000 children per year) *†

ID	Location (reference)	Case Definition	Denominator source	Specimen and diagnostic test	0-27 d	1-2 m	3-5 m	6-11 m	12-23 m	24-59 m	0-5 m	0-59 m
up_1	Haryana, India (Aug 2012-Aug 2014) (Krishnan and colleagues)	vsALRI	Census-derived estimate	OP and nasal specimens; PCR	0	13.9	14.6	3.2	3.1	0.5	13.5	2.3
up_2	Managua, Nicaragua (Sep 2012-Sep 2015) (Gordon and colleagues)	vsALRI	Defined population base	Nasal and throat swabs; PCR	0	0	0	2.7	1.6	··	0	··
up_25	Kamalapur, Bangladesh (2007-2014) (Brooks and colleagues)	vsALRI	Defined population base	NPW; PCR and tissue culture	0	0	0	0.2	0.2	0	0	0.1
up_3	Western Province, South Africa (Mar 2012-Dec 2016) (Zar and colleagues)	vsALRI	Census-derived estimate	NPS; PCR	10.8	0	0	2.0	3.4	0	1.7	1.9

* NPA: nasopharyngeal aspirate. NPS: nasopharyngeal swab. NPW: nasopharyngeal wash. OPS: oropharyngeal swab. PCR: polymerase chain reaction. IFA: indirect immunofluorescence assay. DFA: direct fluorescent antibody. ELISA: enzyme-linked immunosorbent assay. HI: hemagglutination-inhibition assay. RIDT: rapid influenza diagnostic test. SPIA: Solid-phase immunoassay. MN: micro-neutralization assay. TRFIA: time-resolved fluoroimmunoassay. ALRI, sALRI, and vsALRI: WHO definition for ALRI, chest wall indrawing ALRI, and very severe ALRI applied by a health worker.

† Incidence rates were adjusted for the proportion of testing in eligible patients where available.

Supplementary table 24: Quality studies reporting incidence rates of IFV-diseases in children under five years by severity*†

Disease‡	ID	Location (Period of study)	Study design	Patient group excluded	Case definition	Sampling strategy	Diagnostic test
Episode	229	Vietnam;2007-2010	Low	Low	Low	Low	Low
Episode	700	Mali;2011-2014	Low	Low	High	Low	Low
Episode	701	United States of America;2009-2013	Low	Low	High	Low	Low
Episode	702	Japan;2009-2011	High	Low	High	Low	Low
Episode	703	Finland;2000-2002	Low	High	Low	Low	Low
Episode	801	Senegal;2012-2013	Low	Low	High	Low	Low
Episode	802	Senegal;2012-2013	Low	Low	High	Low	Low
Episode	b_3	Japan;2002-2008	Low	High	High	Low	High
Episode	b_40	United States of America;1974-1999	Low	Low	High	Low	Low
Episode	ba_1	Switzerland;1999-2004	Low	Low	Low	Low	Low
Episode	ba_3	India;2001-2005	Low	Low	High	Low	High
Episode	ba_4	Bangladesh;2004-2007	Low	Low	High	Low	Low
Episode	e17	United States of America;2010-2014	High	High	High	High	High
Episode	e19	India;2012-2014	Low	High	High	High	Low
Episode	e21	Australia;2011	Low	High	Low	Low	Low
Episode	e23	Nepal;2011-2013	Low	Low	High	Low	Low
Episode	e24	Southwest Finland;2010-2012	Low	Low	High	Low	Low
Episode	s408	Japan;2004-2008	Low	Low	Low	High	High
Episode	up_2	Nicaragua;2012-2015	Low	Low	High	Low	Low
Episode	up_25	Bangladesh;2007-2015	Low	Low	Low	Low	Low
Episode	up_600	Nepal;2011-2014	Low	Low	High	Low	Low
Episode	up_601	Mali;2011-2014	Low	Low	High	Low	Low
Episode	up_602	South Africa;2011-2013	Low	Low	High	Low	Low
Episode	1807	Australia; 2010-2014	Low	High	High	High	Low
Episode	f037	Romania; 2011-2016	High	Low	Low	Low	Low
Episode	f052	Nepal; 2011-2013	Low	Low	High	High	Low
Episode	f071	Spain; 2011-2016	Low	Low	High	High	Low
ALRI	703	Finland;2000-2002	Low	High	Low	Low	Low
ALRI	b_11	Germany;1999-2001	Low	Low	High	High	Low
ALRI	b_40	United States of America;1974-1999	Low	Low	Low	Low	Low
ALRI	ba_3	India;2001-2005	Low	Low	Low	Low	High
ALRI	ba_4	Bangladesh;2004-2007	Low	Low	Low	Low	Low
ALRI	e19	India;2012-2014	Low	High	Low	High	Low
ALRI	up_1	India;2012-2014	Low	Low	Low	Low	Low
ALRI	up_2	Nicaragua;2012-2015	Low	Low	Low	Low	Low
ALRI	up_25	Bangladesh;2007-2015	Low	Low	Low	Low	Low
ALRI	up_26	Pakistan;2012-2014	Low	Low	Low	Low	Low
ALRI	up_3	South Africa;2012-2016	Low	Low	Low	Low	Low

* Only in community-based studies
† Low: low risk of bias; high: high risk of bias
‡ Episode: for IFV-episode. sALRI: chest wall indrawing ALRI. vsALRI: for very severe ALRI.

Disease[‡]	ID	Location (Period of study)	Study design	Patient group excluded	Case definition	Sampling strategy	Diagnostic test
ALRI	1807	Australia; 2010-2014	Low	High	Low	High	Low
sALRI	ba_2	Bangladesh;1993-1996	Low	Low	Low	Low	High
sALRI	ba_4	Bangladesh;2004-2007	Low	Low	Low	Low	Low
sALRI	up_1	India;2012-2014	Low	Low	Low	Low	Low
sALRI	up_2	Nicaragua;2012-2015	Low	Low	Low	Low	Low
sALRI	up_25	Bangladesh;2007-2015	Low	Low	Low	Low	Low
sALRI	up_271	Pakistan;2011-2014	Low	Low	Low	Low	Low
sALRI	up_3	South Africa;2012-2016	Low	Low	Low	Low	Low
sALRI	up_521	Bangladesh;2011-2013	Low	Low	High	Low	Low
sALRI	up_522	Pakistan;2012-2013	Low	Low	High	Low	Low
sALRI	up_523	Pakistan;2012-2013	Low	Low	High	Low	Low
sALRI	up_524	India;2013-2014	Low	Low	High	Low	Low
sALRI	up_525	India;2013-2014	Low	Low	High	Low	Low
sALRI	up_600	Nepal;2011-2014	Low	Low	Low	Low	Low
sALRI	up_601	Mali;2011-2014	Low	Low	Low	Low	Low
vsALRI	up_1	India;2012-2014	Low	Low	Low	Low	Low
vsALRI	up_2	Nicaragua;2012-2015	Low	Low	Low	Low	Low
vsALRI	up_25	Bangladesh;2007-2015	Low	Low	Low	Low	Low
vsALRI	up_3	South Africa;2012-2016	Low	Low	Low	Low	Low

Supplementary table 25: Description of studies reporting IFV-associated ALRI hospitalisation rates in children under 5 years (per 1,000 children per year) [*†]

ID	Location (reference)	Case Definition	Denominator source	Specimen and diagnostic test	0-27 d	1-2 m	3-5 m	6-11 m	12-23 m	24-59 m	0-5 m	0-59 m
141	Baguio City, Philippines; urban (2009-2011) (Tallo et al, 2014)[33]	ALRI AND Fever	Census-derived estimate	OPS or NPS; PCR	··	··	··	··	··	2.4	4.4	3.9
156	Jingzhou, China; urban (Apr 2010-Mar 2012) (Yu et al, 2014)[34]	ARI AND Fever	Census-derived estimate	NPS; PCR	··	··	··	37	23.6	21.4	25.1	21.9
32	Multistate, USA (Oct 2003-Apr 2012) (Chaves et al, 2014)[35]	Flu	Census-derived estimate	NPS or OPS; PCR, culture, DFA, IDFA, or RIDT	··	··	1.0	0.9	··	··	2	··
34	Hong Kong (2004-2011) (Chiu et al, 2014)[36]	ARI AND Fever	Census-derived estimate	NPA; DFA and viral culture, PCR	··	··	··	7.6	7.8	9.7	8.4	9.0
80	Hong Kong (Apr 2005-Mar 2011) (Nelson et al, 2014)[37]	All	Defined population base	NPA; Viral culture and IF	··	··	··	13.5	11.8	9.0	12.8	10.3
b_10	Bondo district, Kenya (Jun 2007-May 2009) (from Nair et al, 2011)[10]	ALRI	Census-derived estimate	NPS or OPS; PCR	··	··	··	··	1.3	0.3	··	··
b_11	Multicentric, Germany (Nov 1999- Oct 2001) (Forster et al, 2004)[31]	ARI	Census-derived estimate	NPA; PCR	··	··	··	··	1.4	··	··	··
b_13	Multistate, USA (Oct-April 2003-2008) (Dawood et al, 2010)[38]	Flu	Census-derived estimate	NPS or OPS; PCR, culture, DFA/IDFA, or RIDT	··	··	··	··	0.5	0.2	··	0.4
b_14	Kiel, Germany (Jul 1996-Jun 2000) (Wiegl et al, 2005)[39]	ARI	Census-derived estimate	NPA; RT-PCR	··	··	··	··	··	··	··	1.2
b_15	Gipuzoka, Spain (Jul 2001-Jun 2004) (Montes et al, 2005)[40]	ARI	Census-derived estimate	NPA; Viral culture and PCR	··	··	··	0.8	0.7	0.5	4.1	0.9
b_16	East London, United Kingdom (Oct 2002- Sep 2004) (Ajayi-Obe et al, 2008)[41]	ARI	Census-derived estimate	NPA; IFA and PCR	··	··	··	··	··	0.7	4.3	1.6
b_17	Leicester, United Kingdom (Oct 2001- Jun 2002) (Nicholson et al, 2006)[42]	ARI	NHS data base	Nasal and throat swabs; PCR	··	··	··	··	2.0	··	··	1.6

[*] NPA: nasopharyngeal aspirate. NPS: nasopharyngeal swab. NPW: nasopharyngeal wash. OPS: oropharyngeal swab. BAL: bronchoalveolar lavage. ETA: endotracheal aspirate. PCR: polymerase chain reaction. IFA: indirect immunofluorescence assay. DFA: direct fluorescent antibody. ELISA: enzyme-linked immunosorbent assay. IF: immunofluorescence. HI: hemagglutination-inhibition assay. RIDT: rapid influenza diagnostic test. SPIA: Solid-phase immunoassay. EIA: enzyme immunoassay. MN: micro-neutralization assay. TRFIA: time-resolved fluoroimmunoassay. ARI: any respiratory infections/symptoms (acute one of cough, sore throat, shortness of breath, coryza); ICD-codes for influenza & pneumonia (excluding non-respiratory manifestations), or acute infections (fever or <35 deg C) with any respiratory signs. ALRI: physician-diagnosed pneumonia and/or bronchiolitis, or WHO definition for ALRI requiring hospitalisation. All: all diagnosis related to influenza (including influenza & pneumonia and other non-respiratory illnesses). Flu: evidence of a positive influenza test.

[†] Hospitalisation rates were adjusted for the proportion of testing in hospitalised ALRI patients where available.

ID	Location (reference)	Case Definition	Denominator source	Specimen and diagnostic test	0-27 d	1-2 m	3-5 m	6-11 m	12-23 m	24-59 m	0-5 m	0-59 m
b_19	Nha Trang, Vietnam (Mar 2007- Feb 2008) (Yoshida et al, 2010)[43]	ARI	Census-derived estimate	NPA; PCR	··	··	··	··	18.4	4.2	··	8.7
b_20	Hong Kong (Jul 1997- Jun 1999) (Nelson et al, 2007)[44]	ARI	Census-derived estimate	NPA; Viral culture and serology	··	··	··	··	4.4	2.2	··	3
b_21	Hong Kong (Oct 2003- Sep 2006) (Chiu et al, 2009)[45]	ARI AND Fever	Census-derived estimate	NPA; DFA and viral culture	··	··	··	··	7.3	7.2	··	7.2
b_22	Suzhou, China (Jan 2007- Dec 2008) (Ji et al, 2010)[46]	ALRI	Census-derived estimate	NPA; DFA	··	··	··	··	0.1	0.2	··	0.2
b_24	Nashville, Rochester; Cincinati, USA (Oct 2001-Sep 2004) (Poehling et al, 2006)[47]	ARI; Fever	Census-derived estimate	Nasal and throat swabs; Viral Culture and PCR	··	··	··	··	··	0.3	4.5	1
b_25	Monroe County NY, and Davidson County TN, USA (Oct 2000 – Sep 2001) (Iwane et al, 2004)[48]	ARI	Census-derived estimate	Nasal and throat swabs; Viral Culture and PCR	··	··	··	1	0.5	0.2	2.4	0.6
b_26	Salt Lake County, Utah, USA (Jul 2001-Jun 2004) (Ampofo et al, 2006)[49]	ARI	Census-derived estimate	NPA; DFA	··	··	··	3.3	2.9	1	7.4	2.6
b_27	Philadelphia, USA (Jul 2000-Jun 2004) (Coffin et al, 2007)[50]	All	Census-derived estimate	Nasal aspirates; SPIA, DFA and viral culture	··	··	··	··	··	0.7	··	2.1
b_28	Davidson County, USA (2003-2004) (Grijalva et al, 2006)[51]	ARI; Fever	Census-derived estimate	Nasal and throat swabs; Viral culture, PCR, RIDT, IFA, serology	··	··	··	··	··	0.3	5.4	1.2
b_3	Soma and Shinchi, Japan (2002-2008) (from Nair et al, 2011)[10]	ARI AND Fever	Defined population base	Nasal swabs; RIDT	··	··	··	33.7	37.3	24.3	··	··
b_40	Nashville, TN, USA (Aug 1974-Jul 1999) (Neuzil et al, 2002)[15]	ARI; Fever	Defined population base	Nasal wash; Culture and HI or DFA	··	··	··	··	3.5	0	··	2.3
b_9	Manhiça, Mozambique (Sep 2006-Sep 2007) (from Nair et al, 2011)[10]	ALRI	Defined population base	NPA; PCR	··	··	··	3	3.4	0.3	4.5	1.7
e13	Baguio City, Philippines (Jan 2012-Dec 2014) (Kamigaki et al, 2017)[52]	ALRI	Census derived estimate	OPS or NPS; PCR	··	··	··	··	··	3.6	8.7	7.2
e26	Australia (2006-2015) (Li-Kim-Moy et al, 2016)[53]	ARI	Census derived estimate	··; ··	··	··	··	··	··	0.4	1.9	0.8
s103	Memphis, Nashville, and Salt Lake City; urban (Jan 2010-Jun 2012) (Jain et al, 2015)[54]	ALRI	Census-derived estimate	NPS and OPS; PCR, serology	··	··	··	··	··	0.1	··	0.2
s138	Athens, Greece (2002-2003; 2004-2005) (Sakkou et al, 2011)[55]	ARI; Fever	Census-derived estimate	NPA; PCR	··	··	··	··	··	··	··	··

ID	Location (reference)	Case Definition	Denominator source	Specimen and diagnostic test	0-27 d	1-2 m	3-5 m	6-11 m	12-23 m	24-59 m	0-5 m	0-59 m
s140	Turku, Finland; (Jul 1988-Jun 2004) (Silvennoinen et al, 2011)[56]	All	Defined population base	NPA; IFA and RIDT	··	··	··	··	··	··	2.8	··
up_10	St. Elizabeth, Lwak, Asembo, Kenya; rural (2010-2014) (Chaves and colleagues)	ALRI	Census-derived estimate	NPS and OPS; PCR	0	0	0	13.1	7.2	0.9	0	3.3
up_11	Siaya County, Kenya; rural (2010-2014) (Chaves and colleagues)	ALRI	Census-derived estimate	NPS and OPS; PCR	0	1.7	2.3	2.1	1.9	0.6	1.8	1.1
up_13	Soweto, Gauteng, South Africa; urban (Mar 1998 to Oct 2005) (Madhi and colleagues)	ALRI	Defined population base	NPA; IFA	··	3.4	4.3	2.1	1.8	0.3	4	1.1
up_14	Amman, Jordan; urban (Mar 2010-Mar 2013) (Khuri-Bulos and colleagues)	ALRI	Census-derived estimate	Nasal or throat swabs; PCR	··	··	··	··	0.2	··	··	··
up_15	Manhiça, Mozambique; rural (Jan 2011-June 2014) (Bassat and colleagues)	ALRI	Census-derived estimate	NPA; PCR	1.5	0.7	1.2	1.5	0.6	0.2	1.1	0.5
up_16	Kilifi, Kenya; rural and semi-urban (Jan 2007- Dec 2016) (Nokes and colleagues)	ALRI	Census-derived estimate	NPS; PCR	0.4	2.2	2.1	1.8	1.0	0.4	1.7	0.8
up_17	Muang District, Nakhon Phanom Province, Thailand (PERCH); rural (2012-2013) (O'Brien and colleagues)	ALRI	Census-derived estimate	NP/OP and induced sputum; PCR	··	··	··	··	0	0.1	··	0.1
up_18	Muang District, Sa Kaeo Province, Thailand; rural (2012-2013) (O'Brien and colleagues)	ALRI	Census-derived estimate	NP/OP and induced sputum; PCR	··	··	··	··	0.4	0.2	··	0.2
up_19	Nha Trang city, Vietnam; urban and sub-urban (2008-2013) (Yoshida and colleagues)	ALRI	Census-derived estimate	NP specimens; PCR	··	··	··	··	1.4	0.2	··	0.7
up_21	Basse, Upper River Region, The Gambia (PERCH); rural (Nov 2011-Nov 2013) (O'Brien and colleagues)	ALRI	Census-derived estimate	NPS, OPS, induced sputum; PCR	··	4.5	2.6	0.6	0.2	0.3	3.3	0.6
up_22	Bersheba, Israel (Sep-Mar 2011-2016) (Katz and colleagues)	ARI; Fever	Medical Records	OP and nasal swabs; PCR	9.6	15.3	8.9	12.6	6	3.5	11.5	5.3
up_23	Nakhon Phanom and Sa Kaeo Provinces, Thailand; rural (Jan 2005-Dec 2011) (Thamtithiwat and colleagues)	ARI	Census-derived estimate	NPS; PCR	0.6	2	4.2	9.1	9	4.4	2.9	5.6
up_24	Pune district, India; rural (May 2009-Apr 2013) (Hirve and colleagues)	ALRI	Census-derived estimate	NPS; PCR	0	0	0	1.2	0	0.1	0	0.2

ID	Location (reference)	Case Definition	Denominator source	Specimen and diagnostic test	0-27 d	1-2 m	3-5 m	6-11 m	12-23 m	24-59 m	0-5 m	0-59 m
up_25	Kamalapur, Bangladesh (2007-2014) (Brooks and colleagues)	ALRI	Defined population base	NPW; PCR and tissue culture	0	0.2	0.3	0.8	2.5	0.4	0.2	0.7
up_26	David City, Panama (2014-2016) (Jara and colleagues)	ALRI	Census-derived estimate	NPS or OPS; PCR	··	··	··	··	8.6	1.2	··	4.9
up_27	Ciudad de Buenos Aires, Argentina; urban (Jun 2008-Dec 2010) (Echavarria and colleagues)	ALRI	Defined population base	NPA; IFA	··	··	··	··	4.4	0.6	··	4
up_28	Turku, Finland; urban (Jan 2010-Jun 2012) (Heikkinen and colleagues)	ALRI	Census-derived estimate	Nasal swabs; TRFIA	1.1	4.9	0.3	0.7	0.7	0.1	2	0.5
up_29	Aurora, Colorado, USA; urban (Jan 2011-Oct 2015) (Simões and colleagues)	ALRI	Census-derived estimate	··; ··	··	··	··	··	0.5	0.2	··	0.3
up_3	Paarl, Western Province, South Africa (Jun 2012-Dec 2016) (Zar and colleagues)	ALRI	Defined population base	NPS; PCR	21.1	28.0	7.2	7.9	1.1	1.1	17.1	5.6
up_30	Region VI Buenos Aires Province, Argentina; urban /slums/semi-rural (2011-2013) (Polack and colleagues)	ALRI	Census-derived estimate	NPA; PCR	0	2.0	4.3	2.1	1.4	··	2.9	··
up_31	Quetzaltnango, Guatemala (2010-2016) (McCracken and colleagues)	ALRI	Census-derived estimate	NPS and OPS; PCR	0.8	2.4	1.3	0.9	0.6	0.1	1.6	0.5
up_32	Santa Rosa, Guatemala (2010-2016) (McCracken and colleagues)	ALRI	Census-derived estimate	NPS and OPS; PCR	2.8	2.9	1.4	0.7	1.3	0.1	2.1	0.6
up_33	Tagbilaran, Bohol, Philipines; Dauis, Baclayon, Panglao, Cortes, Balilihan, Bohol, Philippines; mixed urban-rural (July 2000-Dec 2004) (Lucero and colleagues)	ALRI	Defined population base	NPA; Viral culture	··	0.8	3.0	3.0	1.3	··	2.4	··
up_34	Valencia Region, Spain (2014-2017) (Mira Iglesias and colleagues)	All	Census-derived estimate	NPS and nasal swabs; PCR	··	··	··	··	1.3	0.3	··	0.9
up_4	Buenos Aires, Argentina; urban (2009-2016) (Gentile and colleagues)	ALRI	Census-derived estimate	NPA; PCR	··	··	··	··	3.4	1.1	··	2.6
up_41	Soweto, Gauteng, South Africa (2015-2017) (Madhi and colleagues)	ALRI	Census-derived estimate	NPS; PCR	··	··	··	··	1.8	0.4	··	2.3
up_5	Klerksdorp, North West Province, South Africa; peri-urban (2013-2015) (Cohen and colleagues)	ALRI; Sepsis	Census-derived estimate	NPA; PCR	0	1.6	1.6	1.8	1.4	0.3	1.3	0.8

ID	Location (reference)	Case Definition	Denominator source	Specimen and diagnostic test	0-27 d	1-2 m	3-5 m	6-11 m	12-23 m	24-59 m	0-5 m	0-59 m
up_6	Pietermaritzburg, Kwa-Zulu Natal Province, South Africa; peri-urban (2013-2015) (Cohen and colleagues)	ALRI; Sepsis	Census-derived estimate	NPA; PCR	0.9	1.3	1.5	2.0	0.7	0.1	1.3	0.5
up_7	Soweto, Gauteng, South Africa; urban (2009-2012) (Cohen and colleagues)	ALRI; Sepsis	Census-derived estimate	NPA; PCR	1.7	1.7	1.4	1.4	0.7	0.1	1.6	0.5
up_8	Concepcion, Chile; mixed urban/rural (2012-2013) (Fasce and colleagues)	ALRI	Census-derived estimate	NPA or NPS; PCR	··	··	··	··	0.4	0.2	··	0.4
up_9	Iquique, Chile; mixed urban-rural (2012-2013) (Fasce and colleagues)	ALRI	Census-derived estimate	NPA; PCR	··	··	··	··	0.4	0.4	··	0.7
111	New Haven County, CT, USA (Oct 2003-April 2010) (Yousey-Hindes et al, 2011)[57]	Flu	Census-derived estimate	NPS or OPS; Viral culture, DFA or IDFA, PCR, and RIDT	··	··	··	··	··	··	··	1
116	Kishoreganj, Bogra, Comilla and Barisal, Bangladesh (2010-2014) (Homaira et al, 2016)[58]	ARI	Census-derived estimate	Nasal and throat swabs; PCR	··	··	··	··	··	··	··	0.4
4001	Sohar, Oman (2008-2013) (Al-Awaidy et al, 2015)[59]	ALRI	Census-derived estimate	OP and NP swab; PCR	··	··	··	··	··	··	··	0.3
4002	Ibra, Oman (2008-2013) (Al-Awaidy et al, 2015)[59]	ARI	Census-derived estimate	OP and NP swab; PCR	··	··	··	··	··	··	··	0.4
4003	SQH, Oman (2008-2013) (Al-Awaidy et al, 2015)[59]	ARI	Census-derived estimate	OP and NP swab; PCR	··	··	··	··	··	··	··	0.4
422	El Salvador (2009-2012) (Descalzo et al, 2016)[60]	All	Census-derived estimate	Nasal and pharyngeal specimens; PCR and IFA	··	··	··	··	··	··	··	1.8
423	Guatemala (2009-2012) (Descalzo et al, 2016)[60]	All	Census-derived estimate	Nasal and pharyngeal specimens; PCR and IFA	··	··	··	··	··	··	··	0.7
424	Honduras (2009-2012) (Descalzo et al, 2016)[60]	All	Census-derived estimate	Nasal and pharyngeal specimens; PCR and IFA	··	··	··	··	··	··	··	0.9
425	Nicaragua (2009-2012) (Descalzo et al, 2016)[60]	All	Census-derived estimate	Nasal and pharyngeal specimens; PCR and IFA	··	··	··	··	··	··	··	4.5
b_18	Madrid, Spain (1997-2003) (Rojo et al, 2006)[61]	ARI; Fever	Census-derived estimate	Nasal or throat aspirates; Viral culture and subsequent fluorescent staining	··	··	··	··	··	··	··	··
b_23	South Australia, Australia (1996-2006) (D'Onise et al, 2008)[62]	ARI	Census-derived estimate	··; Viral Culture, PCR	··	··	··	··	··	··	··	0.6
b_30	Milwaukee, Wisconsin, USA (Nov 1996- Oct 1998) (Henrickson et al, 2004)[63]	ARI; Fever	Census-derived estimate	NPS, BAL, throat swabs, ETA; PCR, Tissue culture, EIA	··	··	··	··	··	··	··	1.5
b_31	Rio de Janeiro, Brazil (1987-1989) (Sutmoller et al, 1995)[64]	ALRI	Defined population base	NPA; IFA, viral culture	··	··	··	··	··	··	··	2.5
b_8	Santa Rosa, Guatemala (2008) (from Nair et al, 2011)[10]	ARI	Census-derived estimate	NPS or OPS; PCR	··	··	··	··	··	··	··	0.7

ID	Location (reference)	Case Definition	Denominator source	Specimen and diagnostic test	0-27 d	1-2 m	3-5 m	6-11 m	12-23 m	24-59 m	0-5 m	0-59 m
e14	Ghana (May 2013-April 2015) (Ntiri et al, 2016)[65]	ARI AND Fever	Census derived estimate	NPS, OPS; PCR	··	··	··	··	··	··	··	3
e16	England, United Kingdom (2010-2015) (Boddington et al, 2017)[66]	ARI AND Fever	Census derived estimate	··; PCR	··	··	··	··	··	··	··	0.3
e25	Germany (Jan 2005-Dec 2012) (Von Der Beck et al, 2017)[67]	ARI	Census derived estimate	··; ··	··	··	··	··	··	··	··	0.3
s134	Hamilton County, Ohio (Cincinnati); Monroe County, New York (Rochester); and Davidson County, Tennessee (Nashville) (2004-2009) (Poehling et al, 2013)[68]	ARI; Fever	Census-derived estimate	Nasal and throat swabs; PCR	··	··	··	··	··	··	··	0.6
s205	Scotland, United Kingdom (Nov 2012-Apr 2013) (Harvala et al, 2014)[69]	ALRI	Census-derived estimate	Nose and throat swabs; PCR	··	··	··	··	··	··	··	2.8
s207	Leganes, Madrid, Spain (Oct 2011-Dec 2012) (Olabarrieta et al, 2015)[70]	ARI	Defined population base	NPA; PCR	··	··	··	··	··	··	··	··
s304	Multistate, USA (Oct 2010-Apr 2011) (Chaves et al, 2013)[71]	Flu	Census-derived estimate	NPS or OPS; Culture, DFA, IFA, RIDT, or PCR	··	··	··	··	··	··	··	0.5
up_12	Tone and Cinkasse districts, Togo; mixed urban-rural (August 2011-Oct 2013 and Aug 2014-July 2015) (Moïsi and colleagues)	ALRI	Census-derived estimate	NPA; PCR	··	··	··	··	··	··	··	0.1
up_602	Soweto, South Africa (March 2011-May 2013) (Omer and colleagues)	ALRI	Defined population base	NPS, PCR	0	5.6	··	··	··	··	3.9	··
f008	Kinshasa Province, Congo, Dem. Rep (2013-2015) (Babakazo et al, 2018) [72]	ALRI	Census-derived estimate	OPS and NPS; PCR	··	··	··	··	··	··	··	2.3
f047A	Svay Rieng, Cambodia (2015) (Ieng et al, 2018) [73]	ALRI-Fever	Census-derived estimate	NPS; PCR	··	··	··	··	··	··	··	0.1
f047B	Siem Reap, Cambodia (2016) (Ieng et al, 2018) [73]	ALRI-Fever	Census-derived estimate	NPS; PCR	··	··	··	··	··	··	··	2.6
f047C	Kampong Cham, Cambodia (2016) (Ieng et al, 2018) [73]	ALRI-Fever	Census-derived estimate	NPS; PCR	··	··	··	··	··	··	··	3.9
f070	Rwanda (2012-2014) (Nyamusore et al, 2018) [74]	ARI-Fever	Census-derived estimate	NPS and OPS; PCR	··	··	··	··	··	··	··	1.7
f071	Spain (2010-2016) (Oliva et al, 2018) [30]	ALRI	Census-derived estimate	Respiratory swab; ··	··	··	··	··	··	··	··	0.2
f087	Chile (2012-2014) (Sotomayor et al, 2018) [75]	ARI-Fever	Census-derived estimate	NPA and NPS; PCR and IF	··	··	··	··	··	··	··	0.7
f090A	Deli Serdang, Indonesia (2013-2016) (Susilarini et al, 2018) [76]	ARI-Fever	Census-derived estimate	Respiratory specimen; PCR	··	··	··	··	··	··	··	1.5

ID	Location (reference)	Case Definition	Denominator source	Specimen and diagnostic test	0-27 d	1-2 m	3-5 m	6-11 m	12-23 m	24-59 m	0-5 m	0-59 m
f090B	Balikpapan, Indonesia (2013-2016) (Susilarini et al, 2018) [76]	ARI-Fever	Census-derived estimate	Respiratory specimen; PCR	··	··	··	··	··	··	··	6.1
f090C	Gunung Kidul, Indonesia (2013-2016) (Susilarini et al, 2018) [76]	ARI-Fever	Census-derived estimate	Respiratory specimen; PCR	··	··	··	··	··	··	··	2.5
f118	Beijing, China (2014-2016) (Zhang et al, 2018) [77]	ARI-Fever	Census-derived estimate	Throat swab; PCR	··	··	··	··	··	··	··	4.3
f207	Beijing, China (2017-2018) (Zhao et al, 2018) [78]	ARI-Fever	Census-derived estimate	Throat swab; PCR	··	··	··	··	··	5.3	··	5.0
f231	Oman (2012-2015) (Doaa M. Abdel-Hady et al, 2018) [79]	ARI	Census-derived estimate	Respiratory specimen; PCR	··	··	··	··	··	··	··	0.8

Supplementary table 26: Description of studies reporting hospitalisation rates of IFV-associated ALRI with hypoxemia in children under 5 years (per 1,000 children per year) *†

ID	Location (reference)	Case Definition	Denominator source	Specimen and diagnostic test	0-27 d	1-2 m	3-5 m	6-11 m	12-23 m	24-59 m	0-5 m	0-59 m
up_10	St. Elizabeth, Lwak, Asembo, Kenya; Rural (2010-2014) (Chaves and colleagues)	sALRI	Census-derived estimate	NPS and OPS; PCR	0	0	0	7.8	2.4	0	0	1.3
up_11	Siaya County, Kenya; Rural (2010-2014) (Chaves and colleagues)	sALRI	Census-derived estimate	NPS and OPS; PCR	0	1.2	1.5	1.5	1.4	0.6	1.2	0.9
up_13	Soweto, Gauteng, South Africa; urban (Mar 1998-Oct 2005) (Madhi and colleagues)	sALRI	Defined population base	NPA; IFA	··	0.8	1.3	0.7	0.5	0.1	1.1	0.3
up_14	Amman, Jordan; urban (Mar 2010-Mar 2013) (Khuri-Bulos and colleagues)	sALRI	Census-derived estimate	Nasal or throat swabs; PCR	··	··	··	··	0	··	··	··
up_15	Manhiça, Mozambique; rural (Jan 2011-Jun 2014) (Bassat and colleagues)	sALRI	Census-derived estimate	NPA; PCR	0	0	0	0.6	0.2	0	0	0.1
up_16	Kilifi, Kenya; rural and semi-urban (Jan 2007-Dec 2016) (Nokes and colleagues)	sALRI	Census-derived estimate	NPS; PCR	0.1	0.4	0.6	0.3	0.2	0.1	0.4	0.1
up_19	Nha Trang city, Vietnam; urban and sub-urban (2008-2013) (Yoshida and colleagues)	sALRI	Census-derived estimate	NP specimens; PCR	··	··	··	··	0.2	0.1	··	0.2
up_24	Pune district, India; rural (May 2009 - Apr 2013) (Hirve and colleagues)	sALRI	Census-derived estimate	NPS; PCR	0	0	0	0	0.3	0	0	··
up_27	Ciudad de Buenos Aires, Argentina; urban (Jun 2008-Dec 2010) (Echavarria and colleagues)	sALRI	Defined population base	NPA; IFA	··	··	··	··	1.1	0	··	0.3
up_29	Aurora, Colorado, United States; urban (Jan 2011-Oct 2015) (Simões and colleagues)	sALRI	Census-derived estimate	··	··	··	··	··	0.3	0.1	··	0.2

* NPA: nasopharyngeal aspirate. NPS: nasopharyngeal swab. NPW: nasopharyngeal wash. OPS: oropharyngeal swab. PCR: polymerase chain reaction. IF: immunofluorescence. IFA: indirect immunofluorescence assay. DFA: direct fluorescent antibody. ELISA: enzyme-linked immunosorbent assay. HI: hemagglutination-inhibition assay. RIDT: rapid influenza diagnostic test. MN: micro-neutralization assay. TRFIA: time-resolved fluoroimmunoassay. sALRI: hospitalised ALRI with hypoxemia, or ALRI in ICU or requiring MV. All: all diagnosis related to influenza (including influenza & pneumonia and other non-respiratory illnesses). Flu: evidence of a positive influenza test.

† Hospitalisation rates were adjusted for the proportion of testing in hospitalised ALRI patients where available.

ID	Location (reference)	Case Definition	Denominator source	Specimen and diagnostic test	0-27 d	1-2 m	3-5 m	6-11 m	12-23 m	24-59 m	0-5 m	0-59 m
up_30	Region VI Buenos Aires Province, Argentina; urban /slums/semi-rural (2011-2013) (Polack and colleagues)	sALRI AND SpO2 <93%	Census-derived estimate	NPA; PCR	0	0.7	0.9	1.3	0.6	··	0.7	··
up_31	Quetzaltnango, Guatemala (2010-2016) (McCracken and colleagues)	sALRI	Census-derived estimate	NPS and OPS; PCR	0.8	2.4	1.2	0.8	0.6	0.1	1.5	0.4
up_32	Cuilapa, Santa Rosa, Guatemala (2010-2016) (McCracken and colleagues)	sALRI	Census-derived estimate	NPS and OPS; PCR	1.8	2.9	1.2	0.6	0.8	0.1	1.8	0.5
up_33	Tagbilaran City, Bohol, Philipines; Dauis, Baclayon, Panglao, Cortes, Balilihan, Bohol, Philippines; mixed urban-rural (Jul 2000-Dec 2004) (Lucero and colleagues)	sALRI	Defined population base	NPA; Viral culture	··	0	0.9	0	0.1	··	0.7	··
up_34	Valencia Region, Spain (2014-2017) (Mira Iglesias and colleagues)	All	Census-derived estimate	NPS and nasal swabs; PCR	··	··	··	··	0	0	··	0.0
up_4	Buenos Aires, Argentina; urban (2009-2016) (Gentile and colleagues)	sALRI	NA	NPA; PCR	··	··	··	··	3.2	1.1	··	2.5
up_12	Tone and Cinkasse districts, Togo; mixed urban-rural (Aug 2011-Oct 2013; Aug 2014-Jul 2015) (Moïsi and colleagues)	sALRI	Census-derived estimate	NPA; PCR	··	··	··	··	··	··	··	0

Supplementary table 27: Description of studies reporting hospitalisation rates of IFV-associated very severe ALRI in children under five years (per 1,000 children per year) *†

ID	Location (reference)	Case Definition	Denominator source	Specimen and diagnostic test	0-27 d	1-2 m	3-5 m	6-11 m	12-23 m	24-59 m	0-5 m	0-59 m
146	France (2009-2013) (Bonmarin et al, 2015)[80]	Flu AND ICU	Census-derived estimate	··	··	··	··	··	··	··	··	0
66	Australia (1997-2013) (Kaczmarek et al, 2016)[81]	Flu AND ICU	Census-derived estimate	··	··	··	··	··	··	··	··	0
up_10	St. Elizabeth, Lwak, Asembo, Kenya; rural (2010-2014) (Chaves and colleagues)	ALRI AND ICU; MV; danger signs	Census-derived estimate	NPS and OPS; PCR	0	0	0	5.2	2.4	0	0	1.0
up_11	Siaya County, Kenya; rural (2010-2014) (Chaves and colleagues)	ALRI AND ICU; MV; danger signs	Census-derived estimate	NPS and OPS; PCR	0	0.6	1.5	1.3	1.4	0.5	1	0.8
up_13	Soweto, Gauteng, South Africa; urban (Mar 1998-Oct 2005) (Madhi and colleagues)	ALRI AND ICU; MV; danger signs	Defined population base	NPA; IFA	··	0	0	0	0	0	0	0
up_14	Amman, Jordan; urban (Mar 2010-Mar 2013) (Khuri-Bulos and colleagues)	ALRI AND ICU; MV; danger signs	Defined population base	Nasal/throat swabs; PCR	··	··	··	··	0	··	··	··
up_15	Manhiça, Mozambique; rural (Jan 2011-Jun 2014) (Bassat and colleagues)	ALRI AND ICU; MV; danger signs	Census-derived estimate	NPA; PCR	0	0	0	0.6	0.2	0	0	0.1
up_16	Kilifi, Kenya; rural and semi-urban (Jan 2007- Dec 2016) (Nokes and colleagues)	ALRI AND ICU; MV; danger signs	Census-derived estimate	NPS; PCR	0.2	0.9	1.1	0.4	0.3	0.1	0.8	0.3
up_17	Muang District, Nakhon Phanom Province, Thailand (PERCH); rural (2012-2013) (O'Brien and colleagues)	ALRI AND chest wall indrawing AND danger signs	Census-derived estimate	NP/OP and induced sputum; PCR	··	··	··	··	0	0.1	··	0.1
up_18	Muang District, Sa Kaeo Province, Thailand; rural (2012-2013) (O'Brien and colleagues)	ALRI AND chest wall indrawing AND danger signs	Census-derived estimate	NP/OP and induced sputum; PCR	··	··	··	··	0.4	0.1	··	0.1
up_19	Nha Trang city, Vietnam; urban and sub-urban (2008-2013) (Yoshida and colleagues)	ALRI AND ICU; MV; danger signs	Census-derived estimate	NP specimens; PCR	··	··	··	··	0	0	··	0

* NPA: nasopharyngeal aspirate. NPS: nasopharyngeal swab. NPW: nasopharyngeal wash. OPS: oropharyngeal swab. PCR: polymerase chain reaction. RT-PCR: reverse transcriptase polymerase chain reaction. IFA: indirect immunofluorescence assay. DFA: direct fluorescent antibody. ELISA: enzyme-linked immunosorbent assay. IF: immunofluorescence. HI: hemagglutination-inhibition assay. RIDT: rapid influenza diagnostic test. SPIA: Solid-phase immunoassay. EIA: enzyme immunoassay. MN: micro-neutralization assay. TRFIA: time-resolved fluoroimmunoassay. ICU: intensive care unit. MV: mechanical ventilation. All: all diagnosis related to influenza (including influenza & pneumonia and other non-respiratory illnesses). Flu: laboratory-confirmed influenza.

† Hospitalisation rates were adjusted for the proportion of testing in hospitalised ALRI patients.

ID	Location (reference)	Case Definition	Denominator source	Specimen and diagnostic test	0-27 d	1-2 m	3-5 m	6-11 m	12-23 m	24-59 m	0-5 m	0-59 m
up_21	Basse, Upper River Region, The Gambia (PERCH); rural (Nov 2011-Nov 2013) (O'Brien and colleagues)	ALRI AND chest wall indrawing AND danger signs	Census-derived estimate	NPS/OPS, induced sputum; PCR	··	0	0	0.1	0	0.1	0	0
up_22	Bersheba, Israel (Sep-Mar 2011-2016) (Katz and colleagues)	ARI; Fever AND ICU	Clalit Health Services Electronic Medical Records	OPS and nasal swabs; PCR	0	0.2	0.4	0.1	0	0	0.3	0.1
up_23	Nakhon Phanom and Sa Kaeo Provinces, Thailand; rural (Jan 2005-Dec 2011) (Thamtithiwat and colleagues)	Intubation or SpO2 <80 mmHg	Census-derived estimate	NPS; PCR	0	0.2	0.1	0.7	0.5	0.1	0.2	0.3
up_26	David City, Panama (2014-2016) (Jara and colleagues)	ALRI; ICU/MV/with danger signs	Census-derived estimate	NPS or OPS; PCR	··	··	··	··	0.4	0	··	0.6
up_27	Ciudad de Buenos Aires, Argentina; urban (Jun 2008-Dec 2010) (Echavarria and colleagues)	ALRI AND ICU; MV; danger signs	Defined population base	NPA; IFA	··	··	··	··	0	0	··	0
up_28	Turku, Finland; urban (Jan 2010-Jun 2012) (Heikkinen and colleagues)	ALRI AND ICU; MV; danger signs	Census-derived estimate	Nasal swabs; TRFIA	0	1	0	0	0.2	0	0.3	0.1
up_29	Aurora, Colorado, USA; urban (Jan 2011-Oct 2015) (Simões and colleagues)	ALRI AND ICU; MV; danger signs	Census-derived estimate	··	··	··	··	··	0	0	··	0
up_30	Region VI Buenos Aires Province, Argentina; urban /slums/semi-rural (2011-2013) (Polack and colleagues)	ALRI AND ICU; MV; danger signs	Census-derived estimate	NPA; PCR	0	0	0.6	0.4	0	··	0.3	··
up_31	Quetzaltnango, Guatemala (2010-2016) (McCracken and colleagues)	ALRI; ICU/MV/with danger signs	Census-derived estimate	NPS and OPS; PCR	0.8	2.4	1.1	0.8	0.5	0.1	1.5	0.4
up_32	Cuilapa, Santa Rosa, Guatemala (2010-2016) (McCracken and colleagues)	ALRI; ICU/MV/with danger signs	Census-derived estimate	NPS and OPS; PCR	1.8	2.9	1.2	0.6	0.8	0.1	1.8	0.5
up_33	Tagbilaran City, Bohol, Philipines; Dauis, Baclayon, Panglao, Cortes, Balilihan, Bohol, Philippines; mixed urban-rural (Jul 2000-Dec 2004) (Lucero and colleagues)	ALRI; ICU/MV/with danger signs	Defined population base	NPA; Viral culture	··	0	0.9	0	0	··	0.7	··
up_34	Valencia Region, Spain (2014-2017) (Mira Iglesias and colleagues)	All AND ICU; MV; danger signs	Census-derived estimate	NPS and nasal swabs; PCR	··	··	··	··	0	0	··	0

ID	Location (reference)	Case Definition	Denominator source	Specimen and diagnostic test	0-27 d	1-2 m	3-5 m	6-11 m	12-23 m	24-59 m	0-5 m	0-59 m
up_4	Buenos Aires, Argentina; urban (2009-2016) (Gentile and colleagues)	ALRI AND ICU; MV; danger signs	Census-derived estimate	NPA; PCR	··	··	··	··	0.6	0.2	··	0.4
up_8	Concepcion, Chile; mixed urban/rural (2012-2013) (Fasce and colleagues)	ALRI AND ICU; MV; danger signs	Census-derived estimate	NPA, NPS; PCR	··	··	··	··	0.1	0	··	0.1
up_9	Iquique, Chile; mixed urban-rural (2012-2013) (Fasce and colleagues)	ALRI AND ICU; MV; danger signs	Census-derived estimate	NPA; PCR	··	··	··	··	0	0.1	··	0.1
118	Denmark (2009-10; 2010-11) (Gubbels et al, 2013)[82]	Flu AND ICU	Sentinel surveillance	··; PCR	··	··	··	··	··	··	··	0
197	Taiwan (June 2009-March 2011) (Chuang et al, 2012)[83]	Flu AND ICU	Census-derived estimate	Nasal and Throat swab; PCR, viral culture, and HI	··	··	··	··	··	··	··	0.1
e25	Germany (Jan 2005-Dec 2012) (Von Der Beck et al, 2017)[67]	ARI AND MV	Census derived estimate	··	··	··	··	··	··	··	··	0
up_12	Tone and Cinkasse districts, Togo; mixed urban-rural (Aug 2011-Oct 2013; May 2014-Jul 2015) (Moïsi and colleagues)	ALRI AND ICU; MV; danger signs	Census-derived estimate	NPA; PCR	··	··	··	··	··	··	··	0
f071	Spain (2010-2016) (Oliva, J et al, 2018) [30]	ALRI AND ICU	Census-derived estimate	Respiratory swab; ··	··	··	··	··	··	··	··	0

Supplementary table 28: Description of studies reporting IFV-ALRI in-hospital case fatality ratios (hCFRs) in children under 5 years*

ID	Location (reference)	Case definition	Specimen and diagnostic test	0-5 m		6-11 m		12-59 m		0-59 m	
				Cases	hCFR (%)	Cases	hCFR (%)	Cases	hCFR (%)	Cases	hCFR (%)
b_10	Bondo district, Kenya (Jun 2007-May 2009) (from Nair et al, 2011) [10]	ALRI	NPS or OPS; PCR	··	··	··	··	··	··	67	4.5
b_13	Multistate, USA (Oct-Apr 2003-2008) (Dawood et al, 2010) [38]	ALRI	NPS or OPS; Viral culture, DFA, IFA, RIDT, PCR	1121	0.3	··	··	··	··	3023	0.2
b_15	Gipuzoka, Spain (Jul 2001-Jun 2004) (Montes et al, 2005) [40]	ARI	NPA; Viral culture and PCR	··	··	··	··	··	··	70	0
b_17	Leicester, United Kingdom (Oct 2001- Jun 2002) (Nicholson et al, 2006) [42]	ARI	Nasal and throat swabs; PCR	··	··	··	··	··	··	33	0
b_20	Hong Kong (July 1997- June 1999) (Nelson et al, 2007) [44]	ARI	NPA; Viral culture and serology	··	··	··	··	··	··	5471	0.1
b_23	South Australia, Australia (1996-2006) (D'Onise et al, 2008) [62]	ARI	··; Viral Culture, and PCR	··	··	··	··	··	··	626	0.6
b_26	Salt Lake County, Utah, USA (Jul 2001-Jun 2004) (Ampofo et al, 2006) [49]	ALRI	NPA; DFA	92	0	··	··	··	··	325	0.3
b_27	Philadelphia, USA (Jul 2000- Jun 2004) (Coffin et al, 2007)[50]	All	Nasal aspirates; SPIA, DFA and viral culture	··	··	··	··	··	··	573	0.9
b_32	Jordan, Oman, and Egypt (Oct 2007-Nov 2009) (from Nair et al, 2011) [10]	ALRI; Chest wall indrawing	··; PCR	··	··	··	··	··	··	77	1.3
b_33	Hong Kong (Jan-Jun 2005) (Kwong et al, 2009) [84]	ARI; Fever	··; RIDT	··	··	··	··	··	··	86	1.2
b_34	Canada (2003-2004) (Moore et al, 2006) [85]	ARI; Fever	··; Culture or DFA	116	0	··	··	··	··	423	0.2
b_35	Parana State, Brazil (Jan 1996-Dec 2001) (Coelho et al, 2007) [86]	ARI	NPA or BAL; IFA and culture	··	··	··	··	··	··	45	6.7
b_36	Kuala Lumpur, Malaysia (2002-2007) (Sam et al, 2010) [87]	ARI	··; DFA and culture	··	··	··	··	73	2.7	116	2.6

* NPA: nasopharyngeal aspirate. NPS: nasopharyngeal swab. NPW: nasopharyngeal wash. OPS: oropharyngeal swab. BAL: bronchoalveolar lavage. PCR: polymerase chain reaction. IFA: indirect immunofluorescence assay. DFA: direct fluorescent antibody. ELISA: enzyme-linked immunosorbent assay. IF: immunofluorescence. HI: hemagglutination-inhibition assay. RIDT: rapid influenza diagnostic test. SPIA: Solid-phase immunoassay. EIA: enzyme immunoassay. MN: micro-neutralization assay. TRFIA: time-resolved fluoroimmunoassay. ARI: any respiratory infections/symptoms (acute one of cough, sore throat, shortness of breath, coryza); ICD-codes for influenza & pneumonia (excluding non-respiratory manifestations); or acute infections (fever or <35 deg C) with any respiratory signs. ALRI: physician-diagnosed pneumonia and/or bronchiolitis, or WHO definition for ALRI applied by a health worker requiring hospitalisation. All: all diagnosis related to influenza (including influenza & pneumonia and other non-respiratory illnesses). SpO2: oxygen saturation.

ID	Location (reference)	Case definition	Specimen and diagnostic test	0-5 m		6-11 m		12-59 m		0-59 m	
				Cases	hCFR (%)	Cases	hCFR (%)	Cases	hCFR (%)	Cases	hCFR (%)
b_8	Santa Rosa, Guatemala (Jan 2008-Apr 2009) (from Nair et al, 2011) [10]	ARI	NPS or OPS; PCR	··	··	··	··	··	··	8	25
e11	Islamabad Pakistan (Mar 2011-Apr 2012) (Bashir et al, 2017) [88]	ALRI	NPS or OPS; PCR	··	··	··	··	··	··	··	··
e18	Izmir, Turkey (Oct 2014-May 2015) (Kanik et al, 2016) [89]	ALRI	NPS; PCR	··	··	··	··	··	··	··	··
e20	Australia (Jan 2011-Dec 2013) (Li-Kim-Moy et al, 2017) [90]	ARI	··; PCR	99	0	80	0	297	0	476	0
e25	Germany (Jan 2005-Dec 2012) (Von Der Beck et al, 2017) [67]	ARI	··	··	··	··	··	··	··	6328	0.4
e9	Turkey (Dec 2012-March 2016) (Acar et al, 2017) [91]	ARI; Fever	NPS; PCR	8	0	··	··	··	··	61	3.3
1808	Egypt, Jordan, Oman, Qatar and Yemen (2007-2014) (Horton et al, 2017) [92]	ARI	NPS and OPS; PCR	··	··	··	··	··	··	762	2.5
f023	Multi-country (2010-2014) (Dananche et al, 2018) [93]	ALRI	Nasal swab and nasal aspirate; PCR	··	··	··	··	··	··	86	3.5
f025	Bucharest, Romania (2016-2017) (Draganescu et al, 2018) [94]	ARI	NPS or nasal swab; PCR	··	··	··	··	··	··	58	1.7
f029	Multi-counrty (2013) (El Omeiri et al, 2018) [95]	ALRI-Fever	Combined nasal and OP swab or pharyngeal wash; PCR	··	··	··	··	··	··	··	··
f064	Kutaisi, Georgia (2014-2017) (Machablishvili et al, 2018) [96]	ARI-Fever	Oral and nasal swab; PCR	··	··	··	··	165	1.2	242	0.8
f067	Maputo, Mozambique (2014-2016) (Nguenha et al, 2018) [3]	ARI-Fever	NPS or OPS; PCR	9	11.1	14	0	40	0	63	1.6
f071	Spain (2010-2016) (Oliva et al, 2018) [30]	ALRI	Respiratory swab; PCR	··	··	··	··	··	··	426	0.9
f090	Deli Serdang,Balikpapan, Gunung Kidul, Indonesia (2013-2016) (Susilarini et al, 2018) [76]	ARI-Fever	Respiratory specimen; PCR	··	··	··	··	··	··	114	0
f099	Catalonia, Spain (2014-2016) (Torner et al, 2018) [97]	ALRI	NPS; PCR and culture	··	··	··	··	··	··	167	1.2
f168	Shenzhen, China (2014-2015) (Zhang et al, 2016) [98]	ALRI	NPS; DFA	··	··	··	··	··	··	127	0.8
f173	Wuhan, China (2016-2017) (Zhu et al, 2018) [99]	ARI	pharyngeal specimen; PCR	··	··	··	··	··	··	··	··
up_11	Siaya County, Kenya; rural (2010-2014) (Chaves and colleagues)	ALRI	NPS or OPS; PCR	9	0	10	0	38	2.6	57	1.8
up_12	Tone and Cinkasse districts, Togo; mixed urban-rural (Aug	ALRI	NPA; PCR	1	0	5	0	14	0	20	0

ID	Location (reference)	Case definition	Specimen and diagnostic test	0-5 m		6-11 m		12-59 m		0-59 m	
				Cases	hCFR (%)	Cases	hCFR (%)	Cases	hCFR (%)	Cases	hCFR (%)
	2011-Oct 2013; Aug 2014-July 2015) (Moïsi and colleagues)										
up_13	Soweto, Gauteng, South Africa; urban (Mar 1998-Oct 2005) (Madhi and colleagues)	ALRI	NPA; IFA	28	3.6	20	10	50	2	98	4.1
up_14	Amman, Jordan; urban (Mar 2010-Mar 2013) (Khuri-Bulos and colleagues)	ALRI	Nasal/throat swabs; PCR	35	5.7	22	0	··	··	··	··
up_15	Manhiça, Mozambique; rural (Jan 2011-Jun 2014) (Bassat and colleagues)	ALRI	NPA; PCR	5	0	7	14.3	11	0	23	4.3
up_16	Kilifi, Kenya; rural and semi-urban (Jan 2007- Dec 2016) (Nokes and colleagues)	ALRI	NPS; PCR	33	6.1	26	11.5	59	3.4	118	5.9
up_17	Muang District, Nakhon Phanom Province, Thailand (PERCH); rural (2012-2013) (O'Brien and colleagues)	ALRI	NP/OP and induced sputum; PCR	··	··	1	0	1	0	2	0
up_18	Muang District, Sa Kaeo Province, Thailand; rural (2012-2013) (O'Brien and colleagues)	ALRI	NP/OP and induced sputum; PCR	··	··	··	··	3	33.3	3	33.3
up_19	Nha Trang city, Vietnam; urban and sub-urban (2008-2013) (Yoshida and colleagues)	ALRI	NP specimens; PCR	4	0	6	0	35	0	45	0
up_20	Berlin, Germany; urban (2010-2014) (Rath and colleagues)	ALRI	NPS; PCR	31	0	22	0	98	0	151	0
up_21	Basse, Upper River Region, Gambia (PERCH); rural (Nov 2011-Nov 2013) (O'Brien and colleagues)	ALRI	NPS, OPS, induced sputum; PCR	17	0	4	0	12	0	33	0
up_23	Nakhon Phanom and Sa Kaeo Province, Thailand; rural (Jan 2005-Dec 2011) (Thamtithiwat and colleagues)	ALRI	NPS; PCR	34	0	110	0	559	0.2	703	0.1
up_24	Pune district, India; rural (May 2009-Apr 2013) (Hirve and colleagues)	ALRI	NPS; PCR	··	··	2	0	2	0	4	0
up_26	David City, Panama (2014-2016) (Jara and colleagues)	ALRI	NPS or OPS; PCR	14	21.4	15	13.3	30	0	59	8.5
up_27	Ciudad de Buenos Aires, Argentina; urban (Jun 2008-Dec 2010) (Echavarria and colleagues) **Error! Bookmark not defined.**	ALRI	NPA; IFA	5	0	2	0	5	0	12	0

ID	Location (reference)	Case definition	Specimen and diagnostic test	0-5 m		6-11 m		12-59 m		0-59 m	
				Cases	hCFR (%)	Cases	hCFR (%)	Cases	hCFR (%)	Cases	hCFR (%)
up_28	Turku, Finland; urban (Jan 2010-Jun 2012) (Heikkinen and colleagues)	ALRI	Nasal swabs; TRFIA	12	0	4	0	12	8.3	28	3.6
up_29	Aurora, Colorado, United States; urban (Jan 2011-Oct 2015) (Simões and colleagues)	ALRI	··	134	0	106	1.9	390	0.8	630	0.8
up_3	Paarl, Western Province, South Africa (June 2012-Dec 2016) (Zar and colleagues)	ALRI	NPS; PCR	10	10	4	0	4	0	18	5.6
up_30	Region VI Buenos Aires Province, Argentina; urban /slums/semi-rural (2011-2013) (Polack and colleagues)	ALRI; Wheezing; SpO2 <93%	NPA; PCR	26	3.8	20	0	··	··	··	··
up_31	Quetzaltnango, Guatemala (2010-2016) (McCracken and colleagues)	ALRI	NPS and OPS; PCR	36	0	19	5.3	46	2.2	101	2.0
up_32	Cuilapa, Santa Rosa, Guatemala (2010-2016) (McCracken and colleagues)	ALRI	NPS and OPS; PCR	17	11.8	6	33.3	27	7.4	50	12.0
up_33	Tagbilaran City, Bohol, Philipines; Dauis, Baclayon, Panglao, Cortes, Balilihan, Bohol, Philippines; mixed urban-rural (Jul 2000-Dec 2004) (Lucero and colleagues)	ALRI	NPA; Viral culture	11	9.1	19	0	··	··	··	··
up_34	Valencia Region, Spain (2014-2017) (Mira Iglesias and colleagues)	All	NPS and nasal swabs; RT-PCR	39	2.6	16	0	71	0	126	0.8
up_4	Buenos Aires, Argentina; urban (2009-2016) (Gentile and colleagues)	ALRI	NPA; PCR	21	4.8	29	0	49	4.1	99	3.0
up_40	Karachi, Sind, Pakistan (Jan 2009-Feb 2018) (Abbas and colleagues)	ALRI	NP secretions; PCR	··	··	··	··	13	38.5	22	31.8
up_41	Soweto, Gauteng, South Africa (2015-2017) (Madhi and colleagues)	ALRI; Sepsis	NPS; PCR	45	0	61	0	47	0	153	0
up_42	Rabat, Morocco (Nov 2010-Dec 2011) (Bassat and colleagues)	ALRI	NPA; PCR	3	0	9	0	16	6.2	28	3.6
up_43	Lusaka, Zambia (2011-2013) (O'Brien and colleagues)	ALRI	NPS, OPS, Induced sputum; ··	11	27.3	7	14.3	13	15.4	31	19.4
up_44	Soweto, South Africa (2011-2013) (O'Brien and colleagues)	ALRI	NPS, OPS, Induced sputum; ··	18	0	15	6.7	21	4.8	53	3.8

ID	Location (reference)	Case definition	Specimen and diagnostic test	0-5 m		6-11 m		12-59 m		0-59 m	
				Cases	hCFR (%)	Cases	hCFR (%)	Cases	hCFR (%)	Cases	hCFR (%)
up_45	Matlab, Bangladesh (2012-2013) (O'Brien and colleagues)	ALRI	NPS, OPS, Induced sputum; ··	3	0	4	0	0	··	7	0
up_46	Dhaka, Bangladesh (2012-2013) (O'Brien and colleagues)	ALRI	NPS, OPS, Induced sputum; ··	1	0	0	··	2	0	3	0
up_5	Klerksdorp, North West Province, South Africa; peri-urban (2013-2015) (Cohen and colleagues)	ALRI; Sepsis	NPA; PCR	5	0	7	0	18	0	30	0
up_6	Pietermaritzburg, Kwa-Zulu Natal Province, South Africa; peri-urban (2013-2015) (Cohen and colleagues)	ALRI; Sepsis	NPA; PCR	9	11.1	14	0	15	0	38	2.6
up_7	Soweto, Gauteng, South Africa; urban (2009-2012) (Cohen and colleagues)	ALRI; Sepsis	NPA; PCR	70	1.4	61	0	104	1	235	0.9
up_8	Concepcion, Chile; mixed urban/rural (2012-2013) (Fasce and colleagues)	ALRI	NPA and NPS; PCR	12	8.3	6	0	18	0	36	2.8
up_9	Iquique, Chile; mixed urban-rural (2012-2013) (Fasce and colleagues)	ALRI	NPA; PCR	5	0	9	0	17	0	31	0

Supplementary table 29: Quality of studies reporting hospitalisation rates of IFV- (severe and very severe) ALRI in children under five years[*]

ID	Location (Study period)	Study design	Adjustment for health utilization[†]	Patient group excluded	Case definition	Sampling strategy	Diagnostic test	Hypoxemia[‡]
32	United States of America;2003-2012	Low	Low	Low	High	High	High	NA
34[§]	Hong Kong;2004-2011	Low	Low	Low	High	Low	Low	NA
66	Australia;1997-2013	High	Low	Low	Low	High	High	NA
80	Hong Kong;2005-2011	High	Low	Low	High	High	Low	NA
111	United States of America;2003-2010	Low	Low	Low	High	High	High	NA
116	Bangladesh;2010-2014	Low	Low	Low	High	Low	Low	NA
118	Denmark;2009-2011	High	Low	Low	Low	High	Low	NA
141	Philippines;2009-2011	Low	High	Low	High	Low	Low	NA
146	France;2009-2013	High	Low	Low	Low	High	High	NA
156	China;2010-2012	Low	Low	Low	High	Low	Low	NA
197	Taiwan;2009-2011	High	Low	Low	Low	High	Low	NA
422	El Salvador;2009-2012	High	Low	Low	High	Low	High	NA
423	Guatemala;2009-2012	High	Low	Low	High	Low	High	NA
424	Honduras;2009-2012	High	Low	Low	High	Low	High	NA
425	Nicaragua;2009-2012	High	Low	Low	High	Low	High	NA
4001	Oman;2008-2013	Low	Low	Low	Low	Low	Low	NA
4002	Oman;2008-2013	Low	Low	Low	High	Low	Low	NA
4003	Oman;2008-2013	Low	Low	Low	High	Low	Low	NA
b_10	Kenya;2007-2009	Low	Low	Low	Low	Low	Low	NA
b_11	Germany;1999-2001	Low	Low	Low	High	Low	Low	NA
b_13	United States of America;2003-2008	Low	Low	Low	High	High	High	NA
b_14	Germany;1996-2000	High	Low	Low	High	Low	Low	NA
b_15	Spain;2001-2004	High	Low	Low	High	Low	Low	NA
b_16	United Kingdom;2002-2004	Low	Low	Low	High	Low	Low	NA
b_17	United Kingdom;2001-2002	Low	Low	Low	High	High	Low	NA
b_18	Spain;1997-2003	High	Low	Low	High	High	Low	NA
b_19	Vietnam;2007-2008	Low	Low	Low	High	Low	Low	NA
b_20	Hong Kong;1997-1999	High	Low	Low	High	High	Low	NA
b_21	Hong Kong;2003-2006	Low	Low	Low	High	Low	High	NA
b_22	China;2007-2008	High	High	Low	High	High	High	NA
b_23	Australia;1996-2006	High	Low	Low	High	High	Low	NA
b_24	United States of America;2001-2004	Low	Low	Low	High	Low	Low	NA
b_25	United States of America;2000-2001	Low	Low	Low	High	Low	Low	NA
b_26	United States of America;2001-2004	High	Low	Low	High	High	High	NA

[*] Low: low risk of bias; high: high risk of bias
[†] Only for studies reporting hospitalisation rates
[‡] NA: not applicable.
[§] Combining Flu A and B in study ID - e12.

ID	Location (Study period)	Study design	Adjustment for health utilization †	Patient group excluded	Case definition	Sampling strategy	Diagnostic test	Hypoxemia‡
b_27	United States of America;2000-2004	High	High	Low	High	High	Low	NA
b_28	United States of America;2003-2005	Low	Low	Low	High	Low	High	NA
b_3	Japan;2002-2008	Low	Low	Low	High	Low	High	NA
b_30	United States of America;1996-1998	High	Low	Low	High	High	High	NA
b_31	Brazil;1987-1989	Low	High	Low	Low	Low	Low	NA
b_40	United States of America;1974-1999	Low	Low	Low	High	Low	Low	NA
b_8	Guatemala;2008	Low	Low	Low	High	Low	Low	NA
b_9	Mozambique;2006-2007	Low	Low	Low	Low	Low	Low	NA
e12	Hong Kong;2004-2014	Low	Low	Low	High	Low	High	NA
e13	Philippines;2012-2014	Low	Low	Low	High	Low	Low	NA
e14	Ghana;2013-2015	Low	Low	High	High	Low	Low	NA
e16	United Kingdom;2010-2015	Low	Low	Low	High	Low	Low	NA
e25	Germany;2005-2012	High	Low	Low	High	High	High	NA
e26	Australia;2006-2015	High	Low	Low	High	High	High	NA
s103	United States of America;2010-2012	Low	Low	High	Low	Low	Low	NA
s134	United States of America;2004-2009	Low	Low	Low	High	Low	Low	NA
s138	Greece;2002-2005	Low	Low	Low	High	Low	Low	NA
s140	Finland;1988-2004	Low	Low	Low	High	High	High	NA
s205	United Kingdom;2012-2013	High	Low	Low	Low	High	Low	NA
s207	Spain;2011-2012	Low	Low	High	High	Low	Low	NA
s304	United States of America;2010-2011	Low	Low	Low	High	High	High	NA
f023	Multi-country; 2010-2014	Low	NA	High	Low	Low	Low	NA
f025	Romania; 2016-2017	Low	NA	Low	High	Low	Low	NA
f029	Multi-country; 2013	Low	NA	High	High	High	Low	NA
f064	Georgia; 2014-2017	Low	NA	Low	High	Low	Low	NA
f067	Mozambique; 2014-2016	Low	NA	Low	High	Low	Low	NA
f071	Spain; 2010-2016	High	Low	Low	Low	High	Low	NA
f090	Indonesia; 2013-2016	Low	Low	Low	High	Low	Low	NA
f099	Spain; 2010-2015	Low	NA	Low	Low	Low	Low	NA
f168	China; 2014-2015	High	NA	Low	Low	High	High	NA
f173	China; 2016-2017	High	NA	Low	High	High	Low	NA
f008	Congo, Dem. Rep; 2013-2015	Low	Low	Low	Low	Low	Low	NA
f047A	Cambodia; 2015-2016	Low	Low	Low	High	Low	Low	NA
f047B	Cambodia; 2015-2016	Low	Low	Low	High	Low	Low	NA
f047C	Cambodia; 2015-2016	Low	Low	Low	High	Low	Low	NA
f070	Rwanda; 2012-2014	Low	Low	Low	High	Low	Low	NA
f087	Chile; 2012-2014	Low	Low	Low	High	Low	Low	NA
f118	China; 2014-2016	Low	Low	Low	High	High	Low	NA
f207	China; 2017-2018	Low	Low	Low	High	Low	Low	NA
f231	Oman; 2012-2015	High	Low	Low	High	High	Low	NA

ID	Location (Study period)	Study design	Adjustment for health utilization †	Patient group excluded	Case definition	Sampling strategy	Diagnostic test	Hypoxemia‡
up_10	Kenya;2010-2014	Low	Low	Low	Low	Low	Low	Low
up_11	Kenya;2010-2014	Low	Low	Low	Low	Low	Low	Low
up_12	Togo;2011-2013; 2014-2015	Low	Low	Low	Low	Low	Low	Low
up_13	South Africa;1998 to 2005	Low	Low	High	Low	Low	High	High
up_14	Jordan;2010-2013	Low	Low	Low	Low	Low	Low	Low
up_15	Mozambique;2011-2014	Low	High	Low	Low	Low	Low	Low
up_16	Kenya;2007-2016	Low	Low	Low	Low	Low	Low	Low
up_17	Thailand;2012-2013	Low	Low	High	Low	Low	Low	NA
up_18	Thailand;2012-2013	Low	Low	High	Low	Low	Low	NA
up_19	Vietnam;2008-2013	Low	Low	High	Low	Low	Low	Low
up_21	Gambia;2011-2013	Low	Low	High	Low	Low	Low	NA
up_22	Israel;2011-2016	Low	High	Low	High	High	Low	NA
up_23	Thailand;2005-2011	Low	Low	Low	High	Low	Low	NA
up_24	India;2009-2013	Low	Low	Low	Low	Low	Low	High
up_25	Bangladesh;2007-2015	Low	Low	Low	Low	Low	Low	NA
up_26	Pakistan;2012-2014	Low	Low	Low	Low	High	Low	High
up_27	Argentina;2008-2010	Low	Low	High	Low	Low	High	High
up_28	Finland;2010-2012	Low	High	Low	Low	Low	High	NA
up_29	United States of America;2011-2015	High	High	Low	Low	High	High	High
up_3	South Africa;2012-2016	Low	Low	Low	Low	Low	Low	NA
up_30	Argentina;2011-2013	Low	Low	Low	Low	Low	Low	High
up_31	Guatemala;2010-2016	Low	Low	Low	Low	Low	Low	Low
up_32	Guatemala;2010-2016	Low	Low	Low	Low	Low	Low	Low
up_33	Philipines;2000-2004	Low	Low	High	Low	Low	Low	Low
up_34	Spain;2014-2017	Low	Low	Low	High	Low	Low	High
up_4	Argentina;2009-2016	Low	High	Low	Low	Low	Low	Low
up_41	South Africa;2015-2017	Low	Low	Low	Low	High	Low	NA
up_5	South Africa;2013-2015	Low	High	Low	Low	Low	Low	NA
up_6	South Africa;2013-2015	Low	High	Low	Low	Low	Low	NA
up_602	South Africa;2011-2013	Low	Low	Low	Low	Low	Low	NA
up_7	South Africa;2009-2012	Low	Low	Low	Low	Low	Low	NA
up_8	Chile;2012-2013	Low	High	Low	Low	Low	Low	NA
up_9	Chile;2012-2013	Low	High	Low	Low	Low	Low	NA

Supplementary table 30: Quality of studies reporting IFV-ALRI in-hospital case fatality ratios (hCFRs) in children under five years

ID	Location (Study period)	Study design	Patient group excluded	Case definition	Sampling strategy
1808	Egypt, Jordan, Oman, Qatar and Yemen; 2007-2014	Low	Low	High	High
up_4	Argentina; 2009-2016	Low	Low	Low	Low
up_6	South Africa; 2013-2015	Low	Low	Low	Low
up_5	South Africa; 2013-2015	Low	Low	Low	Low
up_7	South Africa; 2009-2012	Low	Low	Low	Low
up_8	Chile; 2012-2013	Low	Low	Low	Low
up_9	Chile; 2012-2013	Low	Low	Low	Low
up_10	Kenya; 2010-2014	Low	Low	Low	High
up_11	Kenya; 2010-2014	Low	Low	Low	High
up_12	Togo; 2011-2013; 2014-2015	Low	Low	Low	Low
up_13	South Africa; 1998 to 2005	Low	High	Low	Low
up_14	Jordan; 2010-2013	Low	Low	Low	Low
up_15	Mozambique; 2011-2014	Low	Low	Low	Low
up_16	Kenya; 2007-2016	Low	Low	Low	High
up_17	Thailand; 2012-2013	Low	High	Low	Low
up_18	Thailand; 2012-2013	Low	High	Low	Low
up_19	Viet Nam; 2008-2013	Low	High	Low	Low
up_20	Germany; 2010-2014	Low	Low	Low	Low
up_21	Gambia; 2011-2013	Low	High	Low	Low
up_23	Thailand; 2005-2011	Low	Low	High	High
up_24	India; 2009-2013	Low	Low	Low	Low
up_27	Argentina; 2008-2010	Low	High	Low	Low
up_28	Finland; 2010-2012	Low	Low	Low	Low
up_29	United States of America; 2011-2015	High	Low	Low	High
up_30	Argentina; 2011-2013	Low	Low	Low	Low
up_32	Guatemala; 2010-2016	Low	Low	Low	Low
up_31	Guatemala; 2010-2016	Low	Low	Low	Low
up_33	Philippines; 2000-2004	Low	High	Low	High
up_26	Panama; 2012-2014	Low	Low	Low	High
b_32	Jordan, Oman, Egypt; 2007-2009	Low	Low	Low	Low
b_8	Guatemala; 2008	Low	Low	High	Low
b_10	Kenya; 2007-2009	Low	Low	Low	Low
b_15	Spain; 2001-2004	High	Low	High	Low
b_17	United Kingdom; 2001-2002	Low	Low	High	High

ID	Location (Study period)	Study design	Patient group excluded	Case definition	Sampling strategy
b_26	United States of America; 2001-2004	High	Low	High	High
b_27	United States of America; 2000-2004	High	Low	High	High
b_23	Australia; 1996-2006	High	Low	High	High
b_33	China; 2005	High	High	High	High
b_34	Canada; 2003-2004	High	Low	High	High
b_13	United States of America; 2003-2008	Low	Low	High	High
b_35	Brazil; 1996-2001	High	Low	High	High
b_20	China; 1997-1999	High	Low	High	High
b_36	Malaysia; 2002-2007	High	Low	High	High
up_34	Spain; 2014-2017	Low	Low	High	High
up_40	Pakistan; 2010-2018	High	Low	Low	High
up_41	South Africa; 2015-2017	Low	Low	Low	High
up_42	Morocco; 2010-2011	Low	Low	Low	Low
up_3	South Africa; 2012-2016	Low	Low	Low	Low
e25	Germany; 2005-2012	High	Low	High	High
e9	Turkey; 2012-2016	High	Low	High	High
e11	Pakistan; 2011-2012	Low	High	Low	Low
e18	Turkey; 2014-2015	High	High	Low	Low
e20	Australia; 2011-2013	High	Low	High	High
f023	Multi-county; 2010-2014	Low	High	Low	Low
f025	Bucharest, Romania; 2016-2017	Low	Low	High	Low
f029	Multi-country; 2013	Low	High	High	High
f064	Georgia; 2014-2017	Low	Low	High	Low
f067	Mozambique; 2014-2016	Low	Low	High	Low
f071	Spain; 2010-2016	High	Low	Low	High
f090	Indonesia; 2013-2016	Low	Low	High	Low
f099	Spain; 2010-2015	Low	Low	Low	Low
f168	China; 2014-2015	High	Low	Low	High
f173	China; 2016-2017	High	Low	High	High
up_43	Zambia; 2011-2013	Low	High	Low	Low
up_44	South Africa; 2011-2013	Low	High	Low	Low
up_45	Bangladesh; 2012-2013	Low	High	Low	Low
up_46	Bangladesh; 2012-2013	Low	High	Low	Low

Tables of meta-estimates of IFV-respiratory infections by finer age band in children 0-59 months

Supplementary table 31: Incidence rate meta-estimates of IFV-episodes in children under five years

	0-27 d		1-2 m		3-5 m		6-11 m		12-23 m		24-59 m	
	**No** *	**Rate**†	**No**	**Rate**	**No**	**Rate**	**No**	**Rate**	**No**	**Rate**	**No**	**Rate**
Developing	6	17.4 (4.4-66.2)	6	39.4 (16.2-92.4)	3	76.2 (28.9-186.1)	2	165.9 (93.2-277.8)	4	167.3 (106.5-253)	4	196.8 (109.1-329)
Industrialised	..	..	..	..	..	..	..	..	3	187.4 (116.9-286.7)	4	79.1 (25.7-218.2)
Low and lower middle income	5	16.4 (3.1-80.8)	5	31.7 (12.1-80.5)	3	76.2 (28.9-186.1)	2	165.9 (93.2-277.8)	4	167.3 (106.5-253)	4	196.8 (109.1-329)
High income	..	..	..	..	..	..	..	..	3	187.4 (116.9-286.7)	4	79.1 (25.7-218.2)

* No: number of studies.
† Rate: incidence rate per 1,000 children pear year.

Supplementary table 32: Incidence rate meta-estimates of IFV-ALRI in children under five years

	0-27 d		1-2 m		3-5 m		6-11 m		12-23 m		24-59 m	
	No [*]	Rate[†]	No	Rate	No	Rate	No	Rate	No	Rate	No	Rate
Developing	5	0.2 (0-129.2)	5	6.7 (1.1-40.4)	5	12.8 (5.7-28.4)	5	27.6 (23.4-32.6)	7	30.7 (20.3-46.2)	5	7.3 (2.9-18)
Industrialised	··	··	··	··	··	··	··	··	2	11.7 (8.2-16.9)	··	··
Low and lower middle income	··	··	4	3.4 (0.9-12.2)	4	8 (5.6-11.5)	4	28 (23.6-33.3)	6	32.8 (20.7-51.6)	4	10.4 (5-21.8)
High income	··	··	··	··	··	··	··	··	2	11.7 (8.2-16.9)	··	··

[*] No: number of studies.
[†] Rate: incidence rate per 1,000 children per year.

Supplementary table 33: Incidence rate meta-estimates of IFV-severe and very severe ALRI in children under five years

	0-27 d		1-2 m		3-5 m		6-11 m		12-23 m		24-59 m	
	**No***	**Rate†**	**No**	**Rate**	**No**	**Rate**	**No**	**Rate**	**No**	**Rate**	**No**	**Rate**
**IFV-severe ALRI**												
Developing	11	5.1 (2.1-12)	6	2.6 (0.3-19.4)	4	2.8 (0.3-30.3)	4	6.1 (2.2-16.4)	5	4 (1.7-9.4)	4	0.7 (0.4-1.4)
Low and lower middle income	10	4.4 (1.7-11.4)	5	1.3 (0.3-6.1)	3	1 (0.1-14.2)	3	3.9 (1.4-11.1)	4	2.6 (1.2-5.4)	3	0.8 (0.4-1.6)
**IFV-very severe ALRI**												
Developing	4	0.3 (0-37.4)	4	0.1 (0-48.3)	4	0.1 (0-81)	4	1 (0.3-3.5)	4	1.2 (0.4-3.9)	3	0.1 (0-1.3)
Low and lower middle income	··	··	3	0.1 (0-59.9)	3	0.2 (0-86.8)	3	0.8 (0.2-4)	3	0.8 (0.2-3.4)	2	0.1 (0-1.6)

* No: number of studies.
† Rate: incidence rate per 1,000 children per year.

Supplementary table 34: Hospitalisation rate meta-estimates of IFV-ALRI in children under five years

Region	0-27 d		1-2 m		3-5 m		6-11 m		12-23 m		24-59 m	
	No *	Rate †	No	Rate	No	Rate	No	Rate	No	Rate	No	Rate
Developing	15	0.8 (0.4-1.7)	18	1.9 (1.2-3.0)	17	2 (1.5-2.7)	21	2.7 (1.7-4.3)	36	1.6 (1-2.5)	36	0.7 (0.4-1.1)
Industrialised‡	2	4 (0.9-18.2)	2	9.0 (4.0-20.2)	3	1.6 (0.3-7.7)	7	2.6 (0.9-7.6)	12	1.6 (0.8-3.3)	17	0.5 (0.3-1)
Low and Lower middle income	8	0.6 (0.3-1.5)	10	1.6 (0.9-2.8)	10	1.6 (1.2-2.2)	11	1.5 (1-2.4)	14	1.4 (0.8-2.6)	15	0.5 (0.2-0.8)
Upper middle income	7	1 (0.3-3.8)	8	2.6 (1.3-5.3)	7	2.8 (1.8-4.4)	8	3.8 (1.7-8.3)	16	1.4 (0.7-2.9)	15	0.6 (0.3-1.4)
High income	2	4 (0.9-18.2)	2	9.0 (4.0-20.2)	3	1.6 (0.3-7.7)	9	3.5 (1.4-8.8)	18	2 (1.1-3.7)	23	0.8 (0.4-1.4)

* No: number of studies. Data in parentheses were numbers of imputed studies.
† Rate: hospitalisation rate per 1,000 children per year.
‡ For 0-27 d and 1-2 m, only two studies in Finland and Israel provided population-at-risk. Much higher rates were reported in the study in Israel, in which ARI and acute febrile illness was used as the case definition.

Supplementary table 35: Hospitalisation rate meta-estimates of IFV-ALRI using the classic random model

Region	0-27 d		1-2 m		3-5 m		6-11 m		12-23 m		24-59 m	
	No*	Rate †	No	Rate	No	Rate	No	Rate	No	Rate	No	Rate
Developing	15	1.6 (0.8-3.1)	18	2.4 (1.6-3.6)	17	2.3 (1.7-3.1)	21	2.8 (1.7-4.6)	36	1.9 (1.3-2.7)	36	0.8 (0.5-1.1)
Industrialised	2	4 (0.5-32.1)	2	8.9 (2.9-27.4)	3	1.7 (0.3-10.4)	7	2.6 (0.7-10)	12	1.7 (0.7-3.8)	17	0.5 (0.3-0.9)
Low and lower middle income	8	1.1 (0.6-2.2)	10	2.3 (1.6-3.2)	10	1.8 (1.4-2.4)	11	1.7 (1.1-2.7)	14	1.6 (0.8-3.2)	15	0.5 (0.2-1.0)
Upper middle income	7	2.2 (0.6-8.2)	8	2.9 (1.4-6.2)	7	2.9 (1.8-4.9)	8	3.9 (1.3-11.8)	16	1.7 (0.7-3.8)	15	0.7 (0.2-1.8)
High income	2	4 (0.5-32.1)	2	8.9 (2.9-27.4)	3	1.7 (0.3-10.4)	9	3.6 (1.1-11.1)	18	2 (0.9-4.4)	23	0.8 (0.4-1.8)

* No: number of studies. Data in parentheses were numbers of imputed studies.
† Rate: hospitalisation rate per 1,000 children per year.

Supplementary table 36: Hospitalisation rate meta-estimates of IFV-ALRI by subtypes in children under five years.

	0-5 m		6-11 m		12-59 m	
	**No** [*]	**Rate** [†]	**No**	**Rate**	**No**	**Rate**
**In studies reporting IFV-A and IFV-B**						
All IFV						
Developing	21	2.5 (1.5-4)	18	2.5 (1.5-4.2)	24	0.7 (0.4-1.2)
Industrialised	3	4.1 (1.7-9.7)	2	3 (0.4-24)	3	0.8 (0.2-3.2)
IFV-A						
Developing	21	1.2 (0.6-2.5)	18	1.4 (0.7-2.6)	24	0.4 (0.2-0.7)
Industrialised	3	3.8 (1.6-8.7)	2	2 (0.2-26)	3	0.7 (0.2-2.7)
IFV-B						
Developing	21	0.6 (0.4-0.9)	18	0.5 (0.2-0.9)	24	0.1 (0.1-0.2)
Industrialised	3	0.4 (0.1-1.4)	2	0.7 (0.3-1.7)	3	0.2 (0-0.6)
**In studies reporting H1N109pdm, H3N2, and IFV-B**						
All IFV						
Developing	15	2 (1-3.9)	13	2.5 (1.3-4.8)	17	0.9 (0.4-1.7)
Industrialised	··	··	··	··	2	0.4 (0.2-0.7)
H1N109pdm						
Developing	15	0.4 (0.1-1)	13	0.5 (0.2-1.4)	17	0.2 (0.1-0.5)
Industrialised	··	··	··	··	2	0.1 (0.1-0.1)
H3N2						
Developing	15	0.5 (0.2-1.1)	13	0.5 (0.2-1.2)	17	0.3 (0.1-0.6)
Industrialised	··	··	··	··	2	0.2 (0.1-0.4)
IFV-B						
Developing	15	0.5 (0.2-1)	13	0.4 (0.2-1.1)	17	0.2 (0.1-0.3)
Industrialised	··	··	··	··	2	0.1 (0-0.1)

[*] No: number of studies.
[†] Rate: hospitalisation rate per 1,000 children per year.

Supplementary table 37: Hospitalisation rate meta-estimates of IFV-ALRI with hypoxemia in children under 5 years

	0-27 d		1-2 m		3-5 m		6-11 m		12-23 m		24-59 m	
	No [*]	Rate[†]	No	Rate	No	Rate	No	Rate	No	Rate	No	Rate
Developing	8	0.5 (0.2-1.2)	10	1 (0.6-2)	10	1.1 (0.8-1.4)	10	0.8 (0.5-1.1)	14	0.4 (0.2-0.8)	11	0.1 (0-0.3)
Industrialised	··	··	··	··	··	··	··	··	2	0.2 (0-3.2)	2	0 (0-0.8)
Low and lower middle income	7	0.6 (0.2-1.2)	8	1.1 (0.5-2.5)	8	1 (0.7-1.5)	8	0.7 (0.3-1.4)	9	0.4 (0.2-0.8)	8	0.1 (0.1-0.3)
Upper middle income	··	··	2	0.7 (0.3-2)	2	1.1 (0.6-2)	2	1 (0.7-1.6)	5	0.4 (0.1-2)	3	0.2 (0-1.3)
High income	··	··	··	··	··	··	··	··	2	0.2 (0-3.2)	2	0 (0-0.8)

[*] No: number of studies. Data in parentheses were the numbers of imputed studies.
[†] Rate: hospitalisation rate per 1,000 children per year.

Supplementary table 38: Hospitalisation rate meta-estimates of IFV-very severe ALRI in children under 5 years

	0-27 d		1-2 m		3-5 m		6-11 m		12-23 m		24-59 m		0-4 y	
	**No** *	**Rate** †	**No**	**Rate**	**No**	**Rate**	**No**	**Rate**	**No**	**Rate**	**No**	**Rate**	**No**	**Rate**
Developing	9	0.5 (0.2-1.2)	12	0.3 (0.1-1.3)	12	0.5 (0.3-1)	12	0.4 (0.2-0.8)	21	0.1 (0-0.3)	18	0.1 (0-0.1)		
Industrialised	··	··	2	0.5 (0.1-1.4)	2	0.2 (0.1-0.9)	2	0.1 (0-0.6)	4	0 (0-0.1)	4	0 (0-0)		
Low and lower middle income	7	0.5 (0.2-1.2)	9	0.7 (0.2-2.3)	9	0.9 (0.6-1.3)	9	0.5 (0.3-0.9)	10	0.2 (0-0.7)	9	0.1 (0-0.2)		
Upper middle income	··	··	3	0.1 (0-0.8)	3	0.2 (0.1-0.8)	3	0.3 (0.1-1)	9	0.1 (0-0.3)	7	0.1 (0-0.2)		
High income	··	··	2	0.5 (0.1-1.4)	2	0.2 (0.1-0.9)	2	0.1 (0-0.6)	6	0 (0-0.1)	6	0 (0-0)		

* No: number of studies. Data in parentheses was the number of imputed studies.
† Rate: hospitalisation rate per 1,000 children per year.

Supplementary table 39: Incidence rate and hospitalisation rate meta-estimates of IFV-disease by narrow age group among infants

	0-27 d		1-2 m		3-5 m		6-8 m		9-11 m		
	No *	Rate †	No	Rate	No	Rate	No	Rate	No	Rate	P-value for linear trend‡
**IFV-episodes**											
Developing	3	6.7 (0.5-79.2)	3	20.4 (5.6-71)	3	76.2 (28.9-186.1)	2	130.9 (75-218.5)	2	199.4 (113.9-325.4)	0.003
**IFV-ALRI**											
Developing	5	0.2 (0-129.2)	5	6.7 (1.1-40.4)	5	12.8 (5.7-28.4)	5	25.3 (20-31.9)	5	30.4 (24-38.4)	0.02
**IFV-severe ALRI**											
Developing	5	0.2 (0-129.9)	5	2.5 (0.2-30.3)	5	2.1 (0.2-22.6)	5	3.3 (0.7-15)	5	6.1 (2.4-15.2)	0.41
**Hospitalised IFV-ALRI**											
Developing	13	0.9 (0.4-1.8)	13	1.7 (1-3.1)	13	1.6 (1.1-2.3)	13	1.9 (1.2-3)	13	1.9 (1.1-3.3)	0.15
Industrialised§	2	4 (0.9-18.2)	2	9 (4-20.2)	2	1.9 (0.2-21.5)	2	2.3 (0.1-35)	2	3.6 (0.6-20.7)	0.72
**Hospitalised IFV-ALRI with hypoxemia**											
Developing	8	0.5 (0.2-1.2)	8	1.2 (0.7-2.3)	8	1 (0.7-1.5)	8	0.8 (0.6-1.2)	8	1 (0.6-1.7)	0.96
**Hospitalised IFV-very severe ALRI**											
Developing	8	0.4 (0.1-1.0)	12	0.3 (0.1-1.0)	12	0.4 (0.2-0.8)	12	0.3 (0.2-0.6)	12	0.4 (0.2-0.8)	0.72

* No: number of studies.
† Rate: hospitalisation rate per 1,000 children per year.
‡ By outcome and development status, we included a continuous variable for age group in the mixed-effects meta-regression model. For the continuous variable, we used central values of each age group, for example, 0.5 months for 0-27 d, 2 months for 1-2 m, 4.5 months for 3-5 m, 7.5 months for 6-8 m, and 10.5 months for 9-11 m.
§ Only two studies in Finland and Israel provided population-at-risk for narrow age groups during infancy. Much higher rates were reported in the study in Israel, in which ARI and acute febrile illness was used as the case definition.

Supplementary table 40: Information of testing in hospital ALRI cases/deaths in the studies reporting hCFRs of IFV-ALRI by three age groups - 0-5 m, 6-11 m, and 12-59 m

Location (Study period)	All ALRI cases	Tested cases	Proportion of test in ALRI cases	IFV-ALRI cases	All ALRI deaths	Tested deaths	Proportion of test in ALRI deaths	IFV-ALRI deaths
Australia; 2011-2013	··	··	··	476	··	··	··	0
Kenya; 2010-2014	1658	1156	0.7	57	54	21	0.4	1
Togo; 2011-2013; 2014-2015	165	155	0.9	20	4	2	0.5	0
South Africa; 1998-2005	2721	2602	1	98	170	138	0.8	4
Mozambique; 2011-2014	422	411	1	23	13	11	0.8	1
Mozambique; 2014-2016	··	··	··	63	··	··	··	1
Kenya; 2007-2016	4096	2994	0.7	118	186	102	0.5	7
Vietnam; 2008-2013	422	422	1	45	0	0	··	0
Germany; 2010-2014	2630	2630	1	151	9	9	1	0
Gambia; 2011-2013	638	626	1	33	22	17	0.8	0
Thailand; 2005-2011	29513	7895	0.3	703	94	16	0.2	1
Panama; 2012-2014	4087	912	0.2	59	31	5	0.2	5
Argentina; 2008-2010	25	25	1	12	0	0	··	0
Finland; 2010-2012	··	··	··	28	··	··	··	1
United States of America; 2011-2015	12164	··	··	630	51	··	··	5
South Africa; 2012-2016	239	206	0.9	18	3	2	0.7	1
Guatemala; 2010-2016	2158	1753	0.8	101	50	49	1	2
Guatemala; 2010-2016	1788	1499	0.8	50	57	56	1	6
Spain; 2014-2017	2698	1929	0.7	126	··	6	··	1
Argentina; 2010-2016	4931	4666	0.9	99	60	59	1	3
South Africa; 2015-2017	9729	4400	0.5	153	263	18	0.1	0
Morocco; 2010-2011	789	789	1	28	30	30	1	1
Zambia; 2011-2013	617	603	1	31	117	108	0.9	6
South Africa; 2011-2013	920	917	1	53	37	37	1	2
South Africa; 2013-2015	··	606	··	30	··	18	··	0
South Africa; 2013-2015	··	842	··	38	··	8	··	1
South Africa; 2009-2012	··	4244	··	235	··	36	··	2

Location (Study period)	All ALRI cases	Tested cases	Proportion of test in ALRI cases	IFV-ALRI cases	All ALRI deaths	Tested deaths	Proportion of test in ALRI deaths	IFV-ALRI deaths
Chile; 2012-2013	481	464	1	36	2	2	1	1
Chile; 2012-2013	683	679	1	31	3	3	1	0

Checklist of information that should be included in new reports of global health estimates

Item #	Checklist item	Reported on page #
**Objectives and funding**		
1	Define the indicator(s), populations (including age, sex, and geographic entities), and time period(s) for which estimates were made.	7-8
2	List the funding sources for the work.	21; summary
**Data Inputs**		
For all data inputs from multiple sources that are synthesized as part of the study:		
3	Describe how the data were identified and how the data were accessed.	7-9; 11
4	Specify the inclusion and exclusion criteria. Identify all ad-hoc exclusions.	7-8
5	Provide information on all included data sources and their main characteristics. For each data source used, report reference information or contact name/institution, population represented, data collection method, year(s) of data collection, sex and age range, diagnostic criteria or measurement method, and sample size, as relevant.	Supplementary material pp 28-56 and pp 19-25
6	Identify and describe any categories of input data that have potentially important biases (e.g., based on characteristics listed in item 5).	15-18
For data inputs that contribute to the analysis but were not synthesized as part of the study:		
7	Describe and give sources for any other data inputs.	7-9, 11; supplementary material pp 19-25
For all data inputs:		
8	Provide all data inputs in a file format from which data can be efficiently extracted (e.g., a spreadsheet rather than a PDF), including all relevant meta-data listed in item 5. For any data inputs that cannot be shared because of ethical or legal reasons, such as third-party ownership, provide a contact name or the name of the institution that retains the right to the data.	We will provide a weblink to Edinburgh Datashare which will be active for public when paper is published.
**Data analysis**		
9	Provide a conceptual overview of the data analysis method. A diagram may be helpful.	A diagram in Figure 1; description in 9-11
10	Provide a detailed description of all steps of the analysis, including mathematical formulae. This description should cover, as relevant, data cleaning, data pre-processing, data adjustments and weighting of data sources, and mathematical or statistical model(s).	9-11; supplementary material pp 19-25, 26, 27.
11	Describe how candidate models were evaluated and how the final model(s) were selected.	10-11
12	Provide the results of an evaluation of model performance, if done, as well as the results of any relevant sensitivity analysis.	Sensitivity analysis in p 13-14; Supplementary material pp 11-18, 19-25.
13	Describe methods for calculating uncertainty of the estimates. State which sources of uncertainty were, and were not, accounted for in the uncertainty analysis.	10, 14
14	State how analytic or statistical source code used to generate estimates can be accessed.	We will provide a weblink which will be active for public when paper is published.
**Results and Discussion**		
15	Provide published estimates in a file format from which data can be efficiently extracted.	We will provide a weblink to Edinburgh Datashare

Item #	Checklist item	Reported on page #
		which will be active for public when paper is published.
**16**	Report a quantitative measure of the uncertainty of the estimates (e.g. uncertainty intervals).	12-14
**17**	Interpret results in light of existing evidence. If updating a previous set of estimates, describe the reasons for changes in estimates.	14-16
**18**	Discuss limitations of the estimates. Include a discussion of any modelling assumptions or data limitations that affect interpretation of the estimates.	14, 16-18

STROBE Statement—checklist of items that should be included in reports of observational studies

	Item No	Recommendation	Reported on page #
**Title and abstract**	1	(*a*) Indicate the study's design with a commonly used term in the title or the abstract	In Title
		(*b*) Provide in the abstract an informative and balanced summary of what was done and what was found	In Summary
**Introduction**			
Background/rationale	2	Explain the scientific background and rationale for the investigation being reported	P9
Objectives	3	State specific objectives, including any prespecified hypotheses	P9
**Methods**			
Study design	4	Present key elements of study design early in the paper	P9
Setting	5	Describe the setting, locations, and relevant dates, including periods of recruitment, exposure, follow-up, and data collection	P9
Participants	6	(*a*) *Cohort study*—Give the eligibility criteria, and the sources and methods of selection of participants. Describe methods of follow-up<br>*Case-control study*—Give the eligibility criteria, and the sources and methods of case ascertainment and control selection. Give the rationale for the choice of cases and controls<br>*Cross-sectional study*—Give the eligibility criteria, and the sources and methods of selection of participants	For meta-analysis, study selection criteria in P9, P10.
		(*b*) *Cohort study*—For matched studies, give matching criteria and number of exposed and unexposed<br>*Case-control study*—For matched studies, give matching criteria and the number of controls per case	Not applicable
Variables	7	Clearly define all outcomes, exposures, predictors, potential confounders, and effect modifiers. Give diagnostic criteria, if applicable	Definition in P11, P12.
Data sources/ measurement	8*	For each variable of interest, give sources of data and details of methods of assessment (measurement). Describe comparability of assessment methods if there is more than one group	Data sources in P9, P11, P13.
Bias	9	Describe any efforts to address potential sources of bias	Inclusion of unpublished data to avoid publication bias (p11);<br>Subgroup and sensitivity analysis in P12, P13.
Study size	10	Explain how the study size was arrived at	Not applicable
Quantitative variables	11	Explain how quantitative variables were handled in the analyses. If applicable, describe which groupings were chosen and why	P11, 12, 13
Statistical methods	12	(*a*) Describe all statistical methods, including those used to control for confounding	P12, 13, 14
		(*b*) Describe any methods used to examine subgroups and interactions	P12
		(*c*) Explain how missing data were addressed	P11, P12
		(*d*) *Cohort study*—If applicable, explain how loss to follow-up was addressed	

Case-control study—If applicable, explain how matching of cases and controls was addressed<br><br>*Cross-sectional study*—If applicable, describe analytical methods taking account of sampling strategy	
(*e*) Describe any sensitivity analyses	P12, P13.

Results			Reported on page#
Participants	13*	(a) Report numbers of individuals at each stage of study—eg numbers potentially eligible, examined for eligibility, confirmed eligible, included in the study, completing follow-up, and analysed	Figure 1; P14
		(b) Give reasons for non-participation at each stage	Figure 1
		(c) Consider use of a flow diagram	Figure 1
Descriptive data	14*	(a) Give characteristics of study participants (eg demographic, clinical, social) and information on exposures and potential confounders	Appendix (PP30-59)
		(b) Indicate number of participants with missing data for each variable of interest	Appendix (PP30-59)
		(c) *Cohort study*—Summarise follow-up time (eg, average and total amount)	Appendix (PP30-59)
Outcome data	15*	*Cohort study*—Report numbers of outcome events or summary measures over time	Appendix (PP7-8)
		Case-control study—Report numbers in each exposure category, or summary measures of exposure	Not applicable
		Cross-sectional study—Report numbers of outcome events or summary measures	Appendix (PP7-8)
Main results	16	(*a*) Give unadjusted estimates and, if applicable, confounder-adjusted estimates and their precision (eg, 95% confidence interval). Make clear which confounders were adjusted for and why they were included	PP14-16; P12
		(*b*) Report category boundaries when continuous variables were categorized	P12
		(*c*) If relevant, consider translating estimates of relative risk into absolute risk for a meaningful time period	Not applicable
Other analyses	17	Report other analyses done—eg analyses of subgroups and interactions, and sensitivity analyses	PP15-16; Appendix PP11-24
**Discussion**			
Key results	18	Summarise key results with reference to study objectives	P16
Limitations	19	Discuss limitations of the study, taking into account sources of potential bias or imprecision. Discuss both direction and magnitude of any potential bias	PP17-20
Interpretation	20	Give a cautious overall interpretation of results considering objectives, limitations, multiplicity of analyses, results from similar studies, and other relevant evidence	P19, PP20-21
Generalisability	21	Discuss the generalisability (external validity) of the study results	P18; PP20-21
**Other information**			
Funding	22	Give the source of funding and the role of the funders for the present study and, if applicable, for the original study on which the present article is based	P23; Summary

*Give information separately for cases and controls in case-control studies and, if applicable, for exposed and unexposed groups in cohort and cross-sectional studies.

Note: An Explanation and Elaboration article discusses each checklist item and gives methodological background and published examples of transparent reporting. The STROBE checklist is best used in conjunction with this article (freely available on the Web sites of PLoS Medicine at http://www.plosmedicine.org/, Annals of Internal Medicine at http://www.annals.org/, and Epidemiology at http://www.epidem.com/). Information on the STROBE Initiative is available at www.strobe-statement.org.

References

1.International Vaccine Access Center (IVAC), Johns Hopkins Bloomberg School of Public Health. VIEW-hub. www.view-hub.org (accessed 5 June 2018).

2.UNAIDS. Global Plan towards the Elimination of New HIV Infections among Children by 2015 and Keeping Their Mothers Alive. Geneva: UNAIDS, 2011.

3.Nguenha N, Tivane A, Pale M, et al. Clinical and epidemiological characterization of influenza virus infections in children with severe acute respiratory infection in Maputo, Mozambique: Results from the implementation of sentinel surveillance, 2014 - 2016. *PLoS One* 2018; **13**(3): e0194138.

4.Liu L, Oza S, Hogan D, et al. Global, regional, and national causes of under-5 mortality in 2000–15: an updated systematic analysis with implications for the Sustainable Development Goals. *The Lancet* 2016; **388**(10063): 3027-35.

5.McAllister DA, Liu L, Shi T, et al. Global, regional, and national estimates of pneumonia morbidity and mortality in children younger than 5 years between 2000 and 2015: a systematic analysis. *The Lancet Global Health* 2018.

6.WHO. Life tables by WHO region. July, 2017 2017 (accessed Sep 12 2017).

7.Sterne JA, White IR, Carlin JB, et al. Multiple imputation for missing data in epidemiological and clinical research: potential and pitfalls. *Bmj* 2009; **338**: b2393.

8.Rubin DB. Multiple imputation for nonresponse in surveys. New York: Wiley; 1987.

9.Honaker J, King G, Blackwell M. Amelia II: A Program for Missing Data. *2011* 2011; **45**(7): 47.

10.Nair H, Brooks WA, Katz M, et al. Global burden of respiratory infections due to seasonal influenza in young children: a systematic review and meta-analysis. *The Lancet* 2011; **378**(9807): 1917-30.

11.Tapia MD, Sow SO, Tamboura B, et al. Maternal immunisation with trivalent inactivated influenza vaccine for prevention of influenza in infants in Mali: a prospective, active-controlled, observer-blind, randomised phase 4 trial. *The Lancet Infectious diseases* 2016; **16**(9): 1026-35.

12.Fowlkes A, Steffens A, Temte J, et al. Incidence of medically attended influenza during pandemic and post-pandemic seasons through the Influenza Incidence Surveillance Project, 2009-13. *The Lancet Respiratory medicine* 2015; **3**(9): 709-18.

13.Heikkinen T, Silvennoinen H, Peltola V, et al. Burden of Influenza in Children in the Community. *The Journal of Infectious Diseases* 2004; **190**(8): 1369-73.

14.Diene Sarr F, Niang M, Thiam D, et al. Acute Febrile Illness and Influenza Disease Burden in a Rural Cohort Dedicated to Malaria in Senegal, 2012–2013. *PLOS ONE* 2015; **10**(12): e0143999.

15.Neuzil KM, Zhu Y, Griffin MR, et al. Burden of interpandemic influenza in children younger than 5 years: a 25-year prospective study. *J Infect Dis* 2002; **185**(2): 147-52.

16.Broor S, Parveen S, Bharaj P, et al. A prospective three-year cohort study of the epidemiology and virology of acute respiratory infections of children in rural India. *PLoS One* 2007; **2**(6): e491.

17.Brooks WA, Goswami D, Rahman M, et al. Influenza is a major contributor to childhood pneumonia in a tropical developing country. *Pediatr Infect Dis J* 2010; **29**(3): 216-21.

18.Horby P, Mai LQ, Fox A, et al. The Epidemiology of Interpandemic and Pandemic Influenza in Vietnam, 2007-2010 The Ha Nam Household Cohort Study I. *American Journal of Epidemiology* 2012; **175**(10): 1062-74.

19.Inamasu T, Sudo K, Kato S, et al. Pandemic Influenza Virus Surveillance, Izu-Oshima Island, Japan. *Emerging Infectious Diseases* 2012; **18**(11): 1882-5.

20.Kimura Y, Saito R, Tsujimoto Y, et al. Geodemographics profiling of influenza A and B virus infections in community neighborhoods in Japan. *BMC Infectious Diseases* 2011; **11**(1): 36.

21.Regamey N, Kaiser L, Roiha HL, et al. Viral etiology of acute respiratory infections with cough in infancy: a community-based birth cohort study. *Pediatr Infect Dis J* 2008; **27**(2): 100-5.

22.Kumar P, Medigeshi GR, Mishra VS, et al. Etiology of Acute Respiratory Infections in Infants: A Prospective Birth Cohort Study. *Pediatric Infectious Disease Journal* 2017; **36**(1): 25-30.

23.Teros-Jaakkola T, Toivonen L, Schuez-Havupalo L, et al. Influenza virus infections from 0 to 2 years of age: A birth cohort study. *Journal of Microbiology, Immunology and Infection* 2017.
24.Buck PO, Smith DM, Shenolikar R, Irwin DE. A Retrospective Cohort Study of the Incidence, Health Care Resource Utilization and Costs of International Classification of Diseases, Clinical Modification, 9th Revision Diagnosed Influenza and Related Complications in US Children. *Pediatric Infectious Disease Journal* 2017; **36**(12): 1129-40.
25.Li-Kim-Moy JP, Yin JK, Heron L, et al. Influenza vaccine efficacy in young children attending childcare: A randomised controlled trial. *Journal of Paediatrics & Child Health* 2017; **53**(1): 47-54.
26.Steinhoff MC, Katz J, Englund JA, et al. Year-round influenza immunisation during pregnancy in Nepal: a phase 4, randomised, placebo-controlled trial. *The Lancet Infectious Diseases* 2017; **17**(9): 981-9.
27.Sarna M, Lambert SB, Sloots TP, et al. Viruses causing lower respiratory symptoms in young children: findings from the ORChID birth cohort. *Thorax* 2018; **73**(10): 969-79.
28.Gefenaite G, Pistol A, Popescu R, et al. Estimating burden of influenza-associated influenza-like illness and severe acute respiratory infection at public healthcare facilities in Romania during the 2011/12-2015/16 influenza seasons. *Influenza Other Respir Viruses* 2018; **12**(1): 183-92.
29.Katz J, Englund JA, Steinhoff MC, et al. Impact of Timing of Influenza Vaccination in Pregnancy on Transplacental Antibody Transfer, Influenza Incidence, and Birth Outcomes: A Randomized Trial in Rural Nepal. *Clinical infectious diseases : an official publication of the Infectious Diseases Society of America* 2018; **67**(3): 334-40.
30.Oliva J, Delgado-Sanz C, Larrauri A. Estimating the burden of seasonal influenza in Spain from surveillance of mild and severe influenza disease, 2010-2016. *Influenza Other Respir Viruses* 2018; **12**(1): 161-70.
31.Forster J, Ihorst G, Rieger CHL, et al. Prospective population-based study of viral lower respiratory tract infections in children under 3 years of age (the PRI.DE study). *European Journal of Pediatrics* 2004; **163**(12): 709-16.
32.Hasan R, Rhodes J, Thamthitiwat S, et al. Incidence and etiology of acute lower respiratory tract infections in hospitalized children younger than 5 years in rural Thailand. *Pediatr Infect Dis J* 2014; **33**(2): e45-52.
33.Tallo VL, Kamigaki T, Tan AG, et al. Estimating influenza outpatients' and inpatients' incidences from 2009 to 2011 in a tropical urban setting in the Philippines. *Influenza & Other Respiratory Viruses* 2014; **8**(2): 159-68.
34.Yu H, Huang J, Huai Y, et al. The substantial hospitalization burden of influenza in central China: surveillance for severe, acute respiratory infection, and influenza viruses, 2010-2012. *Influenza & Other Respiratory Viruses* 2014; **8**(1): 53-65.
35.Chaves SS, Perez A, Farley MM, et al. The burden of influenza hospitalizations in infants from 2003 to 2012, United States. *Pediatric Infectious Disease Journal* 2014; **33**(9): 912-9.
36.Chiu SS, Lo JY, Chan K-H, et al. Population-based hospitalization burden of influenza a virus subtypes and antigenic drift variants in children in Hong Kong (2004–2011). *PloS one* 2014; **9**(4): e92914.
37.Nelson EAS, Ip M, Tam JS, et al. Burden of influenza infection in hospitalised children below 6 months of age and above in Hong Kong from 2005 to 2011. *Vaccine* 2014; **32**(49): 6692-8.
38.Dawood FS, Fiore A, Kamimoto L, et al. Burden of seasonal influenza hospitalization in children, United States, 2003 to 2008. *J Pediatr* 2010; **157**(5): 808-14.
39.Weigl JA, Puppe W, Belke O, Neususs J, Bagci F, Schmitt HJ. The descriptive epidemiology of severe lower respiratory tract infections in children in Kiel, Germany. *Klinische Padiatrie* 2005; **217**(5): 259-67.
40.Montes M, Vicente D, Perez-Yarza EG, Cilla G, Perez-Trallero E. Influenza-related hospitalisations among children aged less than 5 years old in the Basque Country, Spain: a 3-year study (July 2001-June 2004). *Vaccine* 2005; **23**(34): 4302-6.

41.Ajayi-Obe EK, Coen PG, Handa R, et al. Influenza A and respiratory syncytial virus hospital burden in young children in East London. *Epidemiology and infection* 2008; **136**(8): 1046-58.
42.Nicholson KG, McNally T, Silverman M, Simons P, Stockton JD, Zambon MC. Rates of hospitalisation for influenza, respiratory syncytial virus and human metapneumovirus among infants and young children. *Vaccine* 2006; **24**(1): 102-8.
43.Yoshida LM, Suzuki M, Yamamoto T, et al. Viral pathogens associated with acute respiratory infections in central vietnamese children. *Pediatr Infect Dis J* 2010; **29**(1): 75-7.
44.Nelson EA, Tam JS, Yu LM, Li AM, Chan PK, Sung RY. Assessing disease burden of respiratory disorders in Hong Kong children with hospital discharge data and linked laboratory data. *Hong Kong medical journal = Xianggang yi xue za zhi* 2007; **13**(2): 114-21.
45.Chiu SS, Chan KH, Chen H, et al. Virologically confirmed population-based burden of hospitalization caused by influenza A and B among children in Hong Kong. *Clinical infectious diseases : an official publication of the Infectious Diseases Society of America* 2009; **49**(7): 1016-21.
46.Ji W, Zhang T, Zhang X, et al. The epidemiology of hospitalized influenza in children, a two year population-based study in the People's Republic of China. *BMC health services research* 2010; **10**: 82.
47.Poehling KA, Edwards KM, Weinberg GA, et al. The Underrecognized Burden of Influenza in Young Children. *New England Journal of Medicine* 2006; **355**(1): 31-40.
48.Iwane MK, Edwards KM, Szilagyi PG, et al. Population-based surveillance for hospitalizations associated with respiratory syncytial virus, influenza virus, and parainfluenza viruses among young children. *Pediatrics* 2004; **113**(6): 1758-64.
49.Ampofo K, Gesteland PH, Bender J, et al. Epidemiology, complications, and cost of hospitalization in children with laboratory-confirmed influenza infection. *Pediatrics* 2006; **118**(6): 2409-17.
50.Coffin SE, Zaoutis TE, Rosenquist ABW, et al. Incidence, complications, and risk factors for prolonged stay in children hospitalized with community-acquired influenza. *Pediatrics* 2007; **119**(4): 740-8.
51.Grijalva CG, Craig AS, Dupont WD, et al. Estimating Influenza Hospitalizations among Children. *Emerging Infectious Diseases* 2006; **12**(1): 103-9.
52.Kamigaki T, Aldey PP, Mercado ES, et al. Estimates of influenza and respiratory syncytial virus incidences with fraction modeling approach in Baguio City, the Philippines, 2012-2014. *Influenza and other Respiratory Viruses* 2017; **11**(4): 311-8.
53.Li-Kim-Moy J, Yin JK, Patel C, et al. 2016 Australian vaccine preventable disease epidemiological review series: Influenza 2006 to 2015. *Communicable Diseases Intelligence Quarterly Report* 2016; **40**(4): E482-E95.
54.Jain S, Williams DJ, Arnold SR, et al. Community-acquired pneumonia requiring hospitalization among US children. *New England Journal of Medicine* 2015; **372**(9): 835-45.
55.Sakkou Z, Stripeli F, Papadopoulos NG, et al. Impact of influenza infection on children's hospital admissions during two seasons in Athens, Greece. *Vaccine* 2011; **29**(6): 1167-72.
56.Silvennoinen H, Peltola V, Vainionpaa R, Ruuskanen O, Heikkinen T. Incidence of influenza-related hospitalizations in different age groups of children in Finland: A 16-year study. *Pediatric Infectious Disease Journal* 2011; **30**(2): e24-e8.
57.Yousey-Hindes KM, Hadler JL. Neighborhood socioeconomic status and influenza hospitalizations among children: New Haven County, Connecticut, 2003–2010. *American journal of public health* 2011; **101**(9): 1785-9.
58.Homaira N, Luby SP, Hossain K, et al. Respiratory viruses associated hospitalization among children aged <5 years in Bangladesh: 2010-2014. *PLoS ONE* 2016; **11**(2): e0147982.
59.Al-Awaidy S, Hamid S, Al Obaidani I, et al. The Burden of Influenza-Associated Hospitalizations in Oman, January 2008-June 2013. *PLOS ONE* 2015; **10**(12): e0144186.
60.Descalzo MA, Clara W, Guzmán G, et al. Estimating the burden of influenza‐associated hospitalizations and deaths in Central America. *Influenza and other respiratory viruses* 2016; **10**(4): 340-5.

61.Rojo JC, Ruiz-Contreras J, Fernández MB, Marín MA, Folgueira L. Influenza-Related Hospitalizations in Children Younger Than Three Years of Age. *The Pediatric Infectious Disease Journal* 2006; **25**(7): 596-601.
62.D'Onise K, Raupach JC. The burden of influenza in healthy children in South Australia. *The Medical journal of Australia* 2008; **188**(9): 510-3.
63.Henrickson KJ, Hoover S, Kehl KS, Hua W. National disease burden of respiratory viruses detected in children by polymerase chain reaction. *Pediatr Infect Dis J* 2004; **23**(1 Suppl): S11-8.
64.Sutmoller F, Ferro ZP, Asensi MD, Ferreira V, Mazzei IS, Cunha BL. Etiology of acute respiratory tract infections among children in a combined community and hospital study in Rio de Janeiro. *Clinical infectious diseases : an official publication of the Infectious Diseases Society of America* 1995; **20**(4): 854-60.
65.Ntiri MP, Duque J, McMorrow ML, et al. 2016 Incidence of medically attended influenza among residents of Shai-Osudoku and Ningo-Prampram Districts, Ghana, May 2013 - April 2015. *BMC Infectious Diseases* 2016; **16**(1): 757.
66.Boddington NL, Verlander NQ, Pebody RG. Developing a system to estimate the severity of influenza infection in England: findings from a hospital-based surveillance system between 2010/2011 and 2014/2015. *Epidemiology & Infection* 2017; **145**(7): 1461-70.
67.Von Der Beck D, Seeger W, Herold S, Gunther A, Loh B. Characteristics and outcomes of a cohort hospitalized for pandemic and seasonal influenza in Germany based on nationwide inpatient data. *PLoS ONE* 2017; **12**(7): e0180920.
68.Poehling KA, Edwards KM, Griffin MR, et al. The burden of influenza in young children, 2004-2009. *Pediatrics* 2013; **131**(2): 207-16.
69.Harvala H, Smith D, Salvatierra K, et al. Burden of influenza B virus infections in Scotland in 2012/13 and epidemiological investigations between 2000 and 2012. *Euro Surveillance: Bulletin Europeen sur les Maladies Transmissibles = European Communicable Disease Bulletin* 2014; **19**(37).
70.Olabarrieta I, Gonzalez-Carrasco E, Calvo C, Pozo F, Casas I, Garcia-Garcia ML. Hospital admission due to respiratory viral infections in moderate preterm, late preterm and term infants during their first year of life. *Allergologia et Immunopathologia* 2015; **43**(5): 469-73.
71.Chaves SS, Aragon D, Bennett N, et al. Patients hospitalized with laboratory-confirmed influenza during the 2010-2011 influenza season: Exploring disease severity by virus type and subtype. *Journal of Infectious Diseases* 2013; **208**(8): 1305-14.
72.Babakazo P, Lubula L, Disasuani W, et al. The national and provincial burden of medically attended influenza-associated influenza-like illness and severe acute respiratory illness in the Democratic Republic of Congo, 2013-2015. *Influenza Other Respir Viruses* 2018; **12**(6): 695-705.
73.Ieng V, Tolosa MX, Tek B, et al. National burden of influenza-associated hospitalizations in Cambodia, 2015 and 2016. *WESTERN PACIFIC SURVEILLANCE AND RESPONSE* 2018; **9**.
74.Nyamusore J, Rukelibuga J, Mutagoma M, et al. The national burden of influenza-associated severe acute respiratory illness hospitalization in Rwanda, 2012-2014. *Influenza and Other Respiratory Viruses* 2018; **12**(1): 38-45.
75.Sotomayor V, Fasce RA, Vergara N, De la Fuente F, Loayza S, Palekar R. Estimating the burden of influenza-associated hospitalizations and deaths in Chile during 2012-2014. *Influenza Other Respir Viruses* 2018; **12**(1): 138-45.
76.Susilarini NK, Haryanto E, Praptiningsih CY, et al. Estimated incidence of influenza-associated severe acute respiratory infections in Indonesia, 2013-2016. *Influenza Other Respir Viruses* 2018; **12**(1): 81-7.
77.Yi Z, David JM, Quanyi W, et al. Hospitalizations for Influenza-Associated Severe Acute Respiratory Infection, Beijing, China, 2014–2016. *Emerging Infectious Disease journal* 2018; **24**(11): 2098.
78.赵小娟, 张奕, 杨剑, 田兴军, 王保东 李. 怀柔区流感住院病例特征及其住院率估计. *国际病毒学杂志* 2018; **25**(4): 281.
79.Abdel-Hady DM, Al Balushi RM, Al Abri BA, et al. Estimating the burden of influenza-associated hospitalization and deaths in Oman (2012-2015). *Influenza Other Respir Viruses* 2018; **12**(1): 146-52.

80.Bonmarin I, Belchior E, Bergounioux J, et al. Intensive care unit surveillance of influenza infection in France: the 2009/10 pandemic and the three subsequent seasons. *Euro Surveillance: Bulletin Europeen sur les Maladies Transmissibles = European Communicable Disease Bulletin* 2015; **20**(46).
81.Kaczmarek MC, Ware RS, Coulthard MG, McEniery J, Lambert SB. Epidemiology of Australian Influenza-Related Paediatric Intensive Care Unit Admissions, 1997-2013. *Plos One* 2016; **11**(3).
82.Gubbels S, Krause TG, Bragstad K, Perner A, Molbak K, Glismann S. Burden and characteristics of influenza A and B in Danish intensive care units during the 2009/10 and 2010/11 influenza seasons. *Epidemiology & Infection* 2013; **141**(4): 767-75.
83.Chuang JH, Huang AS, Huang WT, et al. Nationwide surveillance of influenza during the pandemic (2009-10) and post-pandemic (2010-11) periods in Taiwan. *PLoS ONE* 2012; **7**(4): e36120.
84.Kwong KL, Lung D, Wong SN, Que TL, Kwong NS. Influenza-related hospitalisations in children. *Journal of paediatrics and child health* 2009; **45**(11): 660-4.
85.Moore DL, Vaudry W, Scheifele DW, et al. Surveillance for influenza admissions among children hospitalized in Canadian immunization monitoring program active centers, 2003-2004. *Pediatrics* 2006; **118**(3): e610-9.
86.Coelho MC, Tsuchiya LR, Nogueira MB, et al. Impact of respiratory infections by influenza viruses A and B in pediatrics patients from Federal University of Parana, Brazil. *The Brazilian journal of infectious diseases : an official publication of the Brazilian Society of Infectious Diseases* 2007; **11**(2): 220-3.
87.Sam IC, Abdul-Murad A, Karunakaran R, et al. Clinical features of Malaysian children hospitalized with community-acquired seasonal influenza. *International journal of infectious diseases : IJID : official publication of the International Society for Infectious Diseases* 2010; **14** (Suppl 3): e36-40.
88.Bashir U, Nisar N, Arshad Y, et al. Respiratory syncytial virus and influenza are the key viral pathogens in children <2 years hospitalized with bronchiolitis and pneumonia in Islamabad Pakistan. *Archives of Virology* 2017; **162**(3): 763-73.
89.Kanik A, Eliacik K, Koyun B, et al. 2016 Viral Etiology of Acute Bronchiolitis in Hospitalized Infants and the Effect on Clinical Course. *Journal of Pediatric Infection* 2016; **10**(3): 93-8.
90.Li-Kim-Moy J, Yin JK, Blyth CC, et al. Influenza hospitalizations in Australian children. *Epidemiology & Infection* 2017; **145**(7): 1451-60.
91.Acar M, Sutcu M, Akturk H, et al. Clinical differences of influenza subspecies among hospitalized children. [Turkish]. *Turk Pediatri Arsivi* 2017; **52**(1): 15-22.
92.Horton KC, Dueger EL, Kandeel A, et al. Viral etiology, seasonality and severity of hospitalized patients with severe acute respiratory infections in the Eastern Mediterranean Region, 2007-2014. *PloS one* 2017; **12**(7): e0180954-e.
93.Dananche C, Sanchez Picot V, Benet T, et al. Burden of Influenza in Less Than 5-Year-Old Children Admitted to Hospital with Pneumonia in Developing and Emerging Countries: A Descriptive, Multicenter Study. *The American journal of tropical medicine and hygiene* 2018; **98**(6): 1805-10.
94.Draganescu A, Sandulescu O, Florea D, et al. The influenza season 2016/17 in Bucharest, Romania - surveillance data and clinical characteristics of patients with influenza-like illness admitted to a tertiary infectious diseases hospital. *The Brazilian journal of infectious diseases : an official publication of the Brazilian Society of Infectious Diseases* 2018; **22**(5): 377-86.
95.El Omeiri N, Azziz-Baumgartner E, Thompson MG, et al. Seasonal influenza vaccine effectiveness against laboratory-confirmed influenza hospitalizations - Latin America, 2013. *Vaccine* 2018; **36**(24): 3555-66.
96.Machablishvili A, Chakhunashvili G, Zakhashvili K, et al. Overview of three influenza seasons in Georgia, 2014–2017. *PLOS ONE* 2018; **13**(7): e0201207.
97.Torner N, Martínez A, Basile L, et al. Descriptive study of severe hospitalized cases of laboratory-confirmed influenza during five epidemic seasons (2010–2015). *BMC Research Notes* 2018; **11**(1): 244.
98.张锐沐. 住院儿童流感后肺炎 152 例临床特征分析 [硕士]: 汕头大学; 2016.

99.朱芮, 甘雨茹, 盛鄂湘, 赵东赤. 武汉市某医院 2016-2017 年流行性感冒流行病学与临床特征分析. *武汉大学学报(医学版)* 2018; **39**(4).
